# Estimating the basic reproduction number of measles in low-and middle-income settings using 172 seroprevalence studies: A modelling approach

**DOI:** 10.1371/journal.pgph.0006731

**Published:** 2026-07-17

**Authors:** Han Fu, Alyssa N. Sbarra, Timothy Russell, Kaja Abbas, Megan Auzenbergs, Mark Jit

**Affiliations:** 1 Department of Infectious Disease Epidemiology and Dynamics, London School of Hygiene and Tropical Medicine, United Kingdom; 2 Department of Epidemiology, Johns Hopkins University, United States of America; 3 School of Tropical Medicine and Global Health, Nagasaki University, Japan; 4 Institute of Tropical Medicine, Nagasaki University, Japan; 5 Public Health Foundation of India, New Delhi, India; 6 National Institute of Infectious Diseases, Japan Institute for Health Security, Tokyo, Japan; 7 School of Global Public Health, New York University, United States of America; 8 Saw Swee Hock School of Public Health, National University of Singapore, Singapore; 9 School of Public Health, The University of Hong Kong, Hong Kong SAR, People’s Republic of China; University of Washington, UNITED STATES OF AMERICA

## Abstract

The basic reproduction number, R_0_, is an epidemiological measure to describe the transmissibility of infectious diseases and evaluate the potential effect of interventions. Measles R_0_ has historically been considered to be between 12–18, with contextual factors contributing to its heterogeneity, but has rarely been estimated using data across multiple countries. Our study aims to estimate measles R_0_ in low- and middle-income countries using a standardised database of population-based serosurveys. We fitted an age-structured compartmental model of measles transmission dynamics and vaccination (DynaMICE) to the age-specific seroprevalence data extracted from a recent systematic review. Using Markov Chain Monte Carlo, we estimated setting-specific posterior distributions of R_0_ in 172 studies with unique survey years and locations from 57 countries. Bootstrapped samples of R_0_ estimates were pooled by study characteristics, including survey period, geography, and overall bias in sampling, measurement, and reporting results. Measles R_0_ estimates varied substantially across serostudies, ranging from 0.93 (95% credible interval (CrI): 0.70–1.00) to 147 (95% CrI: 76.5–208), with fewer than 13% of studies having median R_0_ values in the range of 12–18. Pooled R_0_ estimates showed smaller medians and variation in serostudies conducted after 2000 or including the adult population, while no distinguishable variation was identified across the World Health Organization regions. Our revised estimates demonstrated the wide range of measles R_0_ across low- and middle-income settings and highlighted the importance of considering the heterogeneity in measles transmissibility when modelling epidemics and planning interventions and vaccination strategies.

## Introduction

Measles is a contagious disease prone to epidemics and has resulted in a large health burden worldwide [1,2]. The basic reproduction number (R_0_) of measles is generally recognised as one of the highest amongst common vaccine-preventable diseases, with suggestions that each primary measles case can generate 12–18 secondary infections when the population is completely susceptible [3]. Due to this characteristic of high transmissibility, measles requires a high level of two-dose vaccine coverage for disease control. However, a systematic review of R_0_ estimates reveals that measles R_0_ varied widely from 1.43 to 770 by country settings [4]. This variation in measles transmissibility can affect the estimation of measles burden and vaccine impact in individual settings. However, it was unclear the extent to which this variation stemmed from the diversity in data sources or estimation methods across studies, as opposed to true heterogeneity.

Serosurveys examine the presence of specific antibodies to understand the immunity profile against infectious diseases within a population over a defined period of time. Because presence of these measles-specific antibodies is understood to be a correlate of protection from the clinical disease, seroprevalence data stratified by age, vaccination history, and other selected characteristics can be used to inform susceptibility gaps in the population and understand the heterogeneity of transmissibility across settings. With appropriate sampling and data collection methods, serosurveys provide a less biased snapshot of the population-level immunity profiles in the community, compared to notification data that are subject to under-ascertainment and underreporting issues in the surveillance systems [5].

To address the heterogeneity of measles transmissibility across low- and middle-income countries (LMICs) that contribute to the majority of global measles burden [2], we extracted age-specific seroprevalence data from serostudies included in a recently conducted systematic review [6]. We then inferred the setting-specific R_0_ by fitting a dynamic model of measles transmission and vaccination to the seroprevalence dataset [7–9]. The comparison of model-based R_0_ estimates by setting-specific characteristics will enhance the understanding of the key factors that contribute to the heterogeneity of measles transmissibility across settings.

## Methods

### Serological data

We obtained 221 articles from a 2024 systematic review by Sbarra et al. that summarised the scope and bias in population-level measles serosurveys [6]. Surveys that did not provide information on the age distribution of the sampled population (n = 16) were excluded. We also excluded survey data from non-representative populations, such as immunocompromised patients, pre-term babies, and refugees or border populations (n = 21). Based on the bias assessment in the previous review [6], we further excluded surveys having a critical bias in the validity of the tools used to measure serology (n = 19). We did not exclude serosurveys that were rated lower quality because they had insufficient information when reporting serological results, because a standard reporting style was not widely used, especially in early studies, and because reporting completeness would not necessarily reflect the implementation quality of serosurveys.

From the 165 included articles, a total of 172 unique studies were defined with a unique combination of country, survey year, and first author (Table A in S1 Appendix). We extracted 13 unique studies from 6 articles, including survey data for multiple years or multiple countries. Where only the publication years were available, we approximated the survey years using the average duration between the survey and publication years in studies with complete information (3.7 years). In mother-newborn studies examining both the samples from mother-newborn pairs, we extracted the data only from mothers. Most such studies reported a high correlation in the antibody concentration between mother and newborn. The age-specific seroprevalence estimates were derived from surveys conducted between 1963 and 2019 in 57 LMICs, with the majority found in China, India, Türkiye, and Brazil (Fig A in S1 Appendix). Most of the included studies (n = 132, 76.7%) consisted of seroprevalence estimates across multiple age groups, and only a small proportion did not cover children (n = 21, 12.2%).

### Measles transmission and vaccine impact model

Dynamic Measles Immunization Calculation Engine (DynaMICE) is a compartmental model developed to capture measles transmission and assess the health impact of vaccination strategies at the country level. The model consists of four epidemiological states – maternally immune, susceptible, infectious, and recovered (Fig B in S1 Appendix), further stratified by the doses of measles-containing vaccines (MCV) received. DynaMICE is incorporated with fine-scale age structure, including 156 weekly age groups for children aged under 3 years old and 98 yearly age groups for those aged between 3 and 100 years old. In addition, modelling the force of measles infection takes into account annual seasonality via a sine function, pathogen transmissibility via R_0_, and age-dependent mixing pattern via a contact matrix. We assumed the incubation period for measles to be 14 days and immunity following infection to be lifelong [10]. The proportion of newborns with maternal immunity was directly informed by the proportion of immune adults at child-bearing age (18–35 years old, ‘recovered’ compartments). We derived country-specific demographics from the United Nations World Population Prospects [11]. Country-specific synthetic contact matrices were obtained from Prem et al. [12] and modified to match the age structure of DynaMICE.

In modelling measles vaccination in DynaMICE, we applied country-specific vaccination schedules and coverage reported in the World Health Organization (WHO) Immunization Data portal [1]. Routine MCV1 and MCV2 doses were provided at the country-specific recommended age, and supplementary immunisation activities were implemented as one-off campaigns in the targeted age groups in the middle of the calendar year. We assumed vaccine effectiveness for MCV1 to vary linearly by age [13], two-dose vaccine protection to be capped at 98% [14], and no further protection from three or more MCV doses. Details of model structure, equations, parameters, and key assumptions have been included in the previous publication [8].

### Model fitting

We fitted DynaMICE to each unique set of age-specific seroprevalence data per country-time period using Markov Chain Monte Carlo (MCMC) with component-wise Metropolis algorithm and random order for updating parameters in each blocked iteration [15]. Four MCMC chains for each study were conducted with starting parameters randomly drawn from their prior distributions. Three parameters―R_0_, duration of maternal immunity protection, and vaccine effectiveness for the first MCV dose―were fitted, and they were assumed to be constant over time within the same serostudy. We selected less informative prior parameter distributions to cover a variety of settings for the 172 included serostudies (Table 1). We extracted prior distributions for R_0_ and vaccine effectiveness from the systematic reviews [4,13] that gathered all the available evidence, and for the duration of maternal immunity, we used information from a longitudinal study with multiple follow-ups [16]. For 69 serosurveys conducted prior to country-specific MCV introduction, we removed the parameter for vaccine effectiveness and fitted the seroprevalence data with a two-parameter model. We constructed the likelihood by assuming the seroprevalence in each age group to be independently binomially distributed. We conducted diagnostic checks for convergence of MCMC chains and goodness-of-fit, with a targeting effective sample size above 200. We obtained 500 posterior samples for each study and calculated the medians and 95% credible intervals (CrI) of fitted parameters.

**Table 1:** Prior distribution of parameters for model fitting.

Estimated parameter	Prior distribution, median (5^th^–95^th^ percentiles)	Source and note
Basic reproduction number (R_0_)	Log-normal, 13.1 (5.44–31.8)	Guerra et al. [4]
Duration of maternal immunity, months	Truncated log-normal, 2.84 (1.24–6.45) minimum: 1, maximum: 12	Leuridan et al. [16] Fitted to multiple time points in the longitudinal study
Vaccine effectiveness at 9 months old (only for surveys conducted in the post-vaccination era)	Truncated normal, 77.5% (49.3%-100%) minimum: 26%, maximum: 100%	Hughes et al. [13] Fitted to the reported range and further capped by two-dose effectiveness 98%

In our model-based outputs, we added up the non-susceptible populations ― maternally immune, infectious, and recovered sub-populations (compartments) [8] ― to calculate the seroprevalence in the corresponding age groups and calendar years. We included all the non-susceptible states because IgG antibodies, as the detection target in serosurveys, cannot distinguish between maternally acquired, infection-acquired and vaccine-induced immunity. Models were first simulated using the country-specific demographics in 1980 [11] and assumed no implementation of vaccination until they reached equilibrium. From 1980 to the survey year, we applied annual inputs of demographics and MCV coverage estimates [1] (Fig C in S1 Appendix). For serosurveys conducted before 1980, we extracted the model estimates in 1980, considering that seroprevalence was likely similar during earlier years, given low coverage of vaccination and other control measures for measles.

### Factors associated with R_0_

Measles R_0_ is influenced by demographic, socioeconomic, and environmental factors, which change contact behaviours and transmission risk per contact in the population [4]. Although the DynaMICE model has accounted for country-specific age structure and age-dependent contact patterns, other factors could further contribute to the temporal and geographical variation in measles R_0_. In addition, the survey quality and available details of seroprevalence data also affect the estimation of R_0_ through fitting to seroprevalence data. To understand these dependencies, we evaluated the association between R_0_ estimates and different study features, including survey period, age of the survey population, number of age-specific data points, and potential methodological biases. We used the bias assessment outcomes in Sbarra et al. [6], which evaluated the processes of sample selection, assay protocol, handling of equivocal results, and other information on serology testing methodology presented in the original serostudies under systematic, pre-defined algorithms. Serostudies were categorised into low, moderate, severe, and critical levels of biases in sampling, measurement and reporting serology. Unweighted bootstrapping was used to pool R_0_ estimates and compare their distributions across the selected study features. For each selected feature, we filtered a subset of serostudies, and from their posterior samples, we randomly drew 10,000 bootstrapped samples with replacement.

We assessed the association of median R_0_ for each study with average household size [17], population density [11], and Human Development Index (HDI) [18]. HDI is a country-specific and time-dependent summary measure combining the dimensions of health and longevity, education, and living standard. When these data were missing at specific survey years, we performed linear interpolation for household size and for HDI and population density, we used the estimate at the closest year on a country basis.

### Estimated measles cases

Using the posterior parameters of R_0_, duration of maternal immunity, and vaccine effectiveness, we estimated the annual numbers of measles cases during the survey year and the two years before and after. We calculated the mean annual cases over the five years to reduce the influence of year-to-year fluctuations in the time series. We compared the model-based case estimates with the WHO country-specific reported cases [19] to assess their consistency in measuring measles burden and the potential issue of underreporting. We also compared our model-based estimates with measles incidence estimates from the Global Burden of Disease (GBD) Study 2021 [20], as it is a key publicly available resource for health decision makers.

### Sensitivity analysis

We investigated the goodness-of-fit of the two-parameter and three-parameter models by calculating the root mean square error (RMSE) between the observed seroprevalence data and median posterior predictions. As the number of age data points is different in each serostudy, the RMSE metric enables comparison across studies by averaging the total error size. To assess how model fit affects the estimation of R_0_, we ranked the RMSE and conducted a sensitivity analysis on the pooled R_0_ results after removing 20% of the studies with the highest error size.

## Results

### Posterior R_0_ estimates

We conducted MCMC diagnostics and presented parameter trace plots, prior and posterior parameter distributions, pairwise parameter correlations, effective sample sizes, and Gelman-Rubin statistics in Figs D–G in S1 Appendix. We obtained 500 sets of posterior parameters from the converged MCMC chains. The posterior model estimates of age-specific seroprevalence show a good fit using the RMSE measure (Fig H in S1 Appendix), with two-thirds of the studies having an average difference less than 10% between the model results and observed data (S1 Fig). There was a positive correlation between median R_0_ and vaccine effectiveness estimates (Spearman’s correlation coefficient (R)=0.3007, p-value (p)=0.0020). However, the correlation became weaker after excluding studies with a severe or critical bias in study population selection, serological measurement and result reporting (Fig I in S1 Appendix, R = 0.2558, p = 0.0485). No significant correlation between R_0_ and duration of maternal immunity was found (R = 0.0122, p = 0.8739).

**Fig 1:**
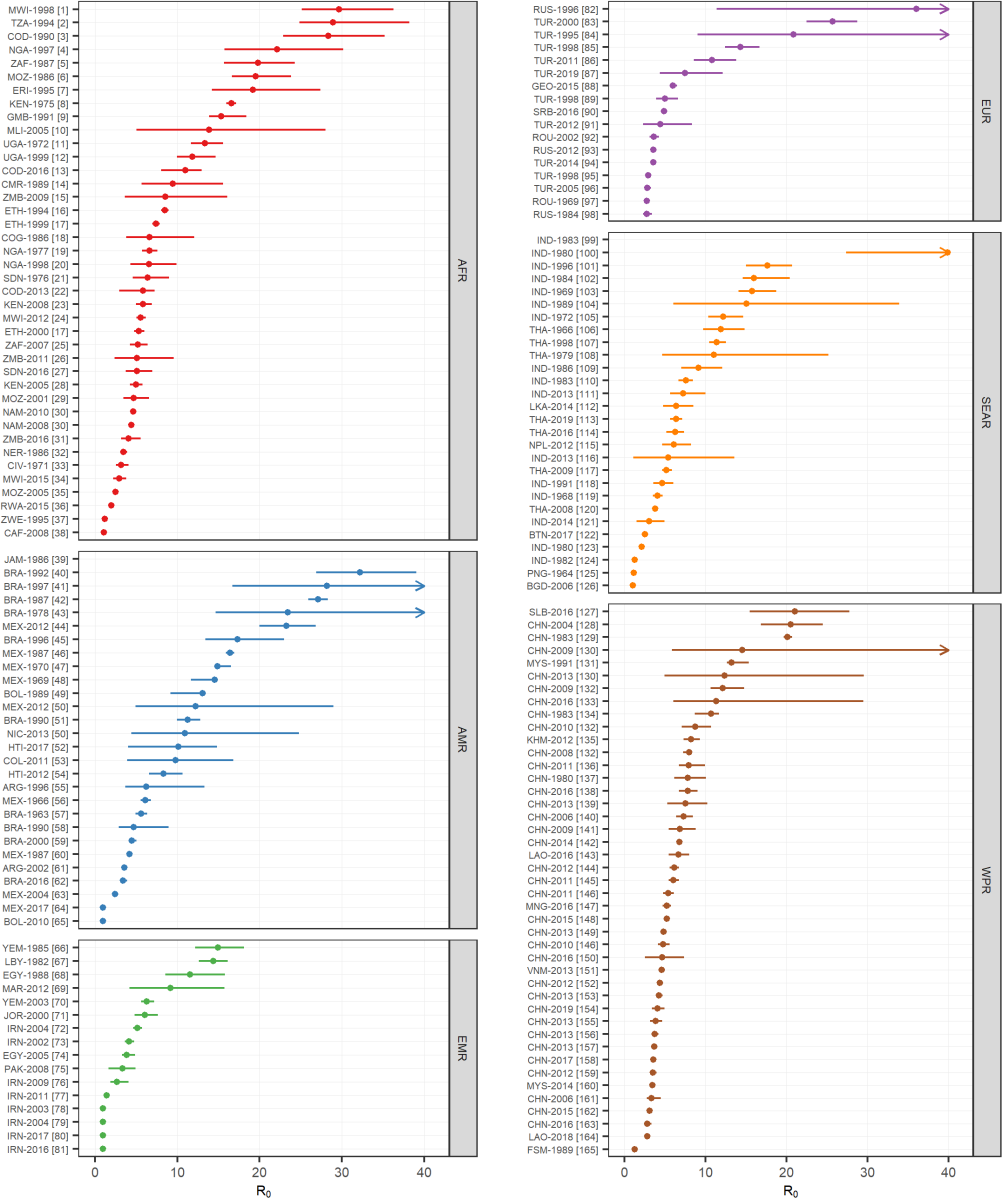
Posterior measles R_0_ obtained by fitting to age-specific seroprevalence data. The median posterior R_0_ estimates are presented with 95% credical intervals, ordered from highest to lowest in each WHO region. Arrows indicate that the upper R_0_ estimates exceed 40. Two studies (JAM-1986 and IND-1983) with the lower bound estimate above 40 are not shown. See the study reference list in Table A in S1 Appendix.

Fig 1 presents the median and 95% CrIs of the R_0_ posterior distributions obtained from fitting the DynaMICE model to age-specific seroprevalence data in 172 individual studies. We observed a right skewed distribution of R_0_ estimates, ranging widely across studies from 0.93 (95% CrI: 0.70–1.00) to 147 (95%CrI: 76.5–208). Only 12.8% (n = 22) of the median estimates were in the commonly cited range of 12–18 [3], while 75.0% (n = 129) of them presented a value below 12 and 12.2% (n = 21) above 18. In 15.2% of the included studies (n = 26), posterior R_0_ showed a broad range of 95% CrIs, with the difference between lower and upper bounds exceeding 10. These R_0_ estimates with a broad range tended to result from serosurveys with a smaller sample size, fewer age-specific data points, or conducted in the pre-vaccination era. Geographically, R_0_ estimates concentrated in a range of less than 15 based on serosurveys conducted in the WHO Eastern Mediterranean Region, while in other regions, a few upper R_0_ estimates could reach 30 and above. The variation in R_0_ estimates remained large between studies in the same country, as seen in median R_0_ estimates dispersing over a range of 20 in China, India, Brazil, and Türkiye (Fig J in S1 Appendix).

### Factors associated with R_0_

**Fig 2:**
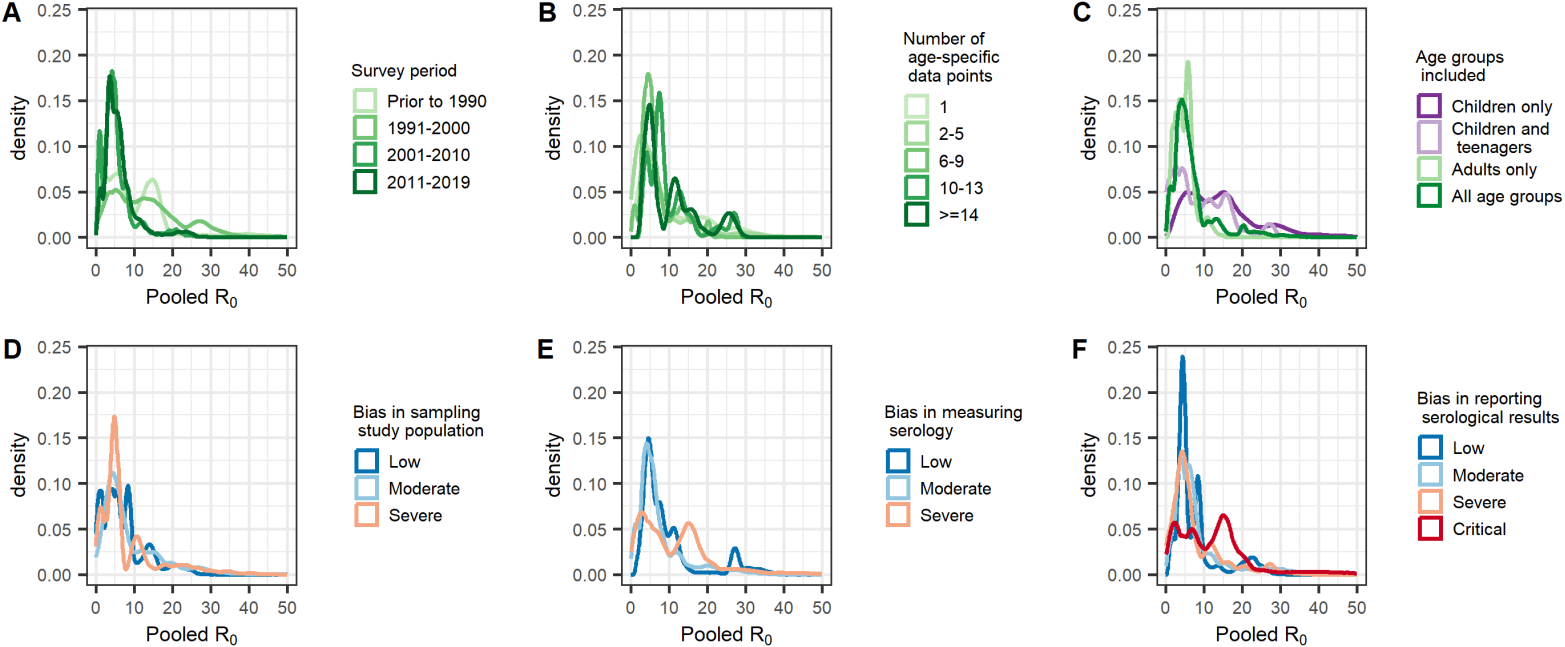
Pooled measles R_0_ by (A) survey period, (B) number of age-specific data points per survey, (C) age groups of survey population, (D) bias in sampling study population, (E) bias in measuring serology, and (F) bias in reporting serological results. Density of 10,000 bootstrapped R_0_ estimates by each selected feature is presented.

Across different study periods, age groups, and bias levels of serosurveys, pooled R_0_ estimates mostly presented a right-skewed distribution and often peaked at a value <10 (Fig 2). However, R_0_ estimates based on the same study feature could still have wide variation, as seen in the multimodal curves of pooled R_0_ estimates in studies with a larger number of seroprevalence data points and a lower bias in sampling, measuring, and reporting measles serology. Our findings suggested that the survey period and age of populations may be associated with the peaks and shapes of pooled R_0_ distributions. The median R_0_ estimates were 8.99 (interquartile range (IQR)=10.4) in the serostudies conducted prior to 1990, 12.8 (IQR = 14.0) in 1991–2000, 4.48 (IQR = 3.62) in 2001–2010, and 5.17 (IQR = 3.73) in 2011–2019, showing a smaller value and narrower variability in the estimates based on more recent studies. A wider variation and a greater value were also found in R_0_ estimates based on studies involving only children (median = 13.2, IQR = 11.6) compared to those with both children and adults (median = 5.19, IQR = 4.42). We also inspected six serostudies reporting R_0_ below 1 and found they were conducted in near-elimination settings where MCV1 coverage level tended to be high and close to the overall seropositivity in the population. On the other hand, two serostudies with the highest R_0_ values (>50) were conducted in the pre-vaccination era, where high seroprevalence in very young children was observed. The factors affecting the overall variation in R_0_ remained important when we looked at influential factors for variation within countries. However, across different countries, the associated factors often had different directions of effect on R_0_ (Fig K in S1 Appendix).

**Fig 3:**
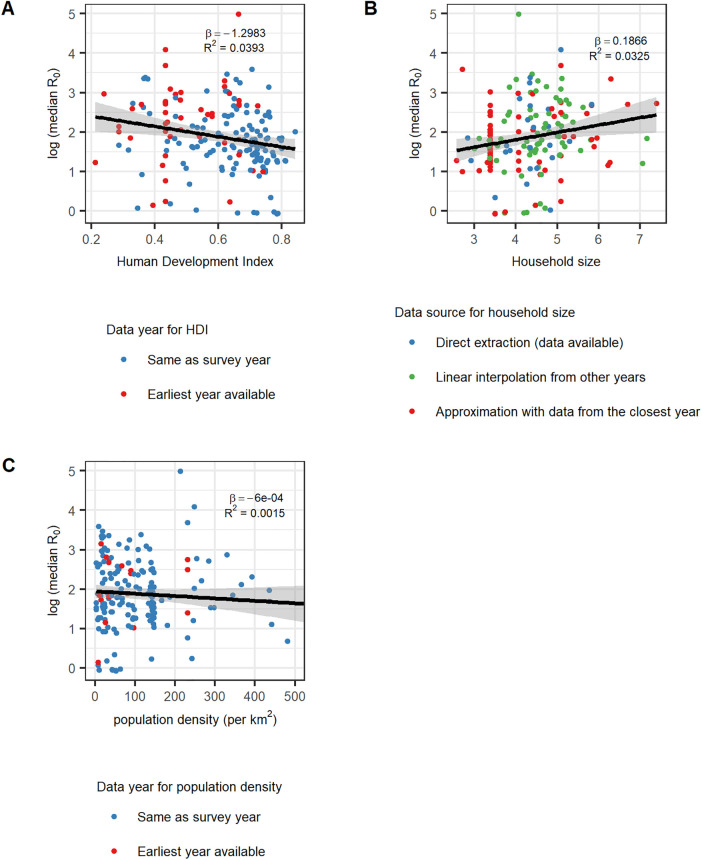
Median R_0_ in relation to (A) Human Development Index, (B) household size and (C) population density. Regression coefficients and R-squared metrics were derived based on univariable log-linear models. Where available, data were matched to the year each serosurvey was conducted.

In exploring other country- and year-specific factors in the broader context, we identified a weak negative correlation (R = –0.2787, p = 0.0002) between the posterior median R_0_ and Human Development Index, suggesting that measles transmissibility decreased with improvement in health and socioeconomics. We also found a weak positive correlation between median R_0_ and average household size (R = 0.2437, p = 0.0015). Nonetheless, no significant correlation was found with population density (R = –0.0361, p = 0.6385). Based on the univariable log-linear regression analysis on R_0_, HDI was a significant predictor but with limited explanation of the variance (R-squared = 3.93%, Fig 3).

### Estimated measles cases

WHO data on reported measles cases was not available, especially in the early years when case surveillance and notification systems had not yet been fully operated. Among 155 studies included for comparison (Fig L in S1 Appendix), 60% of our median model-based estimates (n = 93) show greater numbers than the WHO reported cases. Assuming these model-based estimates reflect the actual magnitude of measles incidence, we found that a proportion of 1.27% measles cases were reported to WHO (25^th^–75^th^ percentiles: 0.56%–2.94%). However, in 36 studies for comparison, the upper bound model estimates reveal < 100 cases, while the WHO data reported > 1000 cases. We also noted that the majority of serostudies where model-estimated measles cases were lower than the WHO-reported cases were conducted in China (n = 23) and having severe bias in measuring and reporting serological results.

The comparison with the GBD 2021 Study was available for 134 serostudies (77.9%) conducted between 1988–2019 (Fig M in S1 Appendix). Our model-based case estimates generally differed from the GBD incidence estimates, with 84 serostudies (62.7%) having uncertainty intervals that did not overlap with those from GBD. Compared to GBD, our model was more likely to present lower case estimates and the scale of difference in median estimates was notable (lower by >80% for all 62 serostudies with non-overlapping interval estimates).

### Sensitivity analysis

**Fig 4:**
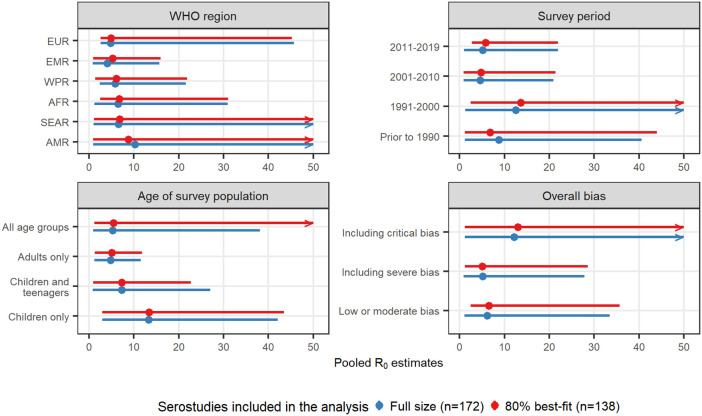
Pooled measles R_0_ estimates in the full set of studies and the restricted set with a better model fit. Median and 95% credible intervals of pooled R_0_ estimates from studies with each selected feature are presented. Arrows indicate the upper estimates exceed 50.

Fitting the DynaMICE model to the age-specific seroprevalence data, we presented a median RMSE of 6.16% (25^th^–75^th^ percentiles: 2.40%–11.6%). Those serostudies with a poorer fit were with more age-specific data points for fitting, conducted in earlier years, and less likely to have low or moderate bias in overall serosurvey quality, but no particular trend was seen by WHO regions (Table B in S1 Appendix). We conducted a sensitivity analysis restricting the analysis to the 80% best-fitting studies, with RMSEs below 15.2%. Compared to the main analysis with a full set of studies, pooled R_0_ estimates of the restricted set generally showed similar point estimates and wide intervals for WHO regions, study periods, age groups, and serological measurement and reporting biases (Fig 4).

## Discussion

This analysis systematically estimated measles R_0_ using published age-specific serological data across LMICs. Based on dynamic model simulation of measles transmission and vaccination, we identified great variability in R_0_ across 172 study settings. Our model-based measles R_0_ estimates were mostly below the commonly cited range between 12–18 and demonstrated large heterogeneity even within the same countries or WHO regions. The value of R_0_ tended to be higher in serosurveys conducted prior to 2000 or including only the children population, and it was also found to be negatively associated with the Human Development Index. Furthermore, the serology-informed R_0_ estimates we applied to quantify the measles incidence suggested a low reporting of disease burden in the surveillance systems.

The great variability in measles R_0_ identified in our analysis was also reported in a comprehensive review by Guerra et al. [4]. Both our analysis and Guerra’s review found that most R_0_ estimates to be outside of the commonly cited range of 12–18, which was derived from a single study taking early data in England and New York [3]. However, Guerra et al. [4] summarised estimates from independent modelling studies based on surveillance and/or serology data sources, and it was therefore not able to separate to what extent the R_0_ variability could be attributed to the methodological differences. Another multi-country analysis based on the same model structure reported more than five times greater measles transmission rates in the United Kingdom than in Ethiopia, but only two LMICs were included, and the homogenous mixing assumption limited further inspection of the heterogeneity of transmissibility [21]. Instead, our analysis included all the available serological data in LMICs conducted prior to 2019 and utilised the DynaMICE model incorporated with fine-scale age structure and country-specific demographics and contact patterns. With a systematic framework for serosurvey selection and model parameterisation, we improved the quantification of measles R_0_ heterogeneity and assessment of potential contributing factors.

Advancements in healthcare, education and living standards could partly explain the variability in measles R_0_, as suggested in previous studies and our regression analysis with HDI [4]. However, we did not find a correlation between R_0_ and average household size and population density. In DynaMICE, the effect of household structure and crowdedness on age-related mixing across countries may have been partially adjusted through the incorporation of synthetic social contact matrices, which were constructed with contact diary surveys and demography data [8,22]. In such cases, other indicators like clustering of susceptibility or proportion of large-sized households could be more informative to explain the complicated mechanism of household transmission [23,24]. In addition to selecting appropriate indicators, addressing the interactions between them is essential to disentangle the heterogeneity in measles transmissibility. An advanced understanding of factors associated with the R_0_ heterogeneity will help characterise measles transmission in settings where serodata are unavailable [23] and update disease burden under evolving settings.

We estimated annual numbers of measles cases by leveraging the strength of serological data in capturing disease incidence regardless of symptom occurrence and access to healthcare [25]. The comparison between our serology-based burden estimates and WHO notification data suggested that fewer than 5% of cases were reported in most study settings. Underreporting was also identified in a previous modelling study [26], and the WHO notification data were prone to underestimation due to low propensity to seek care in settings with weak healthcare systems, low case ascertainment, preferential reporting of severe cases, and variable surveillance sensitivity. We also compared our measles case numbers with GBD 2021, the only publicly available incidence estimates at the country level. We found limited alignment in the magnitude of disease burden between the two studies, largely owing to methodological differences―the GBD estimates were derived from a mixed-effects linear regression model with routine and campaign MCV coverage as covariates and fitted to the WHO notification data [27]. As our seroprevalence-based case estimates are subject to several limitations, the comparison with the WHO and GBD estimates of measles cases should be seen as exploratory only.

Our study has several limitations. First, we assumed that each serosurvey was nationally representative and used country-level demography and vaccine coverage inputs for model fitting. However, serosurveys were usually conducted in geographically restricted populations due to time, cost, and personnel constraints, especially in LMICs [28]. Whilst high-spatial-resolution estimates of routine MCV coverage are available for addressing subnational heterogeneity [29], local data on campaign coverage, contact patterns, and population structure are still limited in LMICs. In many serostudies included in our analysis, reported information was insufficient in defining the geographic areas of the catchment population [6]. A mismatch of population representativeness could lead to either overestimation or underestimation of measles R_0_, as subnational coverage can be greater or lower than national coverage (Fig N in S1 Appendix). The interaction between coverage and other model inputs could further complicate any prediction on the direction of bias in R_0_ estimation. Nonetheless, our exploratory analysis of a small number of serostudies (n = 44) showed that the pooled R_0_ estimates did not change substantially when we removed serostudies in settings with >10% difference between national and subnational coverage levels (Fig O in Appendix).

Second, we assumed that R_0_ and social contact rates were constant over time. Our pooled R_0_ analysis by study period suggested a potential temporal trend. Assuming a constant R_0_ could underestimate measles transmissibility in the years before 2000 but overestimate it in the later years. Even though we addressed some of the temporal effects in DynaMICE through yearly inputs of population age structure, social and environmental factors can still contribute to temporal variation in measles transmissibility. For example, factors such as urbanisation, education, infrastructure improvement, and major public health events like COVID-19 may alter measles transmission and contact behaviour in particular settings [4,30]. The temporal trend of R_0_ is likely to be non-linear, sensitive to occasional events, and jointly driven by multiple time-varying components. Repeated cross-sectional serosurveys in the same population could facilitate the characterisation of the temporal trends in measles transmissibility.

Third, the serostudies included in our analysis used different laboratory methods and antibody levels to define seropositivity, in part likely due to weak evidence for an antibody threshold correlating to protection against measles infection [31]. Although we excluded studies with a critical bias in measuring seropositivity [6], we were not able to consider the sensitivity and specificity of the laboratory methods because the original serostudies gave limited information on whether laboratory protocols were specifically followed, and on sample quality at time of testing. We also did not perform sensitivity analyses across studies that used different thresholds to define seroprotection. Should information on serological test performance be incorporated into model fitting, the updated R_0_ estimates could change in either direction, depending on the relative values of test sensitivity and specificity. In addition to the performance of serological tools, serosurveys with sufficient age-specific data points and a broad age range (e.g., covering both children and adults) will help address the changing vaccination strategies over time and reduce bias and uncertainty in estimating measles R_0_.

Fourth, we did not consider the waning of measles IgG antibodies in the population, which could lead to underestimation of R_0_ in matching seroprevalence in older adults. However, existing observations showed that the decrease in IgG antibody concentration is slow and hence will likely have a small effect on seroprevalence [32]. In LMICs, where measles transmission is mostly endemic, high-level immunity in the population could be sustained with infection. Thus, the effect of immunity waning in adults is less of a concern in most of the serostudies included in this analysis, except for those in the near-elimination settings.

In conclusion, we have comprehensively explored measles R_0_ across LMICs by fitting to the available age-specific seroprevalence data extracted from a systematic review, accounting for country-specific demographic characteristics, contact patterns, and immunisation programmes with the DynaMICE model. We have illustrated the significant variability in measles R_0_ estimates across different settings. We demonstrated that the variation in R_0_ is not purely driven by differences in model structure, but also dependent on serosurvey features and country-specific demographic and developmental characteristics. Further research to improve the quality, granularity, and completeness of data collection for serostudies and other model inputs will enhance the estimation of measles R_0_ and understanding of its key determinants. Our findings highlight the importance of heterogeneities at the country level and subnational level for planning measles interventions and vaccination strategies in LMICs. Local R_0_ values are crucial to identify the speed at which epidemics spread and the level of immunity needed to prevent transmission. Setting-specific serological survey data should be used to support epidemiological analyses and decision-making wherever available.

## Supporting information

S1 Appendix Supplementary methods and results. (PDF)

S1 Fig Observed data and model estimates of age-specific seroprevalence. Study reference is included in S1 Appendix.
(PDF)
