# Supplementary material for: Estimating the basic reproduction number of measles in low-and middle-income settings using 172 seroprevalence studies: A modelling approach
Source: PLOS Glob Public Health. 2026 Jul 17;6(7):e0006731. doi: 10.1371/journal.pgph.0006731 (PMC13378968; doi:10.1371/journal.pgph.0006731)
Supplement: S1 Appendix — (PDF) [file pgph.0006731.s001.pdf]

S1 Appendix

Estimating the basic reproduction number of measles in low-and middle-income settings using 172 seroprevalence studies: a modelling approach

Han Fu, Alyssa Sbarra, Timothy Russell, Kaja Abbas, Megan Auzenbergs, Mark Jit

Contents

Table A. Characteristics and references of the 172 included studies........ 2
Fig A. Geographical distributions of serological studies included (n=172) 6
Fig B. Module structure of Dynamic Measles Immunization Calculation Engine (DynaMICE) 7
Fig C. Time horizon and trajectories of simulation........ 8
Fig D. Trace plots of posterior log-likelihood and parameters 9
Fig E. Prior and posterior parameter distributions........ 11
Fig F. Pairwise correlation matrix between fitting parameters 13
Fig G. Effective sample size and Gelman-Rubin statistic........ 17
Fig H. Root mean square error of age-specific seroprevalence data 18
Fig I. Correlations between posterior R_0 and vaccine effectiveness and duration of maternal immunity........ 19
Fig J. Fitted measles R_0 using age-specific seroprevalence data in China, India, Brazil, and Türkiye........ 20
Fig K. Factors associated with pooled R_0 estimates in China, India, Brazil, and Türkiye 21
Fig L. Model-estimated and WHO reported measles cases at the year of serosurvey (n=155) 22
Fig M. Model estimates and Global Burden of Diseases 2021 cases at the year of serosurvey (n=134) 23
Table B. RSMEs by selected study features........ 24
Fig N. Difference between national and subnational coverage at survey years (n=44) 25
Fig O. Pooled R_0 in serostudies with subnational coverage data........ 26
References........ 27

Table A. Characteristics and references of the 172 included studies

WHO region	Country code	Survey year	First author	Level of bias selection	measurement	reporting	Reference
AFR	MWI	1998	Takechi	moderate	moderate	moderate	[1]
AFR	TZA	1994	Lyamuya	moderate	moderate	severe	[2]
AFR	COD	1990	Cutts	moderate	severe	severe	[3]
AFR	NGA	1997	Oyefolu	moderate	severe	critical	[4]
AFR	ZAF	1987	De Swardt	moderate	moderate	severe	[5]
AFR	MOZ	1986	Cutts	moderate	moderate	moderate	[6]
AFR	ERI	1995	Tolfvenstam	moderate	moderate	severe	[7]
AFR	KEN	1975	Voorhoeve	moderate	severe	severe	[8]
AFR	GMB	1991	Fortuin	moderate	severe	moderate	[9]
AFR	MLI	2005	Tapia	moderate	low	low	[10]
AFR	UGA	1972	Munube	moderate	severe	critical	[11]
AFR	UGA	1999	Waibale	moderate	low	severe	[12]
AFR	COD	2016	Keating	moderate	moderate	moderate	[13]
AFR	CMR	1989	Ndumbe	moderate	low	moderate	[14]
AFR	ZMB	2009	Lowther	low	moderate	severe	[15]
AFR	ETH	1994	Enquselassie	low	moderate	low	[16]
AFR	ETH	1999	Nigatu	moderate	severe	severe	[17]
AFR	COG	1986	Dabis	moderate	low	low	[18]
AFR	NGA	1977	Ogunmekan	moderate	severe	critical	[19]
AFR	NGA	1998	Hartter	moderate	low	severe	[20]
AFR	SDN	1976	Omer	moderate	severe	critical	[21]
AFR	COD	2013	Ashbaugh	moderate	moderate	moderate	[22]
AFR	KEN	2008	Merkel	moderate	moderate	severe	[23]
AFR	MWI	2012	Polonsky	moderate	moderate	severe	[24]
AFR	ETH	2000	Nigatu	moderate	severe	severe	[17]
AFR	ZAF	2007	Jallow	moderate	moderate	severe	[25]
AFR	ZMB	2011	Sutcliffe	moderate	low	moderate	[26]
AFR	SDN	2016	Adam	moderate	moderate	moderate	[27]
AFR	KEN	2005	Scott	severe	moderate	severe	[28]
AFR	MOZ	2001	Mandomando	moderate	moderate	low	[29]
AFR	NAM	2010	Cardemil	moderate	low	low	[30]
AFR	NAM	2008	Cardemil	moderate	low	low	[30]
AFR	ZMB	2016	Hayford	low	moderate	low	[31]
AFR	NER	1986	Loutan	moderate	severe	moderate	[32]
AFR	CIV	1971	Breman	moderate	severe	critical	[33]
AFR	MWI	2015	Polonsky	moderate	moderate	severe	[34]
AFR	MOZ	2005	Jani	moderate	severe	moderate	[35]
AFR	RWA	2015	Seruyange	moderate	moderate	moderate	[36]
AFR	ZWE	1995	Obi	moderate	severe	critical	[37]
AFR	CAF	2008	Manirakiza	moderate	moderate	severe	[38]
AMR	JAM	1986	Christie	moderate	low	moderate	[39]
AMR	BRA	1992	Oliveira	moderate	low	moderate	[40]
AMR	BRA	1997	Lindgren-Alves	moderate	moderate	moderate	[41]

AMR	BRA	1987	Pannuti	moderate	low	severe	[42]
AMR	BRA	1978	Baruzzi	severe	severe	critical	[43]
AMR	MEX	2012	Díaz-Ortega	moderate	moderate	low	[44]
AMR	BRA	1996	Godoy	moderate	moderate	critical	[45]
AMR	MEX	1987	Sepúlveda	moderate	severe	critical	[46]
AMR	MEX	1970	Ruiz-Gomez	moderate	severe	critical	[47]
AMR	MEX	1969	Gutiérrez	moderate	severe	critical	[48]
AMR	BOL	1989	Guglielmetti	moderate	severe	critical	[49]
AMR	MEX	2012	Colson	moderate	moderate	moderate	[50]
AMR	BRA	1990	Cox	moderate	low	severe	[51]
AMR	NIC	2013	Colson	moderate	moderate	moderate	[50]
AMR	HTI	2017	Minta	low	moderate	low	[52]
AMR	COL	2011	González	moderate	moderate	moderate	[53]
AMR	HTI	2012	Fitter	moderate	low	low	[54]
AMR	ARG	1996	Nates	moderate	moderate	low	[55]
AMR	MEX	1966	Golubjatnikov	moderate	severe	critical	[56]
AMR	BRA	1963	Niederman	severe	moderate	critical	[57]
AMR	BRA	1990	de Moraes-Pinto	moderate	severe	critical	[58]
AMR	BRA	2000	Castro-Silva	moderate	low	severe	[59]
AMR	MEX	1987	Fajardo-Gutiérrez	moderate	moderate	severe	[60]
AMR	ARG	2002	Dayan	moderate	moderate	low	[61]
AMR	BRA	2016	Estofolete	moderate	moderate	severe	[62]
AMR	MEX	2004	Belaunzarán-Zamudio	moderate	moderate	moderate	[63]
AMR	MEX	2017	Sánchez-Aleman	moderate	moderate	moderate	[64]
AMR	BOL	2010	Masuet-Aumatell	moderate	moderate	severe	[65]
EMR	YEM	1985	Strauss	moderate	severe	critical	[66]
EMR	LBY	1982	El Nageh	moderate	severe	critical	[67]
EMR	EGY	1988	Fathy	moderate	moderate	severe	[68]
EMR	MAR	2012	Haban	moderate	moderate	moderate	[69]
EMR	YEM	2003	Sallam	moderate	moderate	moderate	[70]
EMR	JOR	2000	Bdour	moderate	moderate	moderate	[71]
EMR	IRN	2004	Esteghamati	moderate	moderate	severe	[72]
EMR	IRN	2002	Karimi	moderate	moderate	severe	[73]
EMR	EGY	2005	Abbassy	moderate	moderate	severe	[74]
EMR	PAK	2008	Sheikh	moderate	severe	severe	[75]
EMR	IRN	2009	Khaki	moderate	moderate	severe	[76]
EMR	IRN	2011	Honarvar	moderate	moderate	low	[77]
EMR	IRN	2003	Yekta	low	moderate	moderate	[78]
EMR	IRN	2004	Pourabbas	moderate	moderate	severe	[79]
EMR	IRN	2017	Zahraei	low	moderate	severe	[80]
EMR	IRN	2016	Izadi	moderate	moderate	severe	[81]
EUR	RUS	1996	Nikitiuk	moderate	severe	critical	[82]
EUR	TUR	2000	Gozalan	moderate	moderate	moderate	[83]
EUR	TUR	1995	Ozbek	moderate	moderate	severe	[84]
EUR	TUR	1998	Egemen	low	moderate	severe	[85]
EUR	TUR	2011	Tanriover	severe	moderate	severe	[86]

EUR	TUR	2019	Karaayvaz	moderate	moderate	severe	[87]
EUR	GEO	2015	Khetsuriani	moderate	moderate	moderate	[88]
EUR	TUR	1998	Metintaş	moderate	moderate	severe	[89]
EUR	SRB	2016	Ristić	moderate	moderate	low	[90]
EUR	TUR	2012	Bekdas	moderate	moderate	severe	[91]
EUR	ROU	2002	Brumboiu	moderate	moderate	moderate	[92]
EUR	RUS	2012	Kostinov	moderate	moderate	moderate	[93]
EUR	TUR	2014	Emek	low	low	moderate	[94]
EUR	TUR	1998	Kanra	moderate	severe	critical	[95]
EUR	TUR	2005	Gunes	moderate	moderate	severe	[96]
EUR	ROU	1969	Sorodoc	moderate	severe	critical	[97]
EUR	RUS	1984	Mal'tseva	moderate	moderate	severe	[98]
SEAR	IND	1983	Bhaskaram	moderate	severe	critical	[99]
SEAR	IND	1980	Cherian	moderate	severe	critical	[100]
SEAR	IND	1996	Khan	moderate	severe	critical	[101]
SEAR	IND	1984	Khare	moderate	severe	critical	[102]
SEAR	IND	1969	John	moderate	severe	critical	[103]
SEAR	IND	1989	Dongre	moderate	severe	critical	[104]
SEAR	IND	1972	Broor	moderate	severe	critical	[105]
SEAR	THA	1966	Ueda	moderate	moderate	severe	[106]
SEAR	THA	1998	Saipan	moderate	moderate	severe	[107]
SEAR	THA	1979	Vanprapar	moderate	severe	critical	[108]
SEAR	IND	1986	Bhardwaj	moderate	moderate	critical	[109]
SEAR	IND	1983	Sehgal	moderate	severe	critical	[110]
SEAR	IND	2013	Gohil	moderate	moderate	moderate	[111]
SEAR	LKA	2014	Muthiah	moderate	moderate	moderate	[112]
SEAR	THA	2019	Wanlapakorn	moderate	moderate	moderate	[113]
SEAR	THA	2016	Chaiwarith	moderate	moderate	severe	[114]
SEAR	NPL	2012	Murray	moderate	moderate	severe	[115]
SEAR	IND	2013	Sathiyanarayanan	moderate	moderate	severe	[116]
SEAR	THA	2009	Tharmaphornpilas	moderate	moderate	severe	[117]
SEAR	IND	1991	Sood	moderate	severe	moderate	[118]
SEAR	IND	1968	Mehta	moderate	moderate	moderate	[119]
SEAR	THA	2008	Gonwong	severe	moderate	moderate	[120]
SEAR	IND	2014	Gupta	moderate	moderate	moderate	[121]
SEAR	BTN	2017	Wangchuk	low	low	moderate	[122]
SEAR	IND	1980	Saha	moderate	severe	critical	[123]
SEAR	IND	1982	Sharma	moderate	severe	critical	[124]
SEAR	PNG	1964	Willis	moderate	severe	critical	[125]
SEAR	BGD	2006	Sultana	moderate	moderate	severe	[126]
WPR	SLB	2016	Breakwell	low	moderate	low	[127]
WPR	CHN	2004	Yu	moderate	moderate	severe	[128]
WPR	CHN	1983	Zhang	moderate	severe	critical	[129]
WPR	CHN	2009	Chong	moderate	moderate	moderate	[130]
WPR	MYS	1991	Saraswathy	moderate	moderate	severe	[131]
WPR	CHN	2013	Chong	moderate	moderate	moderate	[130]
WPR	CHN	2009	Liu	moderate	moderate	moderate	[132]

WPR	CHN	2016	Zhu	moderate	moderate	moderate	[133]
WPR	CHN	1983	Hao	moderate	severe	critical	[134]
WPR	CHN	2010	Liu	moderate	moderate	moderate	[132]
WPR	KHM	2012	Mao	low	moderate	low	[135]
WPR	CHN	2008	Liu	moderate	moderate	moderate	[132]
WPR	CHN	2011	Wang	moderate	moderate	severe	[136]
WPR	CHN	1980	Xu	moderate	severe	critical	[137]
WPR	CHN	2016	Pei	moderate	moderate	moderate	[138]
WPR	CHN	2013	Ma	moderate	moderate	moderate	[139]
WPR	CHN	2006	Ma	moderate	low	severe	[140]
WPR	CHN	2009	He	moderate	moderate	moderate	[141]
WPR	CHN	2014	Ding	moderate	moderate	moderate	[142]
WPR	LAO	2016	Khampanisong	moderate	moderate	moderate	[143]
WPR	CHN	2012	Ma	moderate	moderate	severe	[144]
WPR	CHN	2011	Xiong	low	moderate	moderate	[145]
WPR	CHN	2011	Chong	moderate	moderate	moderate	[146]
WPR	MNG	2016	Nogareda	low	low	moderate	[147]
WPR	CHN	2015	Zhao	moderate	moderate	severe	[148]
WPR	CHN	2013	Wagner	moderate	moderate	moderate	[149]
WPR	CHN	2010	Chong	moderate	moderate	moderate	[146]
WPR	CHN	2016	Yan	moderate	moderate	severe	[150]
WPR	VNM	2013	Choisy	moderate	moderate	moderate	[151]
WPR	CHN	2012	Li	moderate	moderate	severe	[152]
WPR	CHN	2013	Boulton	moderate	moderate	low	[153]
WPR	CHN	2019	Wang	moderate	moderate	low	[154]
WPR	CHN	2013	Han	moderate	moderate	severe	[155]
WPR	CHN	2013	Lu	moderate	moderate	severe	[156]
WPR	CHN	2013	Fu	moderate	moderate	moderate	[157]
WPR	CHN	2017	Zhang	moderate	moderate	moderate	[158]
WPR	CHN	2012	Zhang	moderate	moderate	severe	[159]
WPR	MYS	2014	Hazlina	moderate	moderate	moderate	[160]
WPR	CHN	2006	Xu	moderate	moderate	severe	[161]
WPR	CHN	2015	Tang	moderate	moderate	severe	[162]
WPR	CHN	2016	Meng	moderate	moderate	low	[163]
WPR	LAO	2018	Xaydalasouk	moderate	moderate	severe	[164]
WPR	FSM	1989	Withers	severe	severe	moderate	[165]

Fig A. Geographical distributions of serological studies included (n=172)

The base layer of this world map was derived from Natural Earth, version 2.0, 1:50m (see https://www.naturalearthdata.com/about/terms-of-use/ for the terms of use).

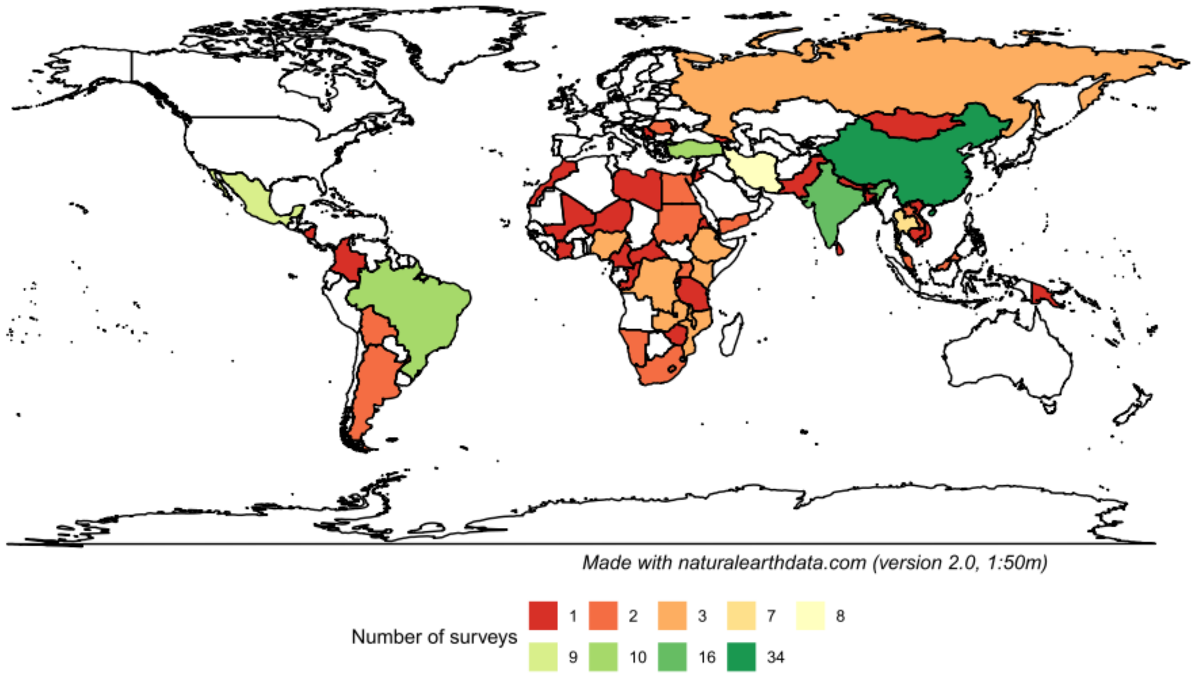

Fig B. Module structure of Dynamic Measles Immunization Calculation Engine (DynaMICE)

DynaMICE consists of four epidemiological states (M-maternally immune, S-susceptible, I-infectious, R-recovered) and records the vaccination history (V1-1 dose, V2-2 doses, V3-≥3 doses). Arrows denote the transition between two states over the transmission, vaccination, and progression of measles. Only a single age group is included for clarity of presentation. This figure is derived from Fu et al. [166], with CC BY licence permitting for unrestricted use with citation.

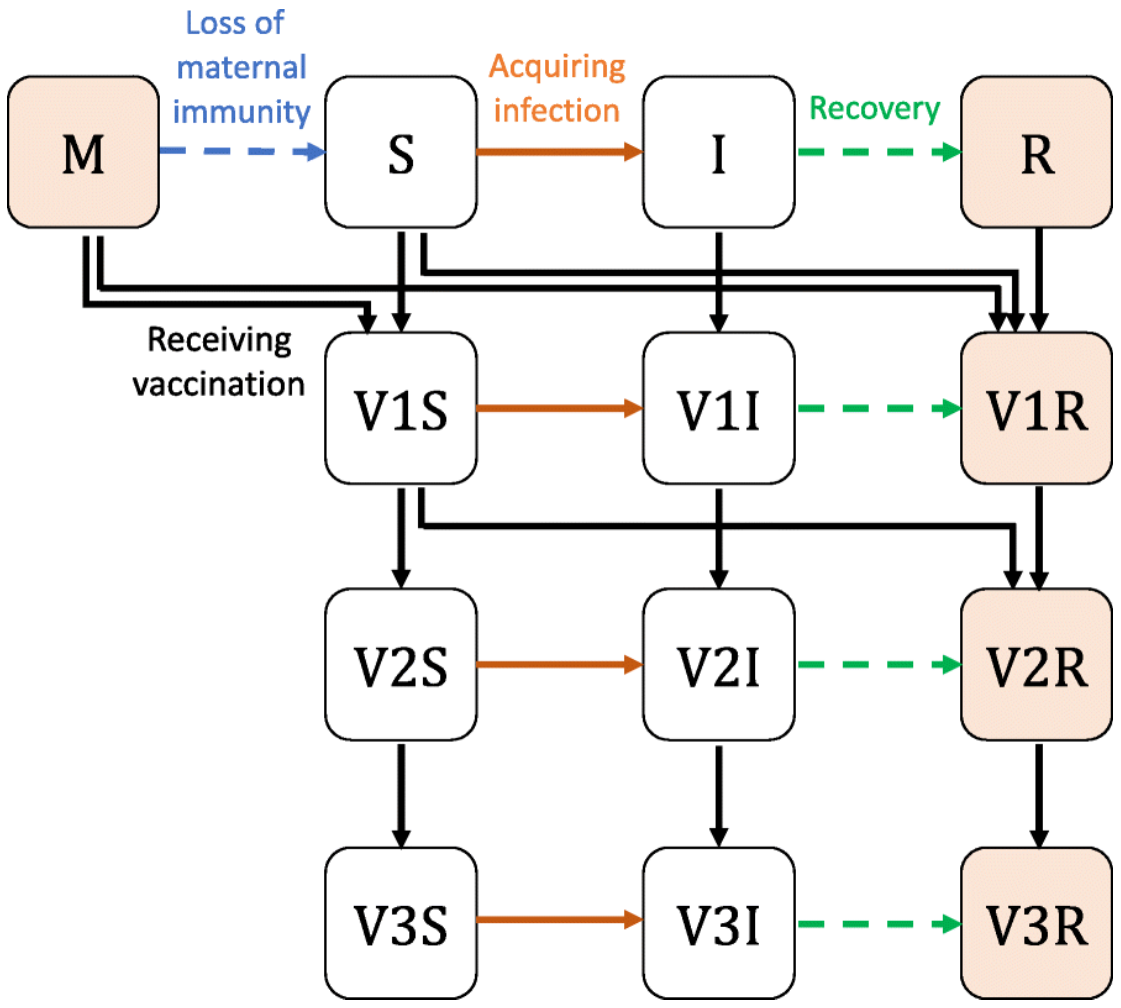

Fig C. Time horizon and trajectories of simulation

Each model run contains an equilibrium period of 100 years, followed by annual inputs of country demography and vaccine coverage from 1980 to the year of serosurvey conducted. Trajectories of measles incidence (grey solid lines) in Argentina [55] based on 500 sets of posterior parameters are presented.

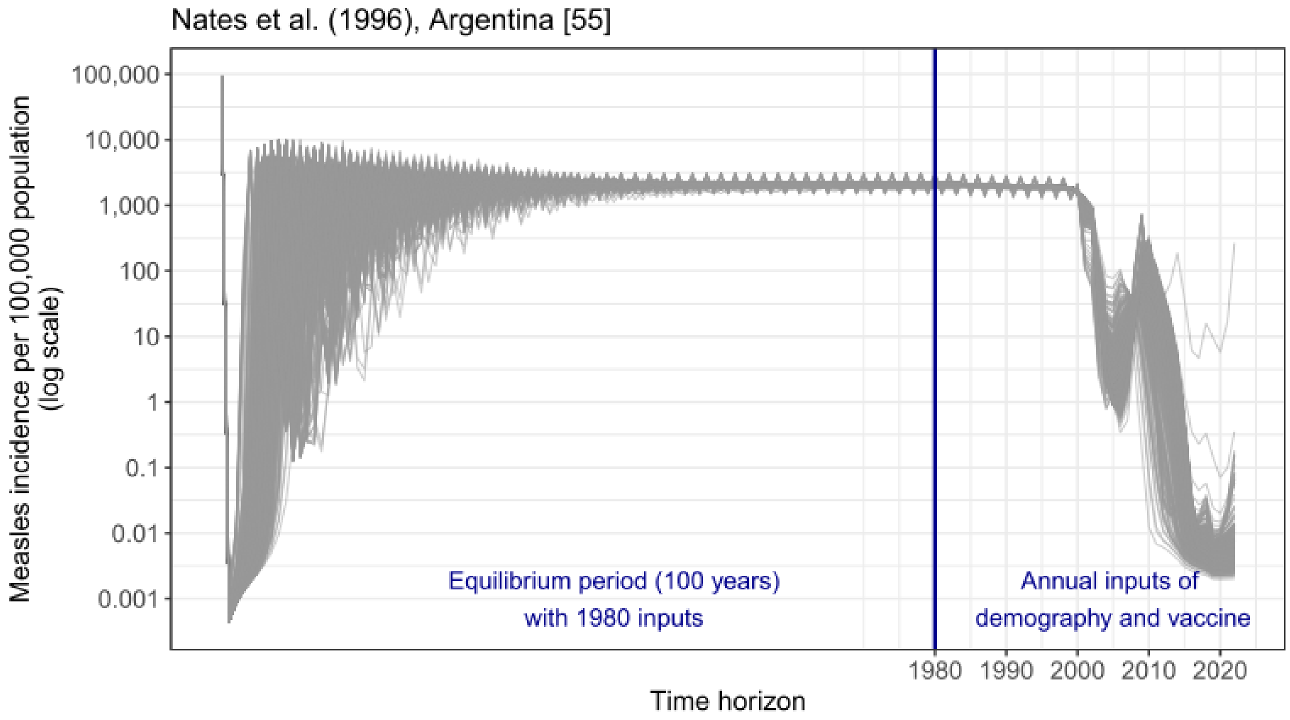

Fig D. Trace plots of posterior log-likelihood and parameters

We obtained trace plots from four MCMC chains for posterior log-likelihood, R_0, duration of maternal immunity, and vaccine effectiveness for the first MCV dose. Studies with the largest sample size in each WHO region based on (A) two- and (B) three-parameter models were selected for presentation.

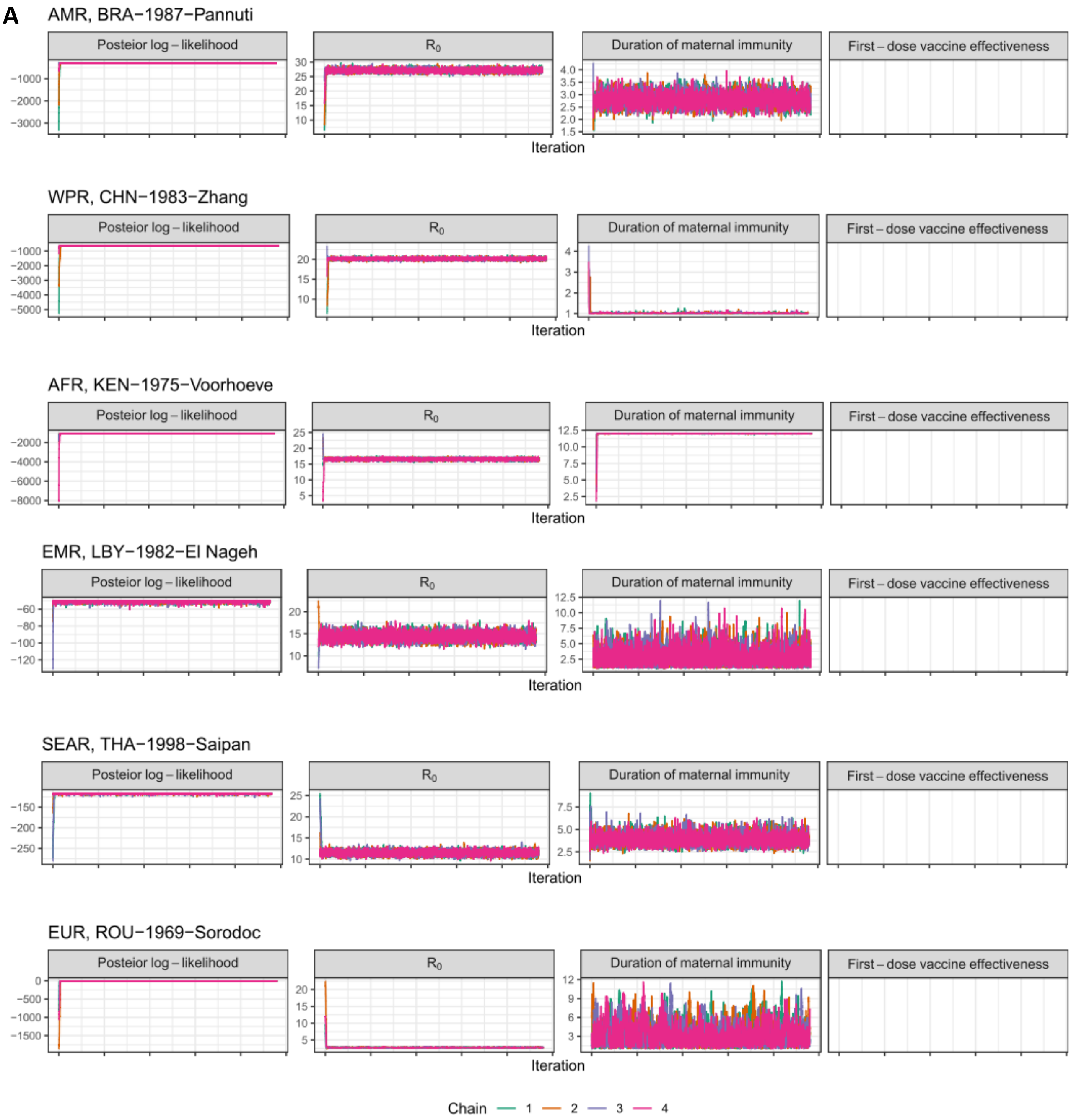

B

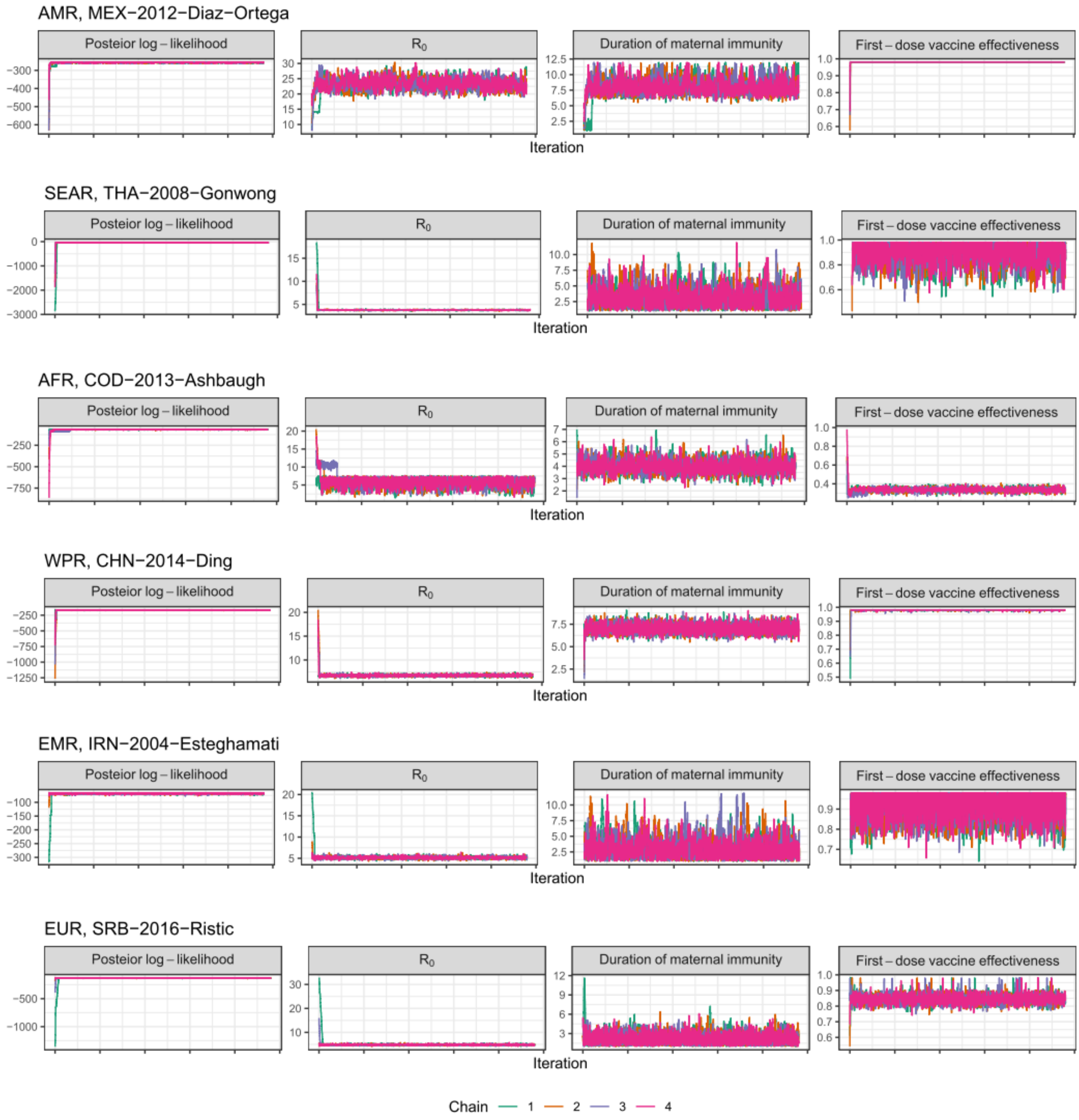
AMR, MEX−2012−Diaz−Ortega
SEAR, THA−2008−Gonwong
AFR, COD−2013−Ashbaugh
WPR, CHN−2014−Ding
EMR, IRN−2004−Esteghamati
EUR, SRB−2016−Ristic
Posteior log – likelihood
R0
Duration of maternal immunity
First – dose vaccine effectiveness
Iteration
Chain 1 2 3 4

Fig E. Prior and posterior parameter distributions

We display prior and posterior density for basic reproduction number (r0), duration of maternal immunity (mat_imm), and vaccine effectiveness for MCV1 (mcv1_ve). Studies with the largest sample size in each WHO region based on (A) two- and (B) three-parameter models were selected for presentation.

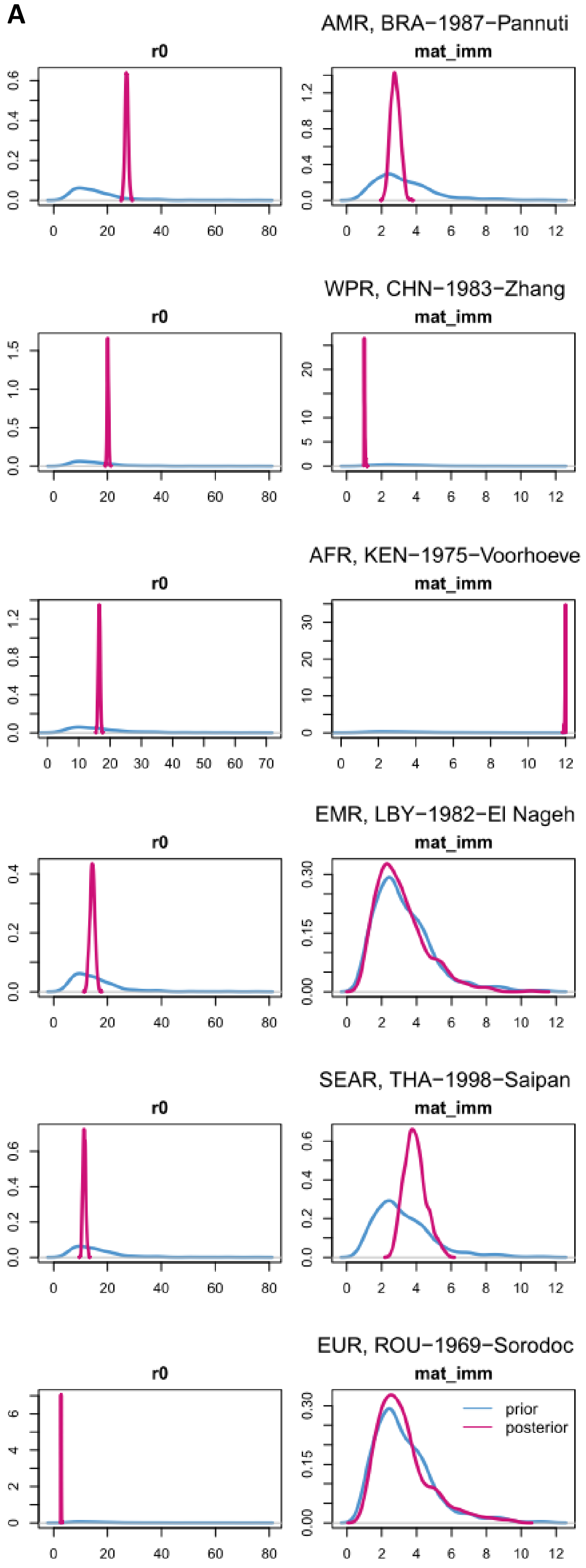

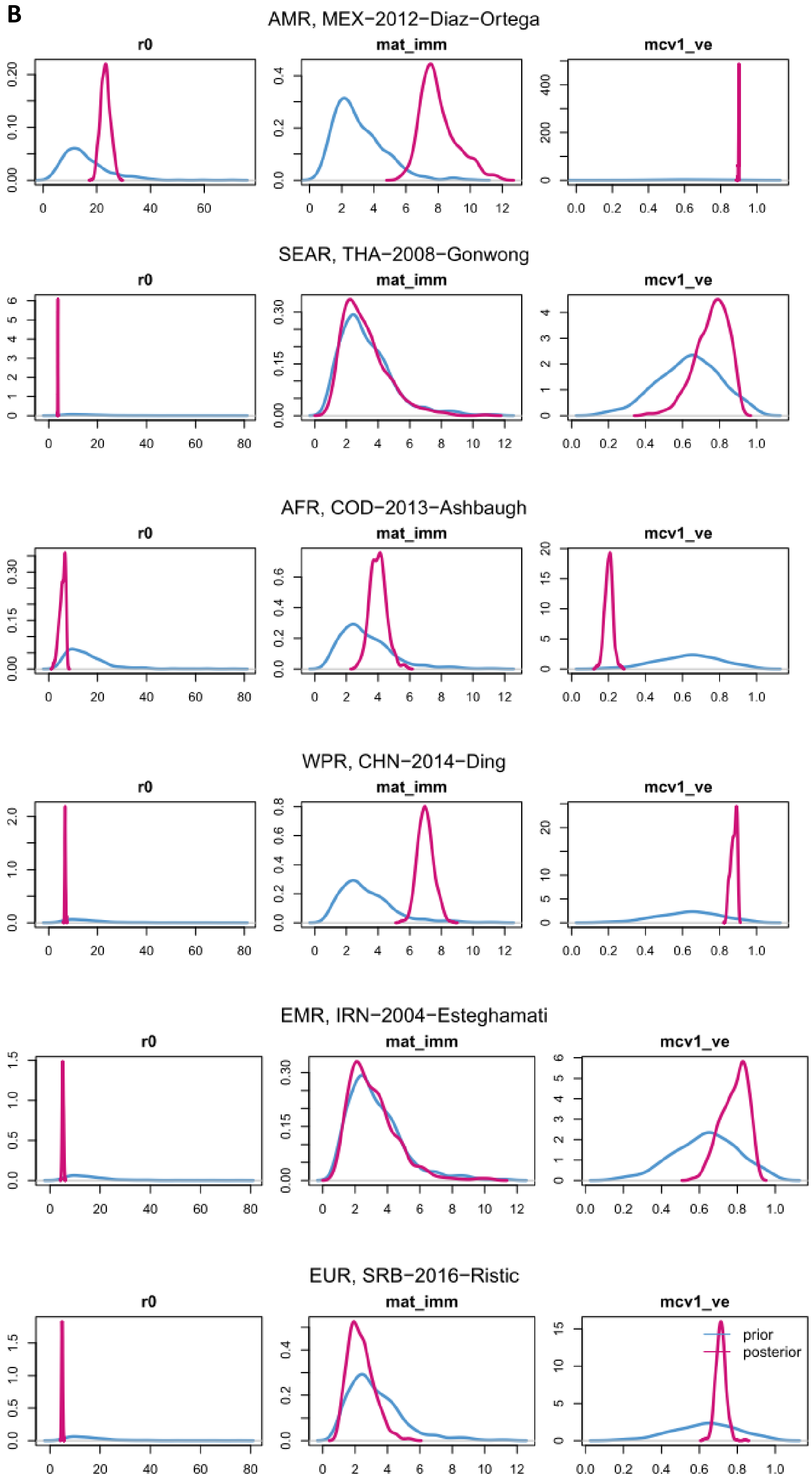
B
AMR, MEX−2012−Diaz−Ortega
SEAR, THA−2008−Gonwong
AFR, COD−2013−Ashbaugh
WPR, CHN−2014−Ding
EMR, IRN−2004−Esteghamati
EUR, SRB−2016−Ristic
r0
mat_imm
mcv1_ve
prior
posterior

Fig F. Pairwise correlation matrix between fitting parameters

We show pairwise correlation plots for basic reproduction number (r0), duration of maternal immunity (mat_imm), and vaccine effectiveness for MCV1 (mcv1_ve). Studies with the largest sample size in each WHO region based on (A) two- and (B) three-parameter models were selected for presentation.

A

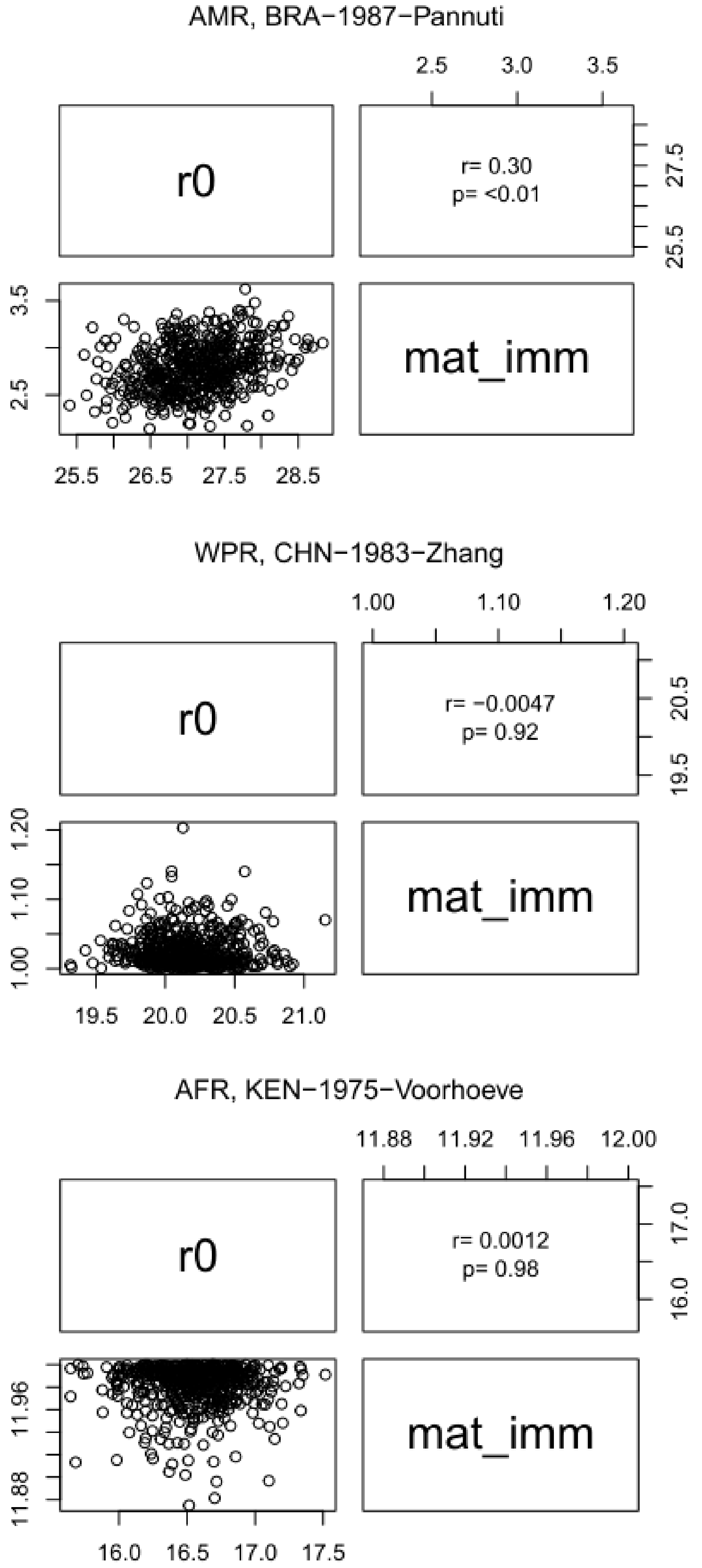

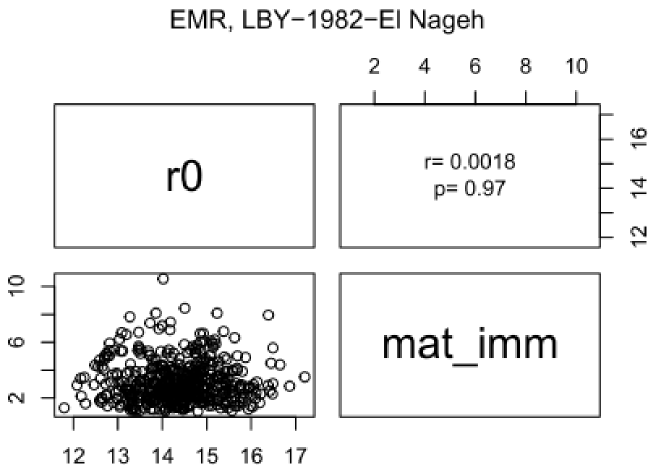
EMR, LBY−1982−El Nageh
r0
r= 0.0018
p= 0.97
mat_imm
2 4 6 8 10
12 14 16
12 13 14 15 16 17

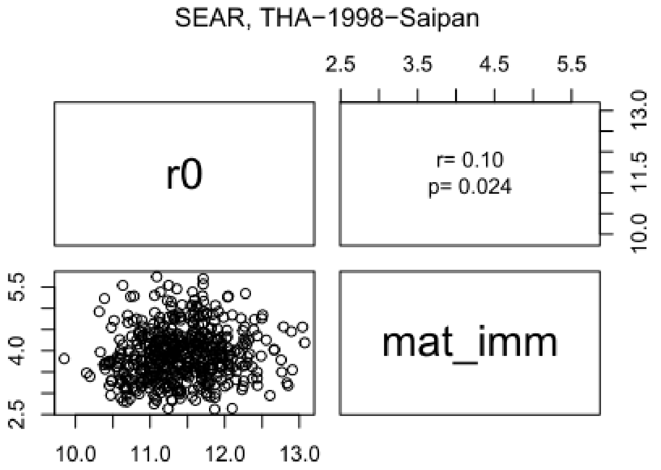
SEAR, THA−1998−Saipan
r0
r= 0.10
p= 0.024
mat_imm
2.5 3.5 4.5 5.5
10.0 11.5 13.0
10.0 11.0 12.0 13.0

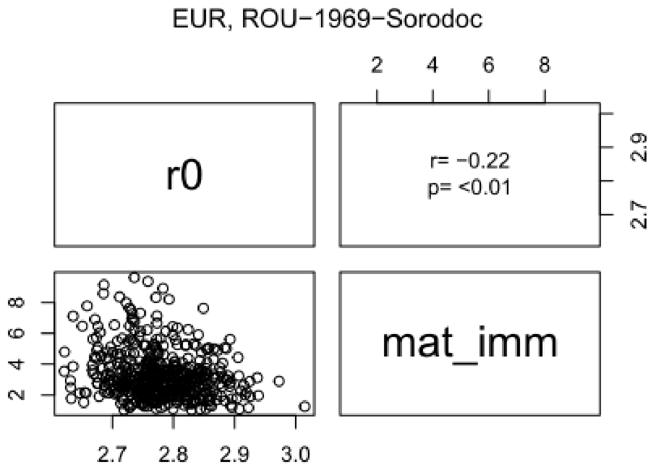
EUR, ROU−1969−Sorodoc
r0
r= −0.22
p= <0.01
mat_imm
2 4 6 8
2.7 2.9
2.7 2.8 2.9 3.0

B

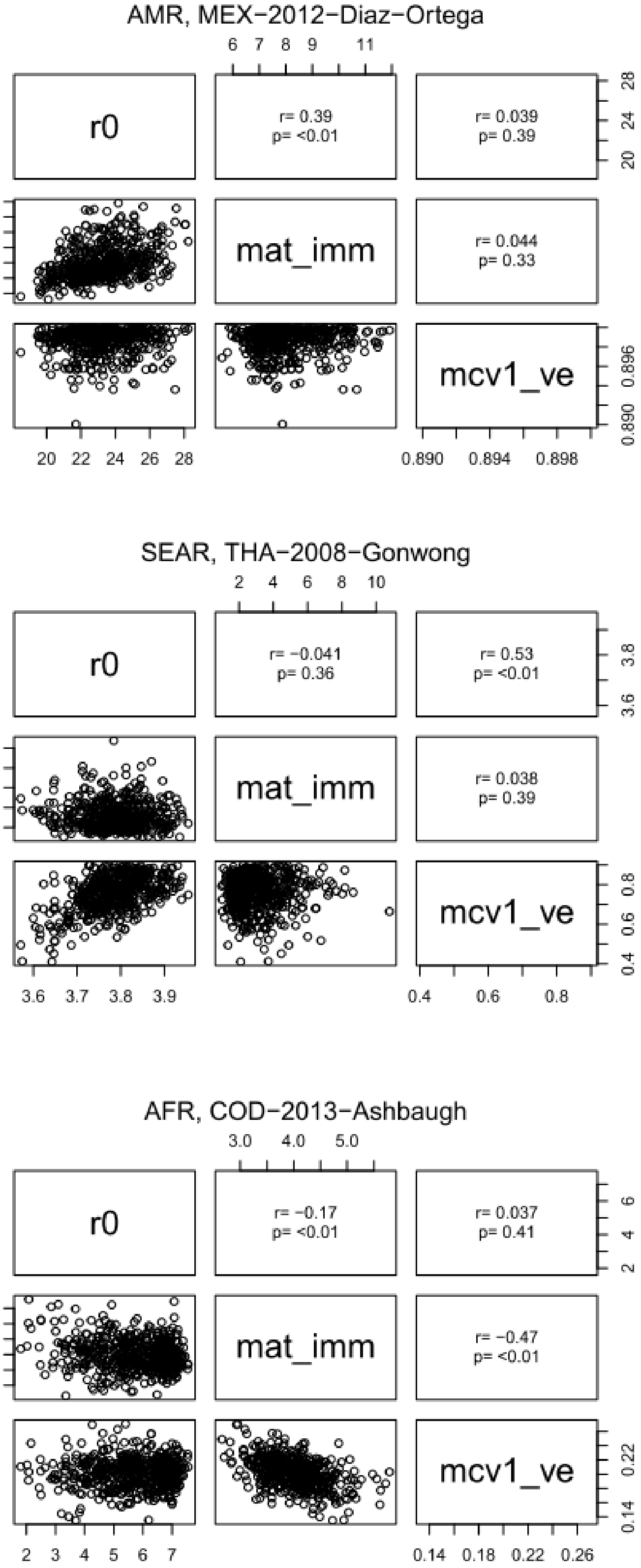
AMR, MEX−2012−Diaz−Ortega
r0
r= 0.39
p= <0.01
r= 0.039
p= 0.39
mat_imm
r= 0.044
p= 0.33
mcv1_ve
SEAR, THA−2008−Gonwong
r0
r= −0.041
p= 0.36
r= 0.53
p= <0.01
mat_imm
r= 0.038
p= 0.39
mcv1_ve
AFR, COD−2013−Ashbaugh
r0
r= −0.17
p= <0.01
r= 0.037
p= 0.41
mat_imm
r= −0.47
p= <0.01
mcv1_ve

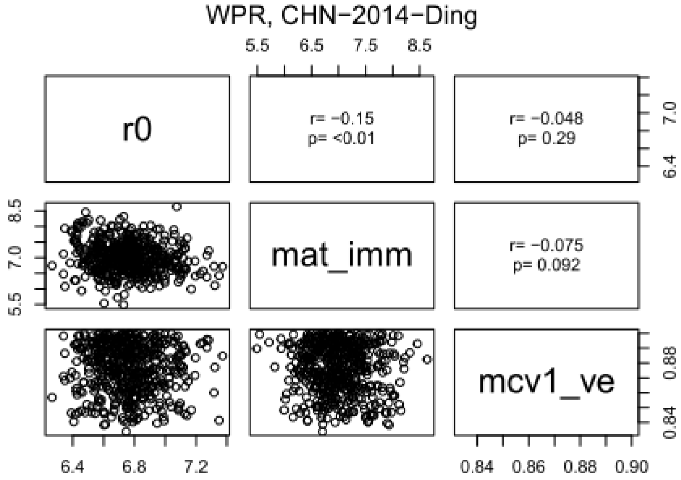
WPR, CHN−2014−Ding
r0
mat_imm
mcv1_ve
r= −0.15
p= <0.01
r= −0.048
p= 0.29
r= −0.075
p= 0.092

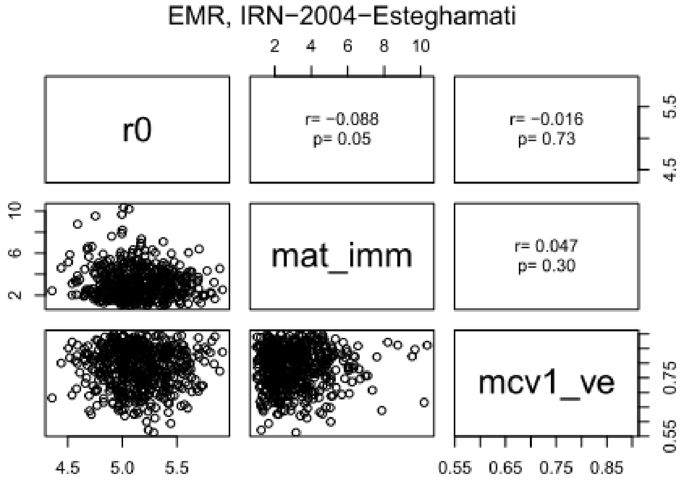
EMR, IRN−2004−Esteghamati
r0
mat_imm
mcv1_ve
r= −0.088
p= 0.05
r= −0.016
p= 0.73
r= 0.047
p= 0.30

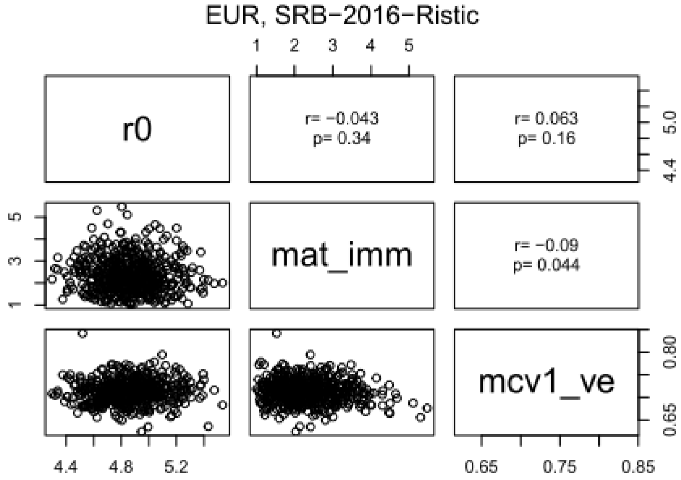
EUR, SRB−2016−Ristic
r0
mat_imm
mcv1_ve
r= −0.043
p= 0.34
r= 0.063
p= 0.16
r= −0.09
p= 0.044

Fig G. Effective sample size and Gelman-Rubin statistic

MCMC diagnostics for R_0, duration of maternal immunity, and vaccine effectiveness for the first measles vaccine dose are presented. Reference lines are set as 200 for (A) effective sample size and 1.1 for (B) Gelman-Rubin statistic.

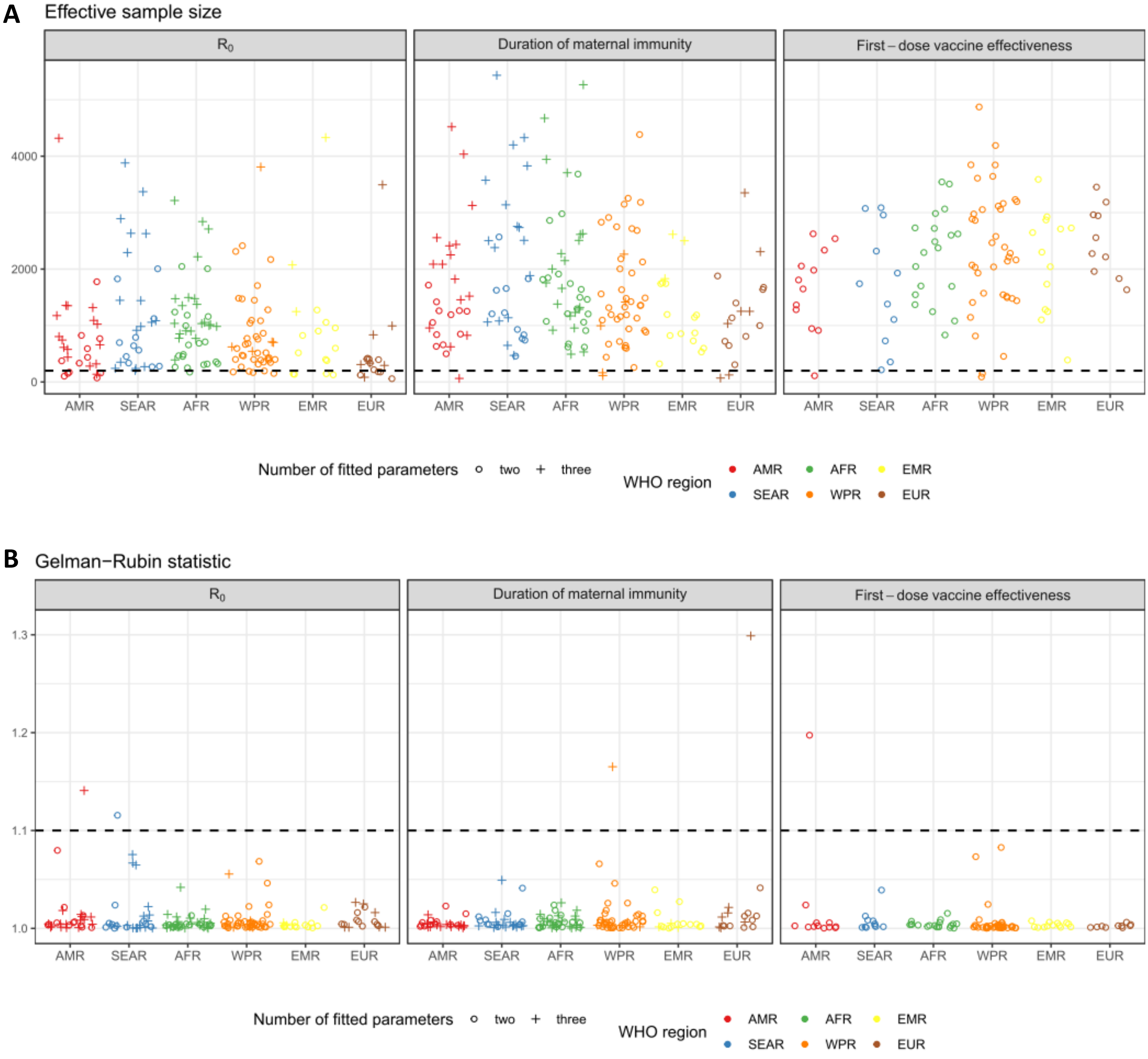

Fig H. Root mean square error of age-specific seroprevalence data

Studies were ranked by the magnitude of root mean square error (RMSE). Darker colour indicates studies conducted in more recent years. The horizontal dashed line indicated 10% of RMSE.

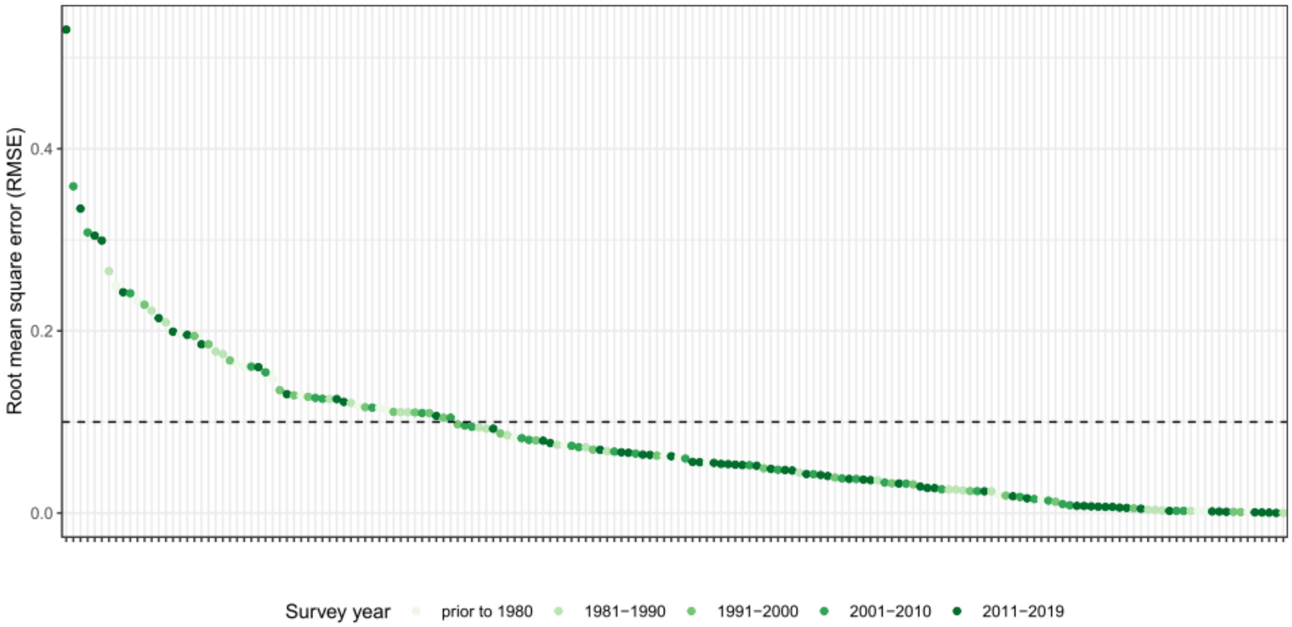

Fig I. Correlations between posterior R_0 and vaccine effectiveness and duration of maternal immunity

(A) displays the correlation between R_0 and vaccine effectiveness among studies conducted in the post-vaccination era (n=103) and (B) shows the correlation only among studies with low or moderate bias (n=60). (C) and (D) show the correlations between duration of maternal immunity among all studies (n=171) and studies with low or moderate bias (n=69), respectively. The Spearman's rank correlation coefficient R and p value are presented. One outlier with median R_0 greater than 100 was removed from this analysis.

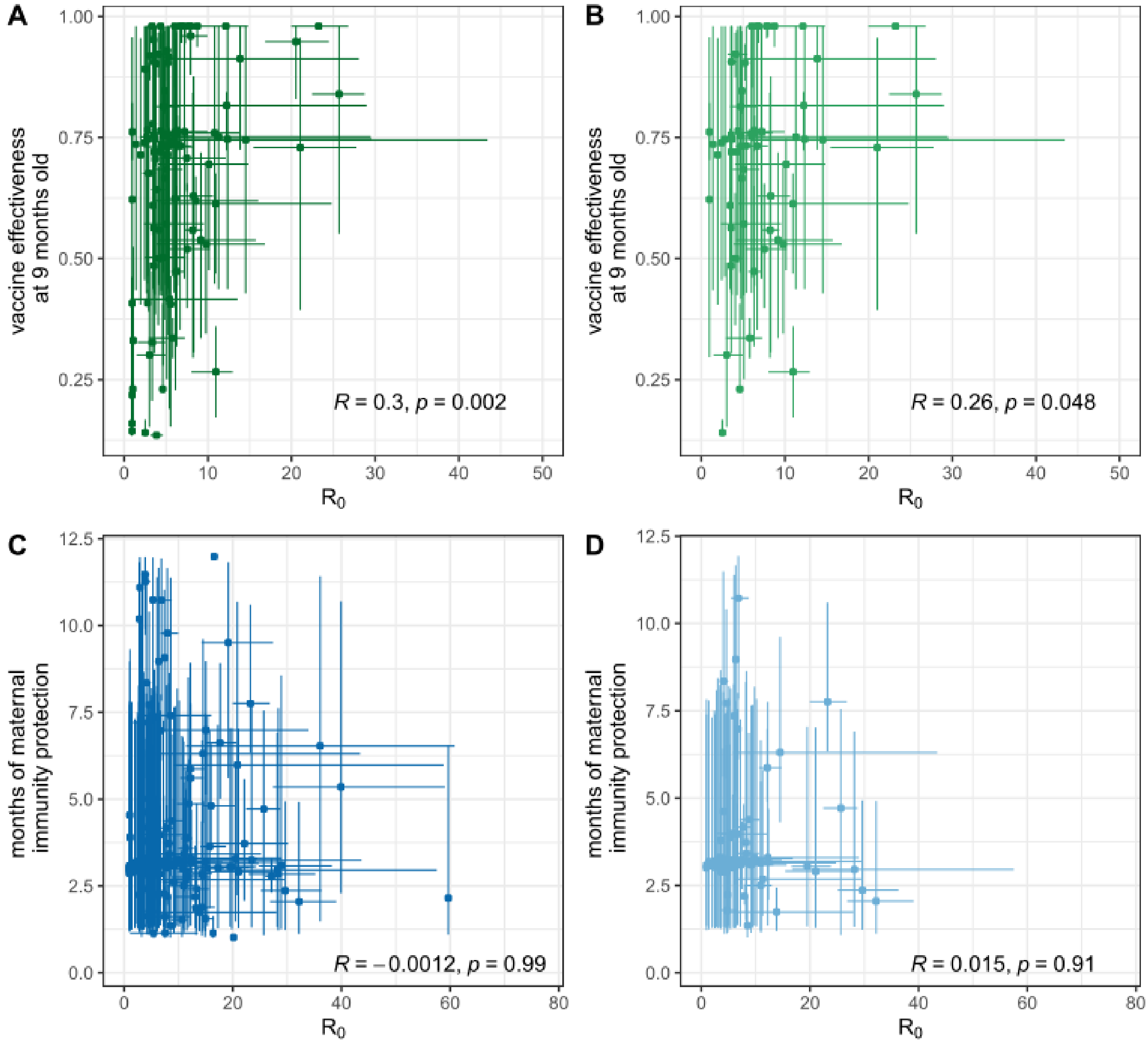

Fig J. Fitted measles R_0 using age-specific seroprevalence data in China, India, Brazil, and Türkiye

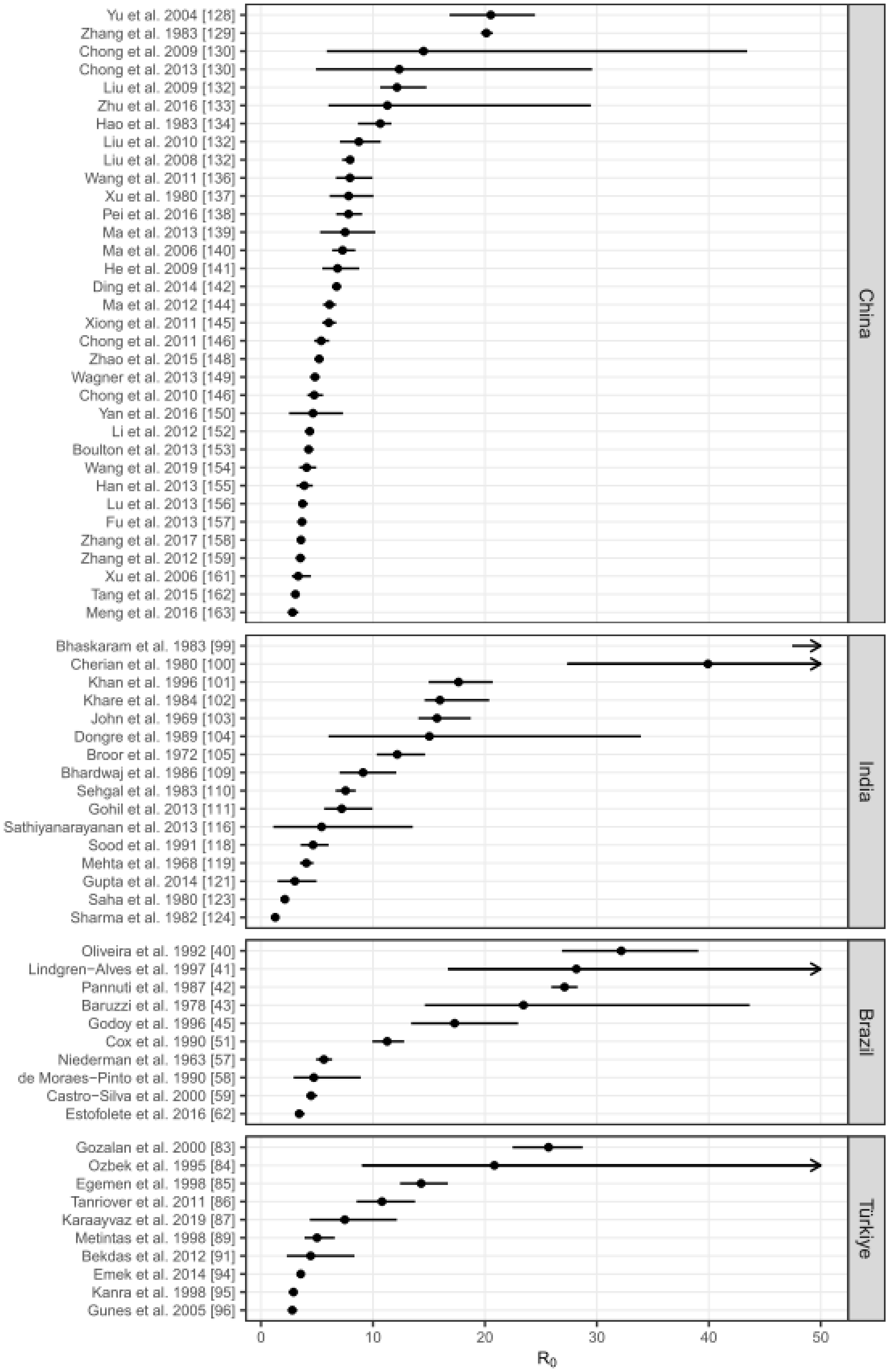

Fig K. Factors associated with pooled R_0 estimates in China, India, Brazil, and Türkiye

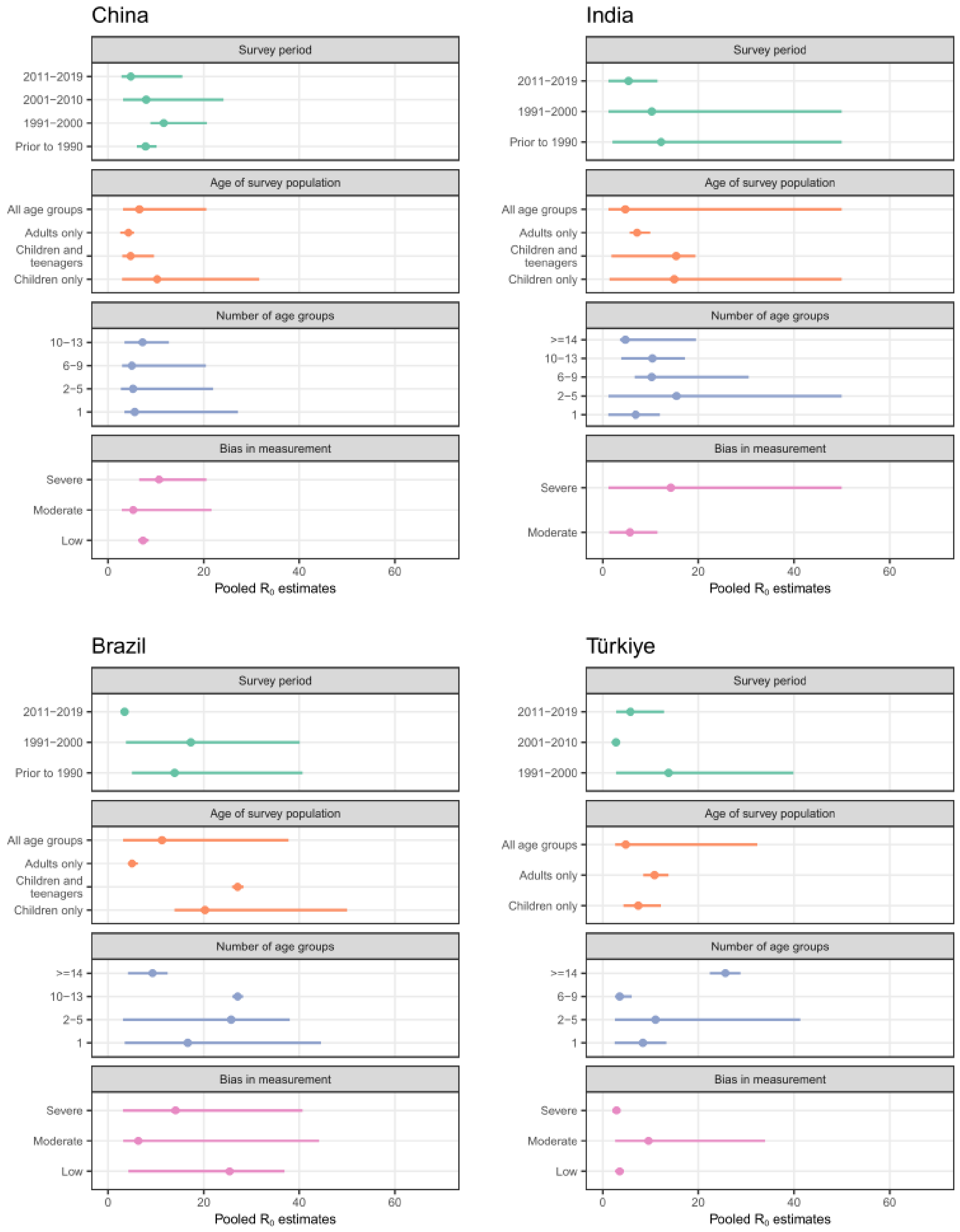

Fig L. Model-estimated and WHO reported measles cases at the year of serosurvey (n=155)

Comparison was based on median estimates and studies were ranked by the scale of difference.

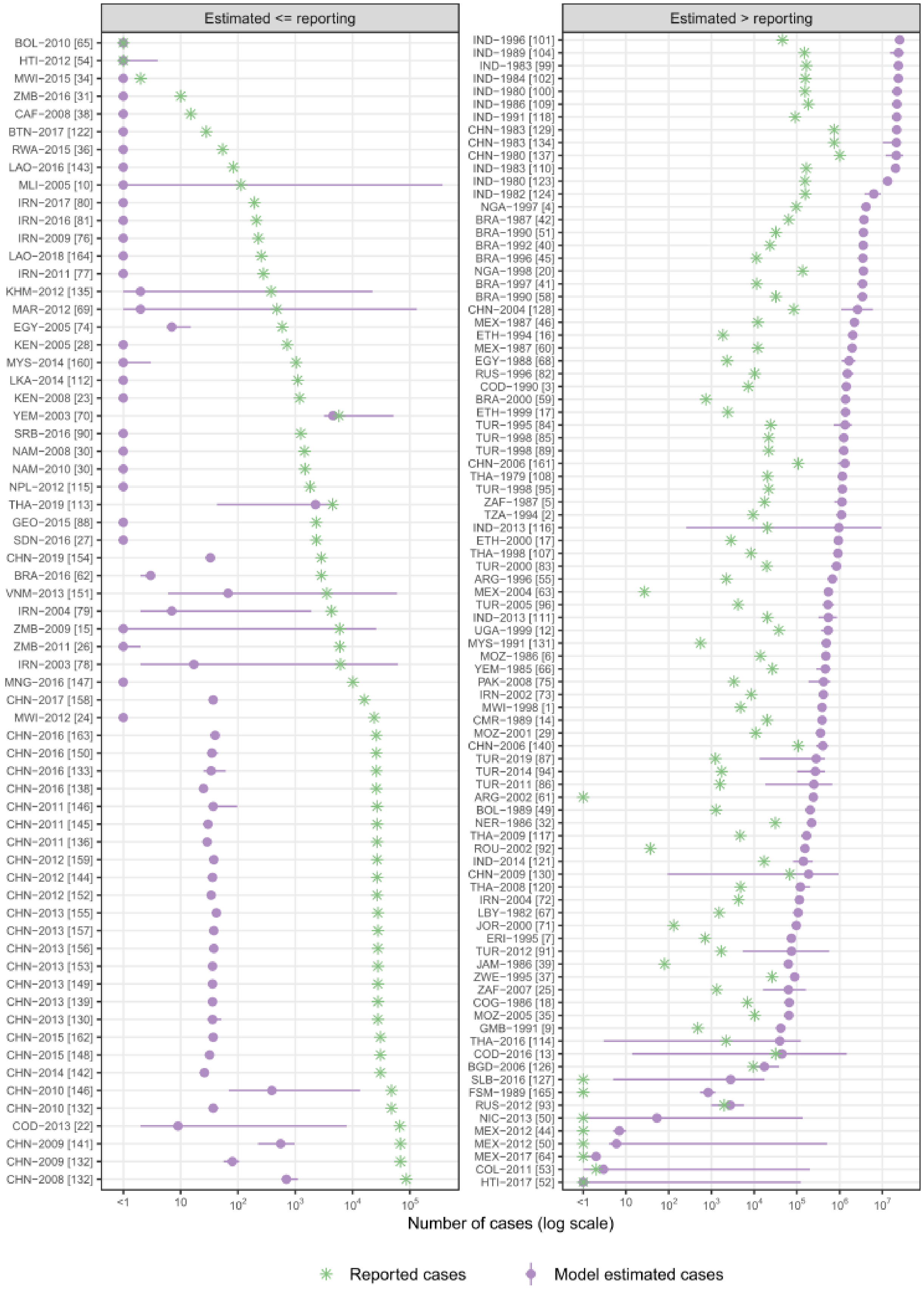

Fig M. Model estimates and Global Burden of Diseases 2021 cases at the year of serosurvey (n=134)

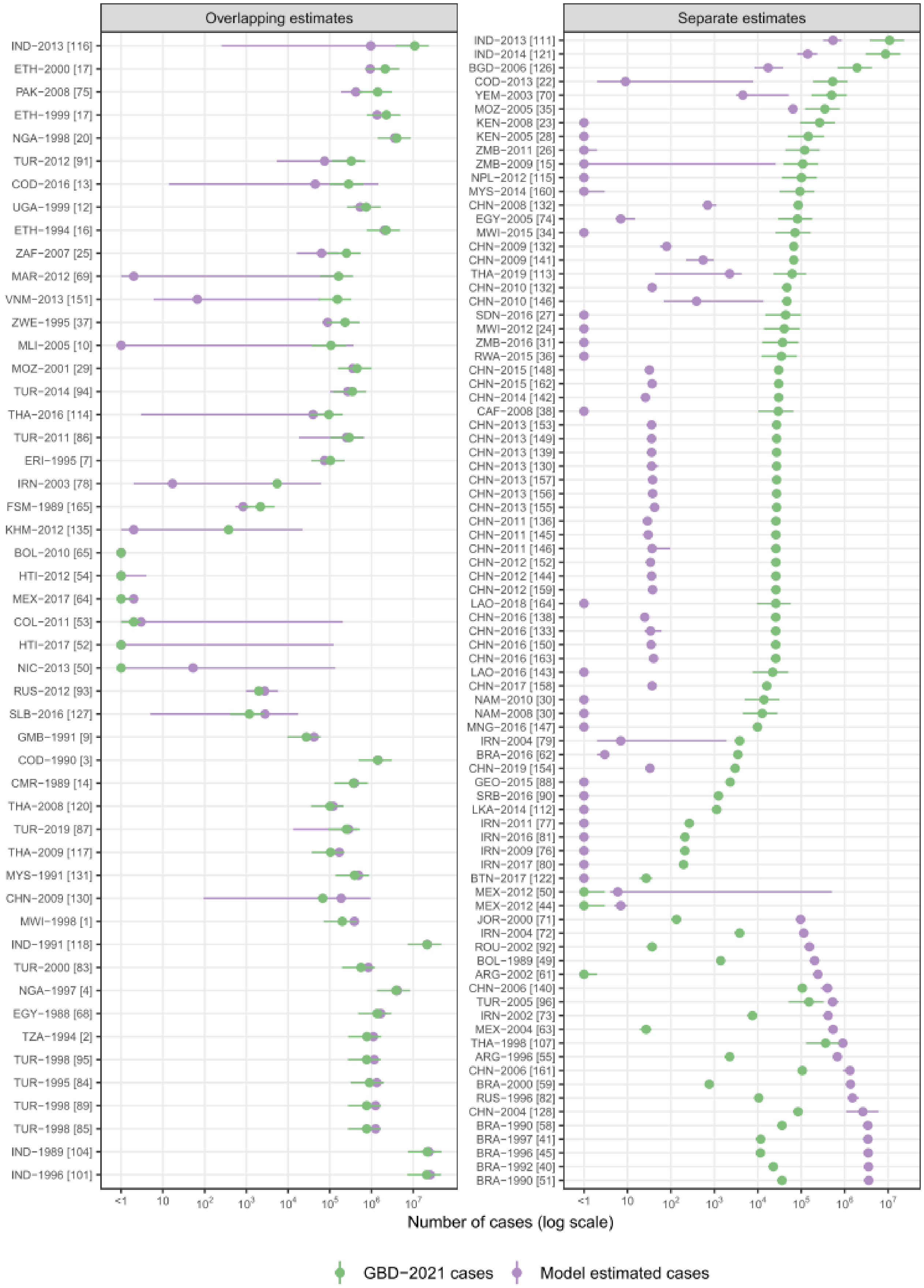

Table B. RSMEs by selected study features

Chi-square test was applied for measuring association between good fit and study features.

Study feature	RMSE >=10% n (%)		RMSE <10% n (%)		p-value
WHO region					0.0702
AFR	12	(6.98)	28	(16.3)	
AMR	7	(4.07)	21	(12.2)	
EMR	7	(4.07)	9	(5.23)	
EUR	5	(2.91)	12	(6.98)	
SEAR	15	(8.72)	13	(7.56)	
WPR	9	(5.23)	34	(19.8)	
Study age group					0.0177
Children only	12	(6.98)	28	(16.3)	
Children and teenagers	12	(6.98)	10	(5.81)	
Adults only	2	(1.16)	19	(11.0)	
All age groups	29	(16.9)	60	(34.9)	
Overall bias					0.0344
Including critical bias	16	(9.30)	20	(11.6)	
Including severe bias	24	(14.0)	42	(24.4)	
Low or moderate bias	15	(8.72)	55	(32.0)	
Survey years					0.0849
Prior to 1980	10	(5.81)	10	(5.81)	
1981—1990	9	(5.23)	17	(9.88)	
1991—2000	12	(6.98)	15	(8.72)	
2001—2010	10	(5.81)	25	(14.5)	
2011—2019	14	(8.14)	50	(29.1)	
Number of age groups					<0.0005
1	1	(0.58)	39	(22.7)	
2—5	25	(14.5)	42	(24.4)	
6—9	17	(9.88)	22	(12.8)	
10—13	7	(4.07)	11	(6.40)	
>=14	5	(2.91)	3	(1.74)	

Fig N. Difference between national and subnational coverage at survey years (n=44)

Based on the location information reported in the original serostudies (Table A in S1 Appendix), we identified the most likely administrative level-1 and/or level-2 location of each serosurvey. We then utilised the corresponding subnational MCV1 coverage from a database recently published by the Institute of Health Metrics and Evaluation (IHME) (https://vizhub.healthdata.org/lsae/vaccines). Only 44 of 103 serostudies (42.7%) conducted in the post-vaccination era were included for analysis due to data availability in both original studies and IHME database.

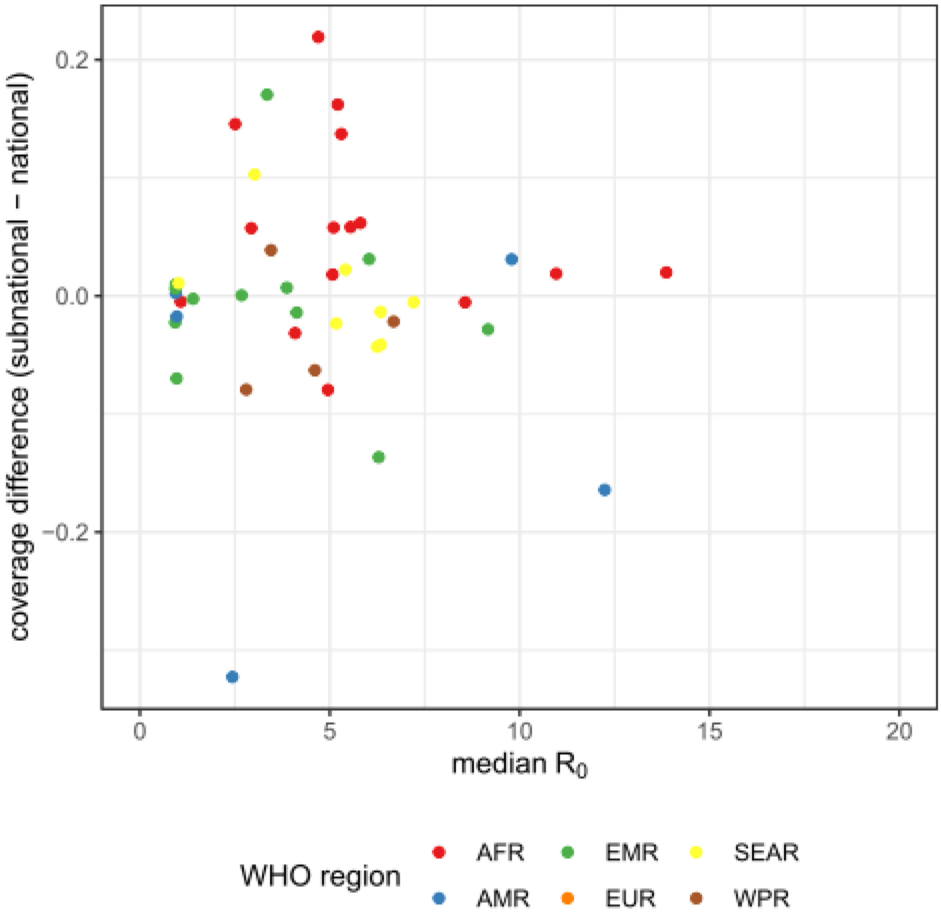

Fig O. Pooled R_0 in serostudies with subnational coverage data

We conducted a sensitivity analysis by removing the serostudies with >10% absolute difference between national and subnational coverage levels.

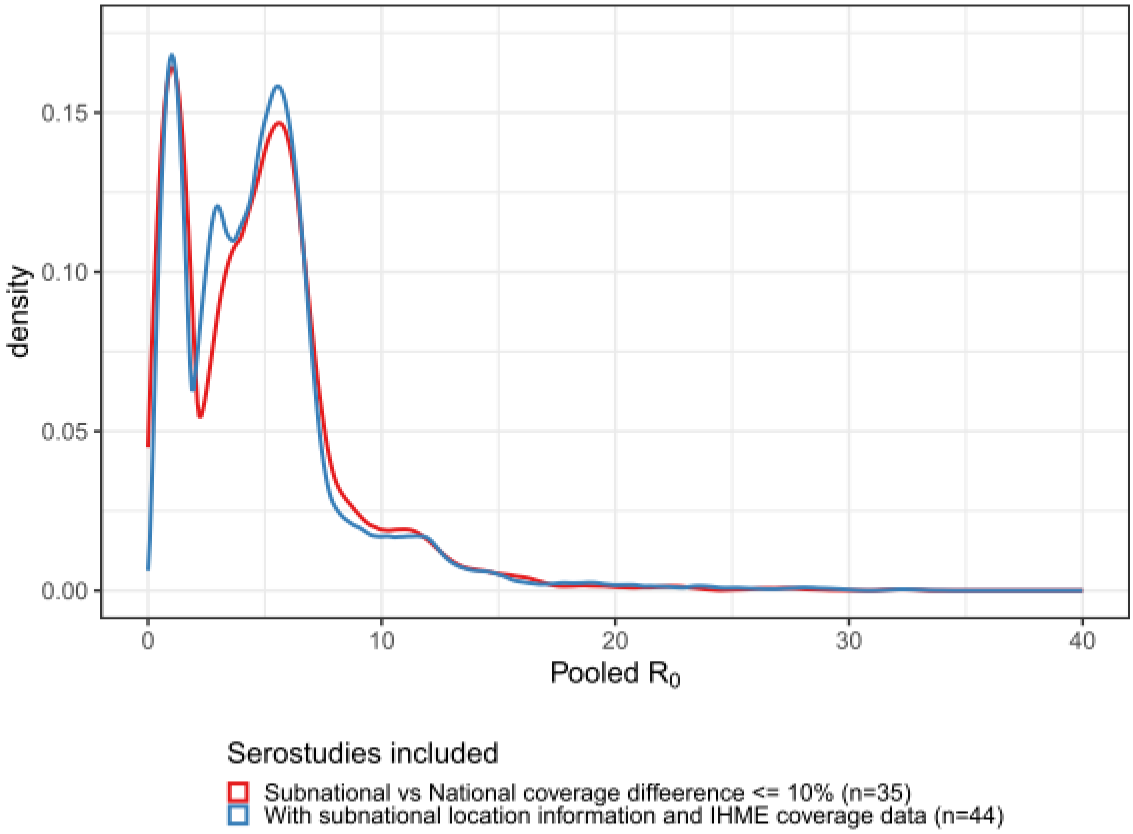

References

1. Takechi M, Matsuo M, Butao D, Zungu IL, Chakanika I, Michongwe J. Measles sero-surveillance during mass immunisation campaign in Malawi. East Afr Med J. 2001;78(1):4-8.
2. Lyamuya EF, Matee MI, Aaby P, Scheutz F. Serum levels of measles IgG antibody activity in children under 5 years in Dar-es-Salaam, Tanzania. Ann Trop Paediatr. 1999;19(2):175-83.
3. Cutts FT, Othepa O, Vernon AA, Nyandu B, Markowitz LE, Deforest A, et al. Measles control in Kinshasa, Zaire improved with high coverage and use of medium titre Edmonston Zagreb vaccine at age 6 months. Int J Epidemiol. 1994;23(3):624-31.
4. Oyefolu AO, Omilabu SA. Measles HI-antibody levels in Lagos children, Nigeria: a follow-up study to resurgence of measles in Lagos metropolis. West Afr J Med. 2001;20(3):238-42.
5. De Swardt R, Ijsselmuiden CB, Johnson S. Vaccination status and seroprevalence of measles and polio antibodies in 1-6-year-old children in the Elim health ward of Gazankulu. S Afr Med J. 1990;78(12):726-8.
6. Cutts FT, Smith PG, Colombo S, Mann G, Ascherio A, Soares AC. Field evaluation of measles vaccine efficacy in Mozambique. Am J Epidemiol. 1990;131(2):349-55.
7. Tolfvenstam T, Enbom M, Ghebrekidan H, Rudén U, Linde A, Grandien M, et al. Seroprevalence of viral childhood infections in Eritrea. J Clin Virol. 2000;16(1):49-54.
8. Voorhoeve AM, Muller AS, Schulpen TW, Gemert W, Valkenburg HA, Ensering HE. Agents affecting health of mother and child in a rural area of Kenya. III. The epidemiology of measles. Trop Geogr Med. 1977;29(4):428-40.
9. Fortuin M, Maine N, Mendy M, Hall A, George M, Whittle H. Measles, polio and tetanus toxoid antibody levels in Gambian children aged 3 to 4 years following routine vaccination. Transactions of the Royal Society of Tropical Medicine and Hygiene. 1995;89(3):326-9.
10. Tapia MD, Sow SO, Medina-Moreno S, Lim Y, Pasetti MF, Kotloff K, et al. A serosurvey to identify the window of vulnerability to wild-type measles among infants in rural Mali. Am J Trop Med Hyg. 2005;73(1):26-31.
11. Munube GM. Measles sero-immunity in rural non-vaccinated children of Busoga District, Uganda. East Afr Med J. 1979;56(7):335-8.
12. Waibale P, Bowlin SJ, Mortimer EA, Jr., Whalen C. The effect of human immunodeficiency virus-1 infection and stunting on measles immunoglobulin-G levels in children vaccinated against measles in Uganda. Int J Epidemiol. 1999;28(2):341-6.
13. Keating P, Carrion Martin AI, Blake A, Lechevalier P, Uzzeni F, Gignoux E, et al. Measles seroprevalence after reactive vaccination campaigns during the 2015 measles outbreak in four health zones of the former Katanga Province, Democratic Republic of Congo. BMC Public Health. 2019;19(1):1153.
14. Ndumbe PM, Atchou G, Biwole M, Lobe V, Ayuk-Takem J. Infections among pygmies in the Eastern Province of Cameroon. Med Microbiol Immunol. 1993;182(6):281-4.
15. Lowther SA, Curriero FC, Kalish BT, Shields TM, Monze M, Moss WJ. Population immunity to measles virus and the effect of HIV-1 infection after a mass measles vaccination campaign in Lusaka, Zambia: a cross-sectional survey. Lancet. 2009;373(9668):1025-32.
16. Enquselassie F, Ayele W, Dejene A, Messele T, Abebe A, Cutts FT, et al. Seroepidemiology of measles in Addis Ababa, Ethiopia: implications for control through vaccination. Epidemiol Infect. 2003;130(3):507-19.
17. Nigatu W, Samuel D, Cohen B, Cumberland P, Lemma E, Brown DW, et al. Evaluation of a measles vaccine campaign in Ethiopia using oral-fluid antibody surveys. Vaccine. 2008;26(37):4769-74.
18. Dabis F, Waldman RJ, Mann GF, Commenges D, Madzou G, Jones TS. Loss of maternal measles antibody during infancy in an African city. Int J Epidemiol. 1989;18(1):264-8.
19. Ogunmekan DA, Bracken P, Marshall WC. A seroepidemiological study of measles infection in normal and handicapped persons in Lagos, Nigeria. J Trop Med Hyg. 1981;84(4):175-8.
20. Hartter HK, Oyedele OI, Dietz K, Kreis S, Hoffman JP, Muller CP. Placental transfer and decay of maternally acquired antimeasles antibodies in Nigerian children. The Pediatric infectious disease journal. 2000;19(7):635-41.
21. Omer AH, McLaren ML, Johnson BK, Chanas AC, Brumpt I, Gardner P, et al. A seroepidemiological survey in the Gezira, Sudan, with special reference to arboviruses. J Trop Med Hyg. 1981;84(2):63-6.

22. Ashbaugh HR, Cherry JD, Hoff NA, Doshi RH, Alfonso VH, Gadoth A, et al. Measles antibody levels among vaccinated and unvaccinated children 6-59 months of age in the Democratic Republic of the Congo, 2013-2014. Vaccine. 2020;38(9):2258-65.
23. Merkel M, Ben-Youssef L, Newman LP, Gitome V, Gataguta A, Lohman-Payne B, et al. Seroprevalence of measles IgG among HIV-1-infected and uninfected Kenyan adults. Int J Infect Dis. 2014;19:103-5.
24. Polonsky JA, Singh B, Masiku C, Langendorf C, Kagoli M, Hurtado N, et al. Exploring HIV infection and susceptibility to measles among older children and adults in Malawi: a facility-based study. Int J Infect Dis. 2015;31:61-7.
25. Jallow S, Cutland CL, Masbou AK, Adrian P, Madhi SA. Maternal HIV infection associated with reduced transplacental transfer of measles antibodies and increased susceptibility to disease. J Clin Virol. 2017;94:50-6.
26. Sutcliffe CG, Searle K, Matakala HK, Greenman MP, Rainwater-Lovett K, Thuma PE, et al. Measles and Rubella Seroprevalence Among HIV-infected and Uninfected Zambian Youth. The Pediatric infectious disease journal. 2017;36(3):301-6.
27. Adam O, Musa A, Kamer A, Sausy A, Tisserand E, Hübschen JM. Seroprevalence of measles, mumps, and rubella and genetic characterization of mumps virus in Khartoum, Sudan. Int J Infect Dis. 2020;91:87-93.
28. Scott S, Cumberland P, Shulman CE, Cousens S, Cohen BJ, Brown DW, et al. Neonatal measles immunity in rural Kenya: the influence of HIV and placental malaria infections on placental transfer of antibodies and levels of antibody in maternal and cord serum samples. J Infect Dis. 2005;191(11):1854-60.
29. Mandomando IM, Naniche D, Pasetti MF, Vallès X, Cuberos L, Nhacolo A, et al. Measles-specific neutralizing antibodies in rural Mozambique: seroprevalence and presence in breast milk. Am J Trop Med Hyg. 2008;79(5):787-92.
30. Cardemil CV, Jonas A, Beukes A, Anderson R, Rota PA, Bankamp B, et al. Measles immunity among pregnant women aged 15-44 years in Namibia, 2008 and 2010. Int J Infect Dis. 2016;49:189-95.
31. Hayford K, Mutembo S, Carcelen A, Matakala HK, Munachoonga P, Winter A, et al. Measles and rubella serosurvey identifies rubella immunity gap in young adults of childbearing age in Zambia: The added value of nesting a serological survey within a post-campaign coverage evaluation survey. Vaccine. 2019;37(17):2387-93.
32. Loutan L, Paillard S. Measles in a west African nomadic community. Bulletin of the World Health Organization. 1992;70(6):741-4.
33. Breman JG, Coffi E, Bomba-Ire R, Foster SO, Herrmann KL. Evaluation of a measles-smallpox vaccination campaign by a sero-epidemiologic method. Am J Epidemiol. 1975;102(6):564-71.
34. Polonsky JA, Juan-Giner A, Hurtado N, Masiku C, Kagoli M, Grais RF. Measles seroprevalence in Chiradzulu district, Malawi: Implications for evaluating vaccine coverage. Vaccine. 2015;33(36):4554-8.
35. Jani JV, Holm-Hansen C, Mussá T, Zango A, Manhiça I, Bjune G, et al. Assessment of measles immunity among infants in Maputo City, Mozambique. BMC Public Health. 2008;8:386.
36. Seruyange E, Gahutu JB, Mambo Muvunyi C, Uwimana ZG, Gatera M, Twagirumugabe T, et al. Measles seroprevalence, outbreaks, and vaccine coverage in Rwanda. Infect Dis (Lond). 2016;48(11-12):800-7.
37. Obi CL, Tswana SA, Moyo SR, Berejena C. Measles virus haemagglutination-inhibition antibodies among pregnant and non-pregnant women in the vaccine era in Harare, Zimbabwe. Cent Afr J Med. 1996;42(5):135-8.
38. Manirakiza A, Kipela JM, Sosler S, Daba RM, Gouandjika-Vasilache I. Seroprevalence of measles and natural rubella antibodies among children in Bangui, Central African Republic. BMC Public Health. 2011;11:327.
39. Christie CD, Lee-Hirsh J, Rogall B, Merrill S, Ramlal AA, Karian V, et al. Durability of passive measles antibody in Jamaican children. Int J Epidemiol. 1990;19(3):698-702.
40. Oliveira SA, Siqueira MM, Mann GF, Costa AJ, Almeida MT, Stavola MS, et al. Measles antibody prevalence after mass immunization campaign in Niterói, state of Rio de Janeiro, Brazil. Rev Inst Med Trop Sao Paulo. 1996;38(5):355-8.
41. Lindgren-Alves CR, Freire LM, Oliveira RC, Guerra HL, Da-Silva EE, Siqueira MM, et al. [Search of antimeasles antibodies in HIV-infected children after basic immunization]. J Pediatr (Rio J). 2001;77(6):496-

502.
42. Pannuti CS, Moraes JC, Souza VA, Camargo MC, Hidalgo NT. Measles antibody prevalence after mass immunization in São Paulo, Brazil. Bulletin of the World Health Organization. 1991;69(5):557-60.
43. Baruzzi RG, Abdala N, Black FL. Measles and measles vaccination in isolated Amerindian tribes. II. The 1978/79 Xingu epidemic. Trop Geogr Med. 1982;34(1):7-12.
44. Díaz-Ortega JL, Ferreira-Guerrero E, Cruz-Hervert LP, Delgado-Sánchez G, Ferreyra-Reyes L, Yanes-Lane M, et al. Seroprevalence of measles antibodies and factors associated with susceptibility: a national survey in Mexico using a plaque reduction neutralization test. Sci Rep. 2020;10(1):17488.
45. Godoy I, Meira DA. [Seroprevalence of measles antibodies in a pediatric population from Botucatu, São Paulo State, Brazil]. Rev Soc Bras Med Trop. 2000;33(3):259-64.
46. Sepúlveda J, Tapia-Conyer R, Valdespino JL, Quiroz G, Salvatierra B, Zárate ML, et al. [Seroepidemiology of measles in Mexico]. Salud Publica Mex. 1992;34(2):148-56.
47. Ruíz-Gómez J, Bustamante Calvillo ME. [Seroepidemiology of chickenpox, measles and parotitis in the Republic of Mexico. III. Chickenpox]. Salud Publica Mex. 1978;20(1):19-27.
48. Gutiérrez G, Ruiz-Gómez J. [Seroepidemiology of 10 infectious diseases in children in Mexico City. Measles, rubella, parotitis, typhoid, whooping cough, amebiasis, influenza and adenovirus infections, Mycoplasma pneumoniae and herpes simplex]. Gac Med Mex. 1973;105(6):529-40.
49. Guglielmetti P, Bianchi Bandinelli ML, Bartoloni A, Gamboa H, Roselli M, Valensin PE. Prevalence of measles antibodies before and after vaccination in previously unvaccinated children of the Cordillera Province (Santa Cruz Department, Bolivia). J Trop Med Hyg. 1994;97(4):231-5.
50. Colson KE, Zúñiga-Brenes P, Ríos-Zertuche D, Conde-Glez CJ, Gagnier MC, Palmisano E, et al. Comparative Estimates of Crude and Effective Coverage of Measles Immunization in Low-Resource Settings: Findings from Salud Mesoamérica 2015. PloS one. 2015;10(7):e0130697.
51. Cox MJ, Azevedo RS, Massad E, Fooks AR, Nokes DJ. Measles antibody levels in a vaccinated population in Brazil. Transactions of the Royal Society of Tropical Medicine and Hygiene. 1998;92(2):227-30.
52. Minta AA, Andre-Alboth J, Childs L, Nace D, Rey-Benito G, Boncy J, et al. Seroprevalence of Measles, Rubella, Tetanus, and Diphtheria Antibodies among Children in Haiti, 2017. Am J Trop Med Hyg. 2020;103(4):1717-25.
53. González MM, Sarmiento L, Giraldo AM, Padilla L, Rey-Benito G, Castaño JC. [Seroprevalence of antibodies to measles, rubella, mumps, hepatitis B viruses and all three poliovirus serotypes among children in Quindío, Colombia]. Rev Salud Publica (Bogota). 2016;18(1):95-103.
54. Fitter DL, Anselme R, Paluku G, Rey G, Flannery B, Tohme RA, et al. Seroprevalence of measles and rubella antibodies in pregnant women Haiti, 2012. Vaccine. 2013;32(1):69-73.
55. Nates SV, Giordano MO, Medeot SI, Martínez LC, Baudagna AM, Naretto E, et al. Loss maternally derived measles immunity in Argentinian infants. The Pediatric infectious disease journal. 1998;17(4):313-6.
56. Golubjatnikov R, Leppla L, Filloy L. [Hemagglutination inhibiting antibodies to measles, rubella, and parotitis. Study of their prevalence in a community of the Mexican plateau]. Salud Publica Mex. 1970;12(5):603-9.
57. Niederman JC, Henderson JR, Opton EM, Black FL, Skvrnova K. A nationwide serum survey of Brazilian military recruits, 1964. II. Antibody patterns with arboviruses, polioviruses, measles and mumps. Am J Epidemiol. 1967;86(2):319-29.
58. de Moraes-Pinto MI, Farhat CK, Carbonare SB, Curti SP, Otsubo ME, Lazarotti DS, et al. Maternally acquired immunity in newborns from women infected by the human immunodeficiency virus. Acta Paediatr. 1993;82(12):1034-8.
59. Castro-Silva R, Camacho LA, Amorim L, Medeiros AD, Ferreira DA, Oliveira SA, et al. Serological surveillance of measles in blood donors in Rio de Janeiro, Brazil. Rev Panam Salud Publica. 2003;14(5):334-40.
60. Fajardo-Gutiérrez A, Yañez-Velasco LB, López-Cervantes M, Yamamoto-Kimura L. [Susceptibility to measles in the adolescent population of the Federal District]. Bol Med Hosp Infant Mex. 1990;47(9):636-44.
61. Dayan GH, Panero MS, Urquiza A, Molina M, Prieto S, Del Carmen Perego M, et al. Rubella and measles seroprevalence among women of childbearing age, Argentina, 2002. Epidemiol Infect. 2005;133(5):861-9.

62. Estofolete CF, Milhim B, França CCG, Silva G, Augusto MT, Terzian ACB, et al. Prevalence of Measles Antibodies in São José do Rio Preto, São Paulo, Brazil: A serological survey model. Sci Rep. 2020;10(1):5179.
63. Belaunzarán-Zamudio PF, García-León ML, Wong-Chew RM, Villasís-Keever A, Cuellar-Rodríguez J, Mosqueda-Gómez JL, et al. Early loss of measles antibodies after MMR vaccine among HIV-infected adults receiving HAART. Vaccine. 2009;27(50):7059-64.
64. Sánchez-Alemán MA, Gutiérrez-Pérez IA, Díaz-Salgado N, Zaragoza-García O, Olamendi-Portugal M, Castro-Alarcón N, et al. Low Seroprevalence of Measles-Specific IgG in Children of Three Ethnic Groups from Mexico: Influence of Age, Sex, Malnutrition and Family Size. Vaccines (Basel). 2021;9(3).
65. Masuet-Aumatell C, Ramon-Torrell JM, Casanova-Rituerto A, Banqué Navarro M, Dávalos Gamboa Mdel R, Montaño Rodríguez SL. Measles in Bolivia: A 'honeymoon period'. Vaccine. 2013;31(16):2097-102.
66. Strauss J, Dobahi SS, Danes L, Kopecký K, Svandová E. Serological survey of measles in Yemen in 1985. J Hyg Epidemiol Microbiol Immunol. 1989;33(1):71-4.
67. el Nageh MM, Khadre MA. Prevalence of antibodies against measles virus in Tripoli, The Socialist People's Libyan Arab Jamahiriya. Transactions of the Royal Society of Tropical Medicine and Hygiene. 1989;83(6):848-50.
68. Fathy MM, el-Khashaab TH, Darwish MA. Antibody level after measles vaccination. J Egypt Public Health Assoc. 1992;67(3-4):369-78.
69. Haban H, Benchekroun S, Sadeq M, Tajounte L, Ahmed HJ, Benjouad A, et al. Seroprevalence of measles vaccine antibody response in vertically HIV-infected children, in Morocco. BMC Infect Dis. 2018;18(1):680.
70. Sallam TA, Al-Jaufy AY, Al-Shaibany KS, Ghauth AB, Best JM. Prevalence of antibodies to measles and rubella in Sana'a, Yemen. Vaccine. 2006;24(37-39):6304-8.
71. Bdour S, Batayneh N. Present anti-measles immunity in Jordan. Vaccine. 2001;19(28-29):3865-9.
72. Esteghamati A, Gouya MM, Zahraei SM, Dadras MN, Rashidi A, Mahoney F. Progress in measles and rubella elimination in Iran. The Pediatric infectious disease journal. 2007;26(12):1137-41.
73. Karimi A, Arjomandi A, Alborzi A, Rasouli M, Kadivar MR, Obood B, et al. Prevalence of measles antibody in children of different ages in Shiraz, Islamic Republic of Iran. East Mediterr Health J. 2004;10(4-5):468-73.
74. Abbassy AA, Barakat SS, Abd El Fattah MM, Said ZN, El Metwally HA. Could the MMR vaccine replace the measles vaccine at one year of age in Egypt? East Mediterr Health J. 2009;15(1):85-93.
75. Sheikh S, Ali A, Zaidi AK, Agha A, Khowaja A, Allana S, et al. Measles susceptibility in children in Karachi, Pakistan. Vaccine. 2011;29(18):3419-23.
76. Khaki M, Ghazavi A, Ghasami K, Rafiei M, Payani MA, Ghaznavi-Rad E, et al. Evaluation of viral antibodies in Iranian multiple sclerosis patients. Neurosciences (Riyadh). 2011;16(3):224-8.
77. Honarvar B, Moghadami M, Moattari A, Emami A, Odoomi N, Bagheri Lankarani K. Seroprevalence of anti-rubella and anti-measles IgG antibodies in pregnant women in Shiraz, Southern Iran: outcomes of a nationwide measles-rubella mass vaccination campaign. PloS one. 2013;8(1):e55043.
78. Yekta Z, Pourali R, Taravati MR, Shahabi S, Salary S, Khalily F, et al. Immune response to measles vaccine after mass vaccination in Urmia, Islamic Republic of Iran. East Mediterr Health J. 2009;15(3):516-25.
79. Pourabbas B, Ziyaeyan M, Alborzi A, Mardaneh J. Efficacy of measles and rubella vaccination one year after the nationwide campaign in Shiraz, Iran. Int J Infect Dis. 2008;12(1):43-6.
80. Zahraei SM, Mokhtari-Azad T, Izadi S, Mohammadi M, Sabouri A. Seroprevalence of anti-rubella and anti-measles antibodies in women at the verge of marriage in Iran. Vaccine. 2020;38(2):235-41.
81. Izadi S, Zahraei SM, Mokhtari-Azad T. Seroprevalence of antibodies to measles and rubella eight months after a vaccination campaign in the southeast of Iran. Hum Vaccin Immunother. 2018;14(6):1412-6.
82. Nikitiuk NF. [The antimeasles immunity in infants in the 1st year of life]. Zh Mikrobiol Epidemiol Immunobiol. 2000(1):63-5.
83. Gozalan A, Korukluoglu G, Kurtoglu D, Miyamura K, Yilmaz N, Morita M, et al. Measles seroepidemiology in 3 cities in Turkey. Saudi Med J. 2005;26(12):1971-7.
84. Ozbek S, Vural M, Tastan Y, Kahraman I, Perk Y, Ilter O. Passive immunity of premature infants against measles during early infancy. Acta Paediatr. 1999;88(11):1254-7.
85. Egemen A, Aksit S, Ozacar T, Kurugol Z, Keskinoglu P, Pehlivan T, et al. Measles seroprevalence in Izmir

with special emphasis on measles vaccination policy for Turkey. Pediatr Int. 2001;43(4):379-84.
86. Tanriover MD, Soyler C, Ascioglu S, Cankurtaran M, Unal S. Low seroprevalence of diphtheria, tetanus and pertussis in ambulatory adult patients: the need for lifelong vaccination. Eur J Intern Med. 2014;25(6):528-32.
87. Karaayvaz S, Oğuz MM, Beyazova U, Korukluoğlu G, Coşgun Y, Güzelküçük Z, et al. Evaluation of measles immunity in Turkey: is it still a threat? Turk J Med Sci. 2019;49(1):336-40.
88. Khetsuriani N, Chitadze N, Russell S, Ben Mamou M. Measles and rubella seroprevalence among adults in Georgia in 2015: helping guide the elimination efforts. Epidemiol Infect. 2019;147:e319.
89. Metintaş S, Akgün Y, Arslantaş D, Kalyoncu C, Uçar B. Decay of maternally derived measles antibody in central Turkey. Public health. 2002;116(1):50-4.
90. Ristić M, Milošević V, Medić S, Djekić Malbaša J, Rajčević S, Boban J, et al. Sero-epidemiological study in prediction of the risk groups for measles outbreaks in Vojvodina, Serbia. PloS one. 2019;14(5):e0216219.
91. Bekdas M, Tufan AE, Hakyemez IN, Tas T, Altunhan H, Demircioglu F, et al. Subclinical immune reactions to viral infections may correlate with child and adolescent diagnosis of attention-deficit/hyperactivity disorder: a preliminary study from Turkey. Afr Health Sci. 2014;14(2):439-45.
92. Brumboiu I, Gocan G, Bocşan IS, Soşa I, Sava A, Ursu L, et al. [Serologic assessment of measles herd immunity in the north-western region of Romania]. Rev Med Chir Soc Med Nat Iasi. 2005;109(3):616-22.
93. Kostinov MP, Zhuravlev PI, Gladkova LS, Mashilov KV, Polishchuk VB, Shmitko AD, et al. Comparative Analysis of the Measles Antibody Levels in Healthy Medical Personnel of Maternity Ward and Women in Labor. Front Immunol. 2021;12:680506.
94. Emek M, Islek D, Atasoylu G, Ozbek OA, Ceylan A, Acikgoz A, et al. Association between seroprevalence of measles and various social determinants in the year following a measles outbreak in Turkey. Public health. 2017;147:51-8.
95. Kanra G, Tezcan S, Badur S. Hepatitis B and measles seroprevalence among Turkish children. Turk J Pediatr. 2005;47(2):105-10.
96. Gunes T, Koklu E, Ozturk MA, Akcakus M, Kurtoglu S, Cetin N, et al. Antimeasles antibodies in preterm infants during early infancy in Turkey. Ann Trop Paediatr. 2007;27(1):31-7.
97. Sorodoc Y, Cepleanu M, Cernescu C, Cajal N. [Sero-epidemiological data on measles in Rumania]. Stud Cercet Virusol. 1973;24(4):311-7.
98. Mal'tseva NN, Desiatskova RG, Kul'kova SA, Egorov VM, Makarova EM. [Assessment by immunoenzyme analysis of the immune status of donors with reference to the viruses of rubella, measles and herpes simplex]. Zh Mikrobiol Epidemiol Immunobiol. 1988(1):55-8.
99. Bhaskaram P, Radhakrishna KV, Madhusudan J. Seroepidemiological study to determine age for measles vaccination. Indian J Med Res. 1986;83:480-6.
100. Cherian T, Joseph A, John TJ. Low antibody response in infants with measles and children with subclinical measles virus infection. J Trop Med Hyg. 1984;87(1):27-31.
101. Khan Z, Malik A, Pal SD, Khan MY. Measles antibody titre in children up to 5 years of age in rural areas of Aligarh District, India. Saudi Med J. 2000;21(2):175-9.
102. Khare S, Dutta A, Kumari S, Basu RN. Seroepidemiology of measles in Delhi: implications for age of vaccination. Indian J Pediatr. 1987;54(5):711-5.
103. John TJ, Jesudoss ES. A survey of measles antibody in children. Indian Pediatr. 1973;10(2):65-6.
104. Dongre ST, Biranjan JR, Jalgaonkar SV, Polakhare JK. Measles antibody levels in 3 to 9 months old infants. Indian Pediatr. 1989;26(8):775-9.
105. Broor S, Pal SR, Banerjee AK, Chitkara NL, Choudhury S. Sero-epidemiological study of measles virus infection in and around Chandigarh. Indian J Med Res. 1976;64(12):1740-6.
106. Ueda S, Okuno Y, Sangkawibha N, Jayavasu J, Tuchinda P. Studies on measles in Thailand. 1. Seroepidemiological examination. Biken J. 1967;10(3):129-33.
107. Saipan P, Jiwapaisarnpong T, Pattanadilok S, Loyha Y, Janggajit T. Measles antibody in the children in Ubon Ratchathani province. J Med Assoc Thai. 2001;84(4):500-6.
108. Vanprapar N, Chavalittamrong B, Chearskul S, Pimolpan V. Disappearance of measles antibody in Thai infants after birth. Southeast Asian J Trop Med Public Health. 1983;14(4):488-90.
109. Bhardwaj AK, Swami HM, Gupta BP, Saha SM, Ahluwalia SK, Vaidya NK. Sero-epidemiological survey

of measles in a tribal district of Himachal Pradesh. J Commun Dis. 1988;20(4):316-20.
110. Sehgal S, Sharma RS, Mehta PK, Sebastian M, Arora RR. Sero-epidemiological survey of measles. J Commun Dis. 1983;15(1):1-7.
111. Gohil DJ, Kothari ST, Chaudhari AB, Gunale BK, Kulkarni PS, Deshmukh RA, et al. Seroprevalence of Measles, Mumps, and Rubella Antibodies in College Students in Mumbai, India. Viral Immunol. 2016;29(3):159-63.
112. Muthiah N, Galagoda G, Handunnetti S, Peiris S, Pathirana S. Dynamics of maternally transferred antibodies against measles, mumps, and rubella in infants in Sri Lanka. Int J Infect Dis. 2021;107:129-34.
113. Wanlapakorn N, Wasitthankasem R, Vichaiwattana P, Auphimai C, Yoocharoen P, Vongpunsawad S, et al. Antibodies against measles and rubella virus among different age groups in Thailand: A population-based serological survey. PloS one. 2019;14(11):e0225606.
114. Chaiwarith R, Praparattanapan J, Nuket K, Kotarathitithum W, Supparatpinyo K. Seroprevalence of antibodies to measles, mumps, and rubella, and serologic responses after vaccination among human immunodeficiency virus (HIV)-1 infected adults in Northern Thailand. BMC Infect Dis. 2016;16:190.
115. Murray AF, Englund JA, Tielsch JM, Katz J, Shrestha L, Khatry SK, et al. Measles and Rubella Seroprevalence in Mother-Infant Pairs in Rural Nepal and the United States: Pre- and Post-Elimination Populations. Am J Trop Med Hyg. 2018;99(5):1342-5.
116. Sathiyanarayanan S, Kumar P, Rao CR, Kumar A, Kamath A, Kamath V. Prevalence of Maternal Measles Antibody and Its Associated Factors among Infants in Coastal Karnataka, India. Indian J Community Med. 2020;45(1):83-8.
117. Tharmaphornpilas P, Yoocharean P, Rasdjarmrearnsook AO, Theamboonlers A, Poovorawan Y. Seroprevalence of antibodies to measles, mumps, and rubella among Thai population: evaluation of measles/MMR immunization programme. J Health Popul Nutr. 2009;27(1):80-6.
118. Sood DK, Kumar S, Singh S, Sharma SB, Sokhey J, Singh H. Transplacental immunity and waning of maternal antibody in measles. Indian J Pediatr. 1995;62(1):95-9.
119. Mehta NA, Nanavati AN, Jhala HI, Sant MV. Seroepidemiology of measles in Bombay. Indian J Med Res. 1972;60(5):661-9.
120. Gonwong S, Chuenchitra T, Khantapura P, Islam D, Mason CJ. Measles susceptibility in young Thai men suggests need for young adult measles vaccination: a cross sectional study. BMC Public Health. 2016;16:309.
121. Gupta M, Tripathy JP, Verma M, Singh MP, Kaur R, Ratho RK, et al. Seroprevalence of measles, mumps & rubella antibodies among 5-10 years old children in north India. Indian J Med Res. 2019;149(3):396-403.
122. Wangchuk S, Nogareda F, Tshering N, Khandu L, Pelden S, Wannemuehler K, et al. Measles and rubella immunity in the population of Bhutan, 2017. Vaccine. 2019;37(43):6463-9.
123. Saha SM, Balasubrahmanyan M, Kambo BS, Ram D. Sero epidemiological surgery of measles in India. Indian J Pathol Microbiol. 1980;23(4):231-4.
124. Sharma RS, Ray K, Grover SS, Behl JP. Sero-epidemiological survey of measles in Alwar, Rajasthan. J Commun Dis. 1986;18(1):6-8.
125. Willis MF, Warburton MF. Measles susceptibility in two Pacific atoll populations. Epidemiological factors and response to live attenuated measles virus. Med J Aust. 1974;1(20):789-93.
126. Sultana R, Rahman MM, Hassan Z, Hassan MS. Prevalence of IgG antibody against measles, mumps and rubella in bangladeshi children: a pilot study to evaluate the need for integrated vaccination strategy. Scand J Immunol. 2006;64(6):684-9.
127. Breakwell L, Anga J, Cooley G, Ropiti L, Gwyn S, Wannemuehler K, et al. Seroprevalence of chronic hepatitis B virus infection and immunity to measles, rubella, tetanus and diphtheria among schoolchildren aged 6-7 years old in the Solomon Islands, 2016. Vaccine. 2020;38(30):4679-86.
128. Yu X, Wang S, Guan J, Mahemuti, Purhati, Gou A, et al. Analysis of the cause of increased measles incidence in Xinjiang, China in 2004. The Pediatric infectious disease journal. 2007;26(6):513-8.
129. Zhang XJ, Zhao HQ. [Sero-epidemiology of measles in Shijiazhuang District after a planned 9-year immunization program]. Zhonghua Liu Xing Bing Xue Za Zhi. 1985;6(1):1-3.
130. Chong KC, Rui Y, Liu Y, Zhou T, Jia K, Wang MH, et al. Early Waning of Maternal Measles Antibodies in Infants in Zhejiang Province, China: A Comparison of Two Cross-Sectional Serosurveys. Int J Environ Res Public

Health. 2019;16(23).
131. Saraswathy TS, Sinniah M, Lee WS, Lye MS, Choo KE, Jusoh H. Poliomyelitis and measles serosurvey in northern Malaysia. Southeast Asian J Trop Med Public Health. 1994;25(3):565-8.
132. Liu Y, Lu P, Hu Y, Wang Z, Deng X, Ma F, et al. Cross-sectional surveys of measles antibodies in the Jiangsu Province of China from 2008 to 2010: the effect of high coverage with two doses of measles vaccine among children. PloS one. 2013;8(6):e66771.
133. Zhu Q, Hu TJ, Shen JW, Shen JH, Chen WH, Gu SK, et al. [A study on measles and rubella antibody level in 319 pairs of mothers and infants in Songjiang District of Shanghai]. Zhonghua Yu Fang Yi Xue Za Zhi. 2019;53(4):388-93.
134. Hao YW. [Seroepidemiology of measles in 1428 people from a copper mine of Chengde City]. Zhonghua Liu Xing Bing Xue Za Zhi. 1985;6(5):289-91.
135. Mao B, Chheng K, Wannemuehler K, Vynnycky E, Buth S, Soeung SC, et al. Immunity to polio, measles and rubella in women of child-bearing age and estimated congenital rubella syndrome incidence, Cambodia, 2012. Epidemiol Infect. 2015;143(9):1858-67.
136. Wang Z, Yan R, He H, Li Q, Chen G, Yang S, et al. Difficulties in eliminating measles and controlling rubella and mumps: a cross-sectional study of a first measles and rubella vaccination and a second measles, mumps, and rubella vaccination. PloS one. 2014;9(2):e89361.
137. Xu FG. [Outbreak of measles in a rural district and its sero-epidemiological analysis]. Zhonghua Liu Xing Bing Xue Za Zhi. 1983;4(1):8-11.
138. Pei L, Yang Y, Zhao X, Zhang S, Yuan L, Liu Y, et al. Identify the susceptibility profile to measles in the general population: Serological survey of measles antibodies in Shaanxi province, China, in 2016. Vaccine. 2017;35(52):7250-5.
139. Ma C, Li F, Zheng X, Zhang H, Duan M, Yang Y, et al. Measles vaccine coverage estimates in an outbreak three years after the nation-wide campaign in China: implications for measles elimination, 2013. BMC Infect Dis. 2015;15:23.
140. Ma YJ, Bo F, Sun ZD, Huang H, Gao SR, An ZJ. Epidemiological investigation of measles in sera of healthy people in Heilongjiang Province, China. Jpn J Infect Dis. 2011;64(3):208-10.
141. He H, Chen EF, Li Q, Wang Z, Yan R, Fu J, et al. Waning immunity to measles in young adults and booster effects of revaccination in secondary school students. Vaccine. 2013;31(3):533-7.
142. Ding Y, Chen W, Lei Y, Mao N, Gao Z, Xu W, et al. Evaluating the population measles susceptibility in Tianjin, China. Vaccine. 2020;38(31):4829-36.
143. Khampanisong P, Pauly M, Nouanthong P, Vickers MA, Virachith S, Xaydalasouk K, et al. Waning of Maternal Antibodies against Measles Suggests a Large Window of Susceptibility in Infants in Lao People's Democratic Republic. Pathogens. 2021;10(10).
144. Ma C, Hao L, Rodewald L, An Q, Wannemuehler KA, Su Q, et al. Risk factors for measles virus infection and susceptibility in persons aged 15 years and older in China: A multi-site case-control study, 2012-2013. Vaccine. 2020;38(16):3210-7.
145. Xiong Y, Wang D, Lin W, Tang H, Chen S, Ni J. Age-related changes in serological susceptibility patterns to measles: results from a seroepidemiological study in Dongguan, China. Hum Vaccin Immunother. 2014;10(4):1097-03.
146. Chong KC, Zhang C, Zee BC, Luo T, Wang L, Tam GC, et al. Interpreting the transmissibility of measles in two different post periods of supplementary immunization activities in Hubei, China. Vaccine. 2017;35(7):1024-9.
147. Nogareda F, Gunregjav N, Sarankhuu A, Munkhbat E, Ichinnorov E, Nymadawa P, et al. Measles and rubella IgG seroprevalence in persons 6 month-35 years of age, Mongolia, 2016. Vaccine. 2020;38(26):4200-8.
148. Zhao ZX, Zhou RR, Li LQ, Yu W, Li QF, Hu P. [Analysis of measles immunity level and serological susceptibility among Yunnan residents aged ≥20 years]. Zhonghua Yu Fang Yi Xue Za Zhi. 2018;52(1):50-4.
149. Wagner AL, Boulton ML, Gillespie BW, Zhang Y, Ding Y, Carlson BF, et al. Risk factors for measles among adults in Tianjin, China: Who should be controls in a case-control study? PloS one. 2017;12(9):e0185465.
150. Yan R, He H, Zhou Y, Xie S, Deng X, Tang X. Study on factors associated with seroprotection after

measles vaccination in children of 6-14 years in Eastern China. Vaccine. 2019;37(36):5185-90.
151. Choisy M, Trinh ST, Nguyen TND, Nguyen TH, Mai QL, Pham QT, et al. Sero-Prevalence Surveillance to Predict Vaccine-Preventable Disease Outbreaks; A Lesson from the 2014 Measles Epidemic in Northern Vietnam. Open Forum Infect Dis. 2019;6(3):ofz030.
152. Li J, Lu L, Chen M, Huang F, Zeng Y, Li XM, et al. [Analysis of measles immunity level in persistent populations in Beijing, 2012]. Zhonghua Yu Fang Yi Xue Za Zhi. 2013;47(10):916-9.
153. Boulton ML, Wang X, Zhang Y, Montgomery JP, Wagner AL, Carlson BF, et al. A population profile of measles susceptibility in Tianjin, China. Vaccine. 2016;34(27):3037-43.
154. Wang Q, Cheng X, Liu D, Chen C, Yao K. One single-center serological survey on measles, rubella and mumps antibody levels of people in Youyang, China. Hum Vaccin Immunother. 2021;17(11):4203-9.
155. Han K, Chen S, Tang C, Wen J, Li J, Ni J, et al. The epidemiological and serological characteristics of measles in Dongguan, China, 2005-2014. Hum Vaccin Immunother. 2016;12(8):2181-7.
156. Lu L, Cao YM, Yang QY, He Q, Dong ZQ, Di B, et al. [Dynamic maternal measles antibody level in infants: a longitudinal study]. Zhonghua Liu Xing Bing Xue Za Zhi. 2016;37(5):663-7.
157. Fu C, Lu L, Wu H, Shaman J, Cao Y, Fang F, et al. Placental antibody transfer efficiency and maternal levels: specific for measles, coxsackievirus A16, enterovirus 71, poliomyelitis I-III and HIV-1 antibodies. Sci Rep. 2016;6:38874.
158. Zhang Z, Chen M, Wang Y, Li J, Li X, Lu L. Seroepidemiology of measles in Beijing, China: a cross-sectional study. Hum Vaccin Immunother. 2019;15(9):2112-6.
159. Zhang X, Kou G, Du H, Ju Z, Zhong L, Cui X, et al. Measles epidemiology and survey of measles immunity level among healthy population in Baoji City, Shaanxi Province, China. Jpn J Infect Dis. 2013;66(5):449-53.
160. Hazlina Y, Marlindawati MA, Shamsuddin K. Serological assessment of the establishment of herd immunity against measles in a health district in Malaysia. BMC Infect Dis. 2016;16(1):740.
161. Xu GZ, Ma R, Xu HJ, Ma YH, Dong HJ, Li Y, et al. [Levels of transition on maternal transferred measles antibody in infants in 3 cities in China]. Zhonghua Liu Xing Bing Xue Za Zhi. 2008;29(11):1074-7.
162. Tang L, Zhou Y, Pan Y, Zhu H. Measles epidemics and seroepidemiology of population in Wujin, Changzhou city, Jiangsu province, China 2015. Vaccine. 2017;35(22):2925-9.
163. Meng QH, Liu Y, Yu JQ, Li LJ, Shi W, Shen YJ, et al. Seroprevalence of Maternal and Cord Antibodies Specific for Diphtheria, Tetanus, Pertussis, Measles, Mumps and Rubella in Shunyi, Beijing. Sci Rep. 2018;8(1):13021.
164. Xaydalasouk K, Sayasinh K, Hübschen JM, Khounvisith V, Keomany S, Muller CP, et al. Age-stratified seroprevalence of vaccine-preventable infectious disease in Saravan, Southern Lao People's Democratic Republic. Int J Infect Dis. 2021;107:25-30.
165. Withers BG, Kelley PW, Pang LW, Kustermann JA, MacArthy PO, Russell BJ, et al. Vaccine-preventable disease susceptibility in a young adult Micronesian population. Southeast Asian J Trop Med Public Health. 1994;25(3):569-74.
166. Fu H, Abbas K, Klepac P, van Zandvoort K, Tanvir H, Portnoy A, et al. Effect of evidence updates on key determinants of measles vaccination impact: a DynaMICE modelling study in ten high-burden countries. BMC Med. 2021;19(1):281.
