# Supplementary figures and images for: Estimating the basic reproduction number of measles in low-and middle-income settings using 172 seroprevalence studies: A modelling approach
Source: PLOS Glob Public Health. 2026 Jul 17;6(7):e0006731. doi: 10.1371/journal.pgph.0006731 (PMC13378968; doi:10.1371/journal.pgph.0006731)

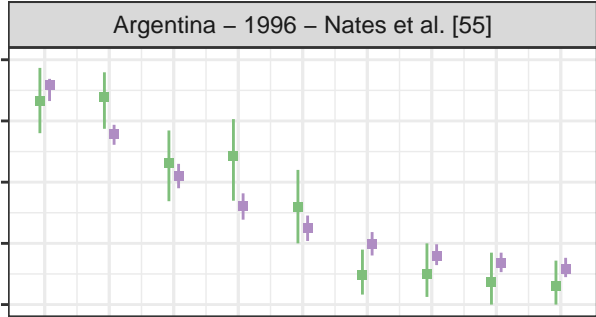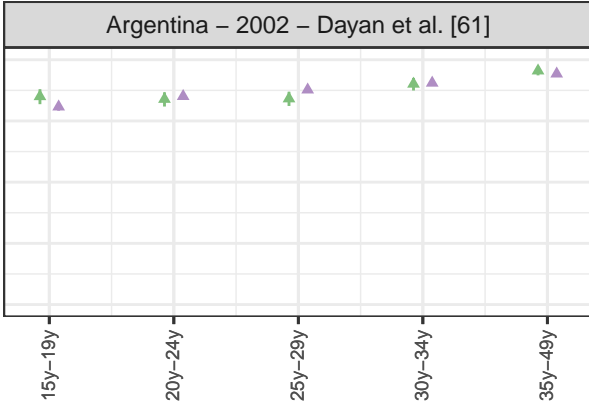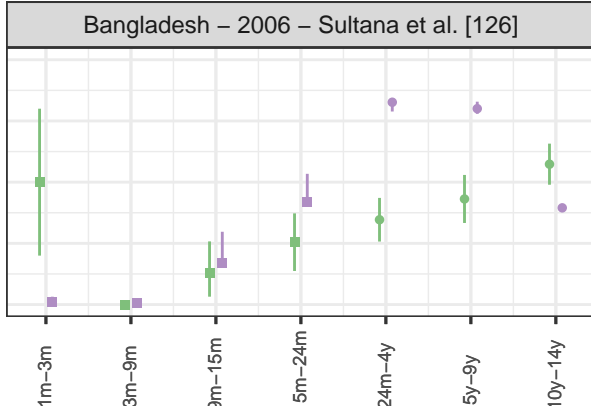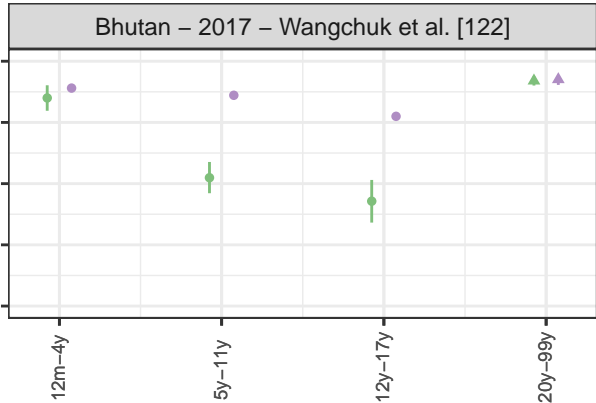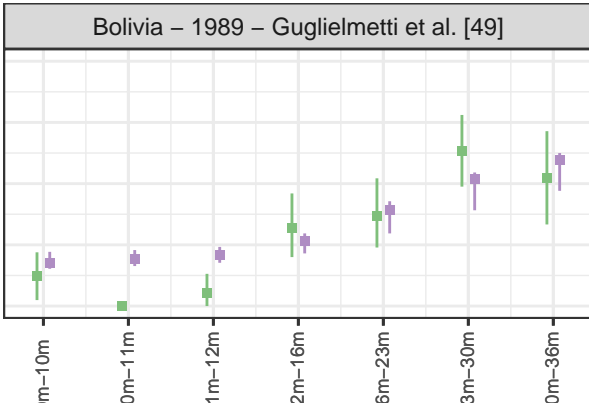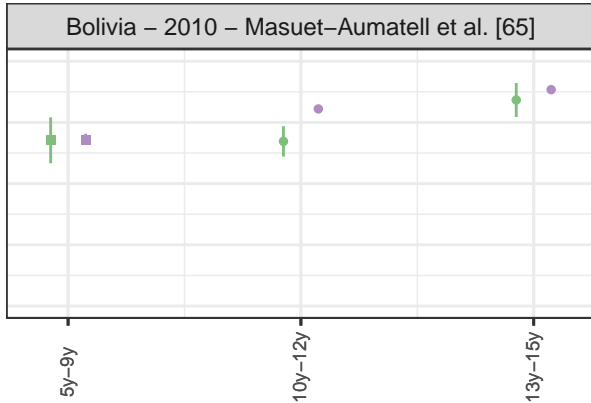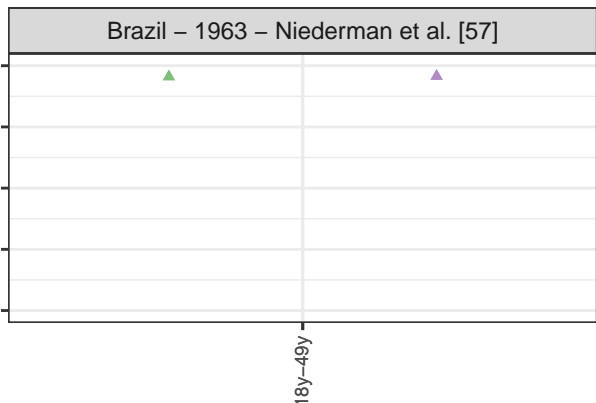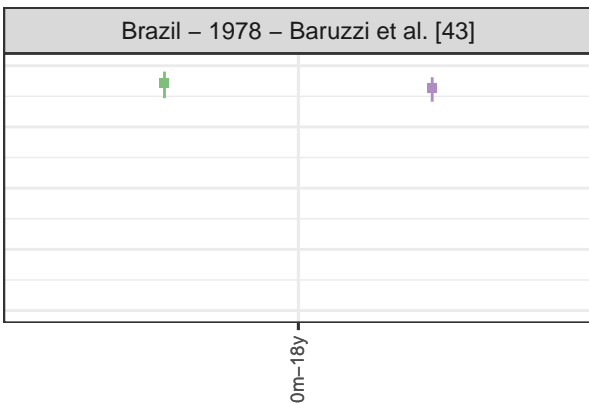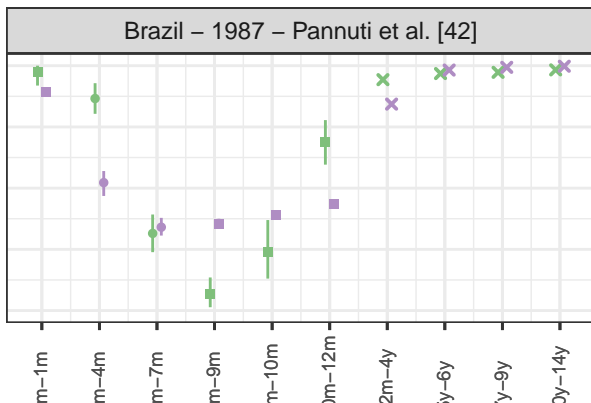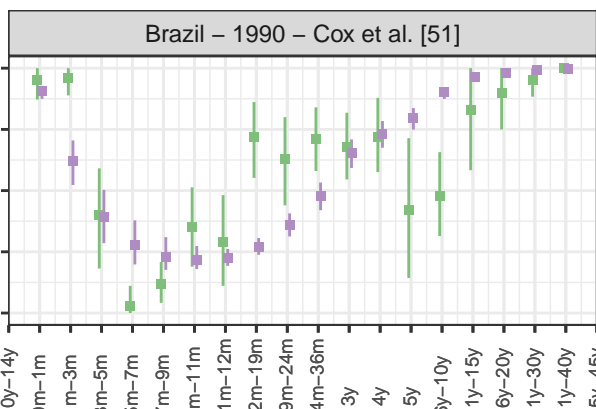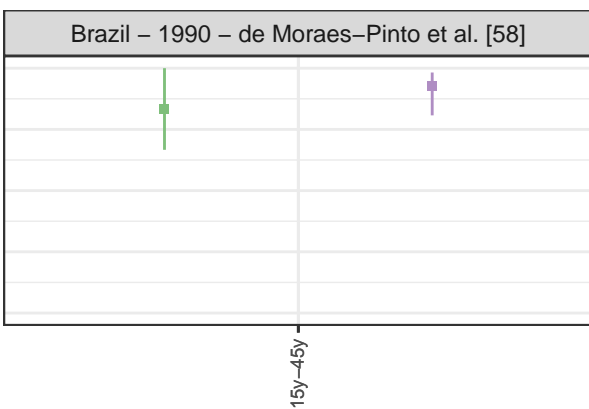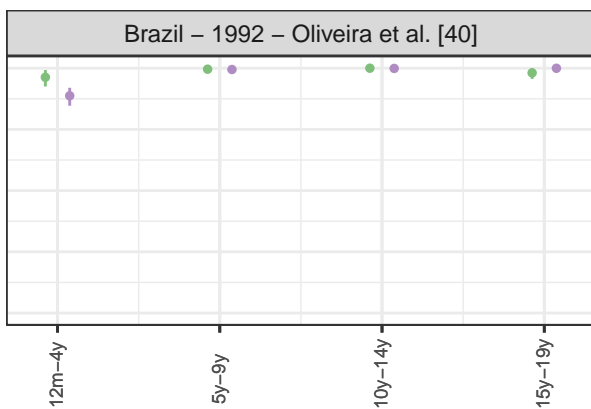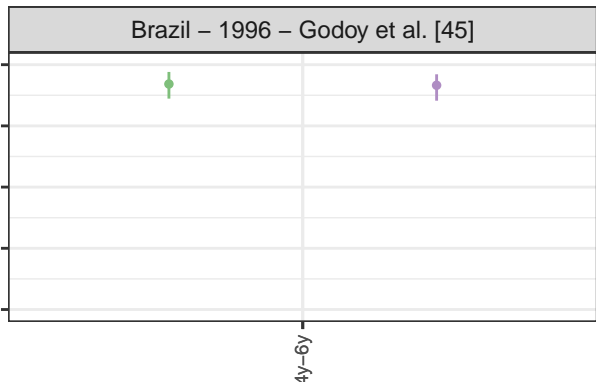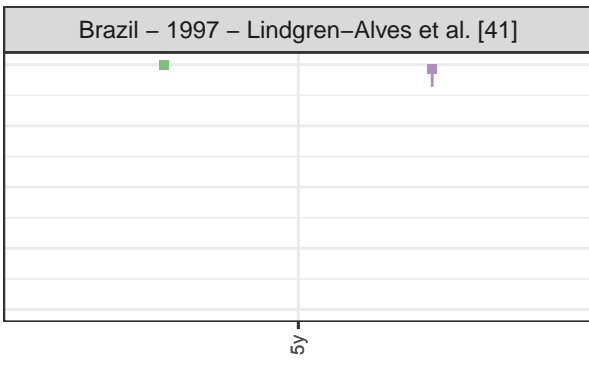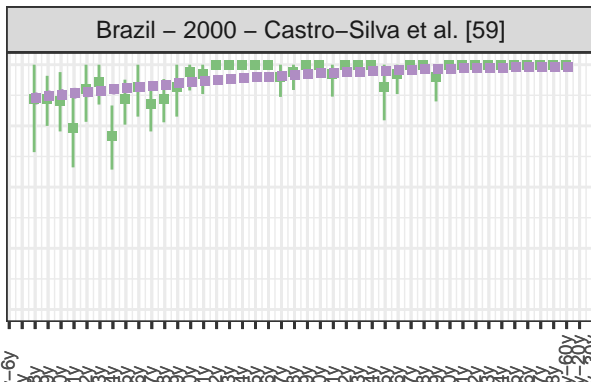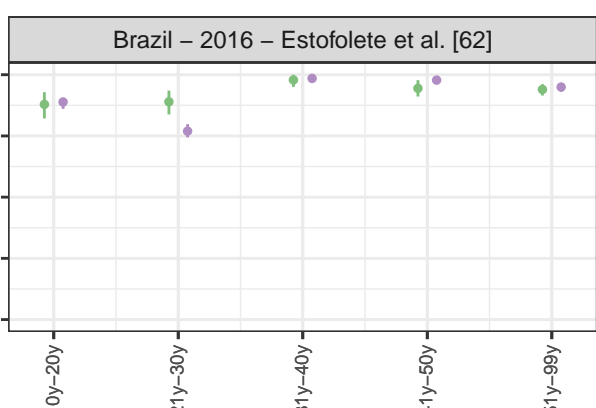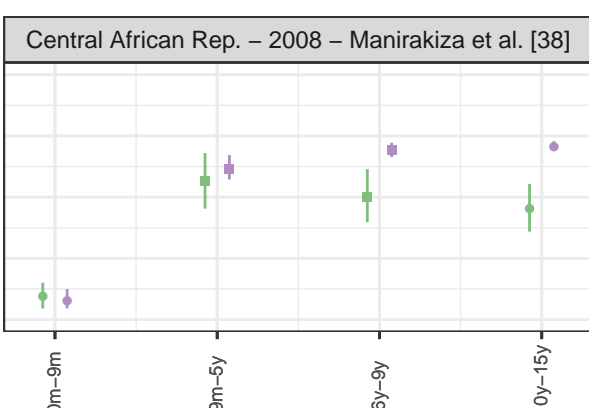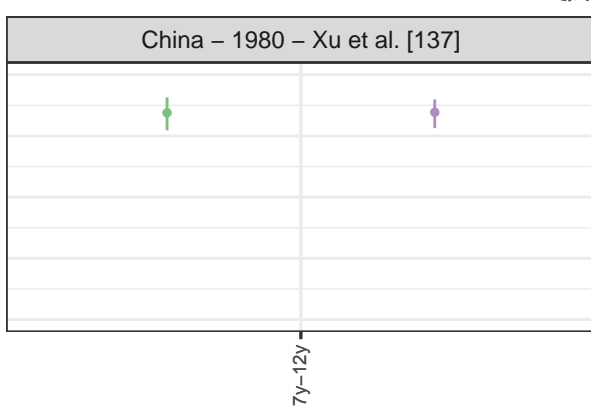

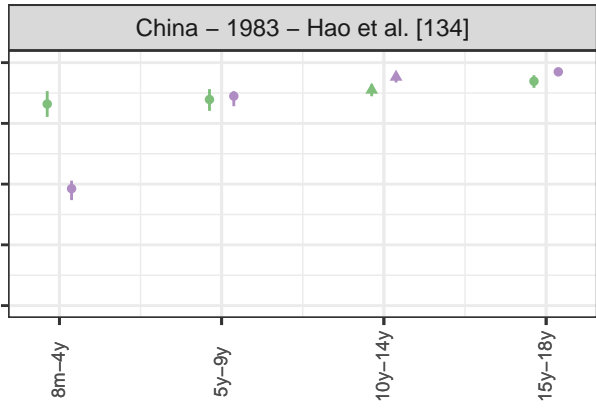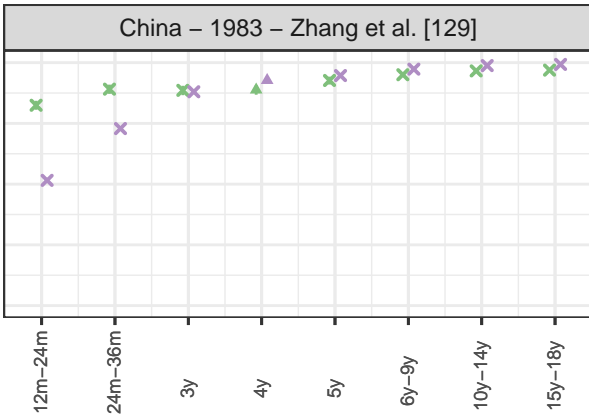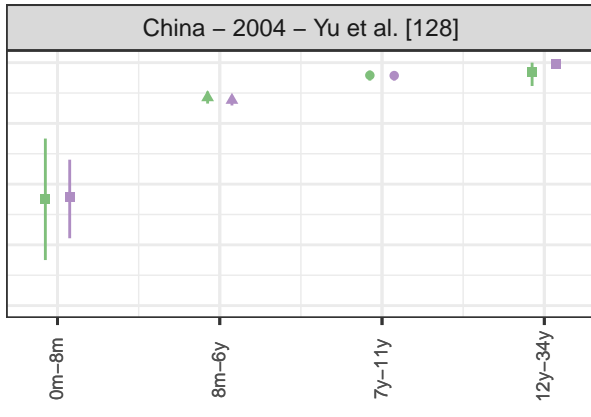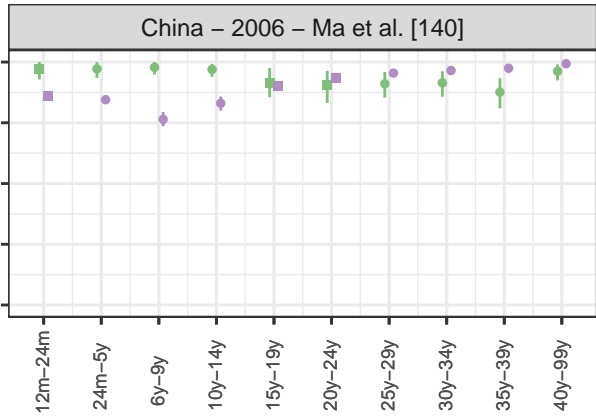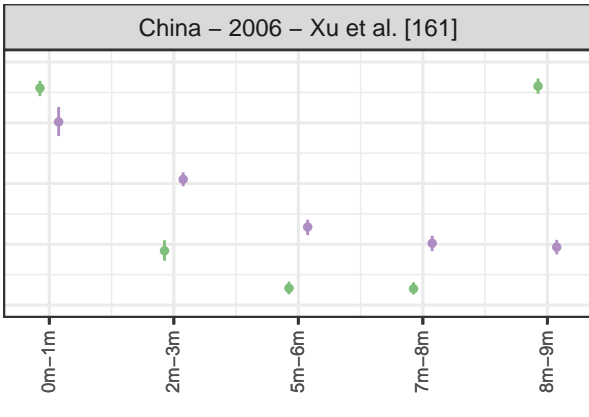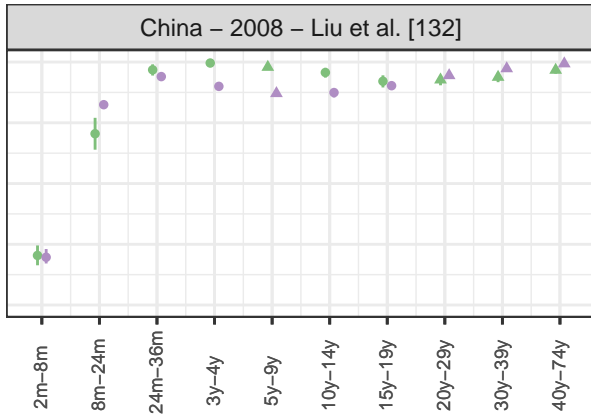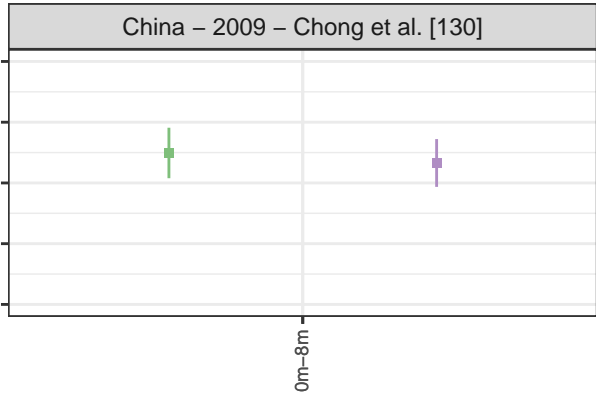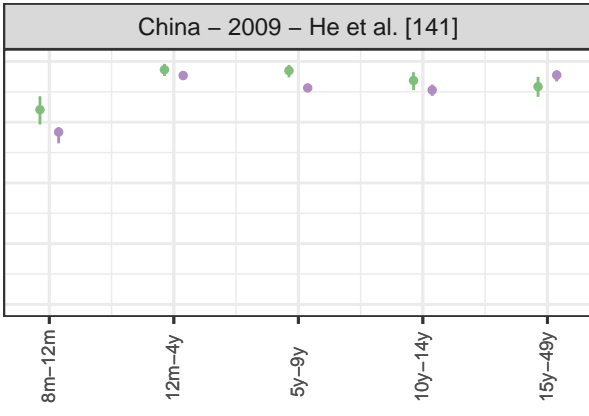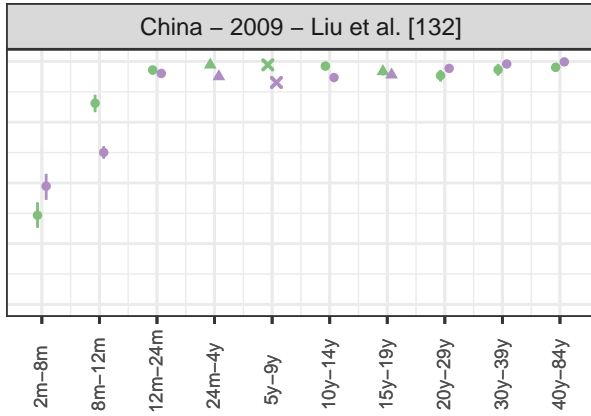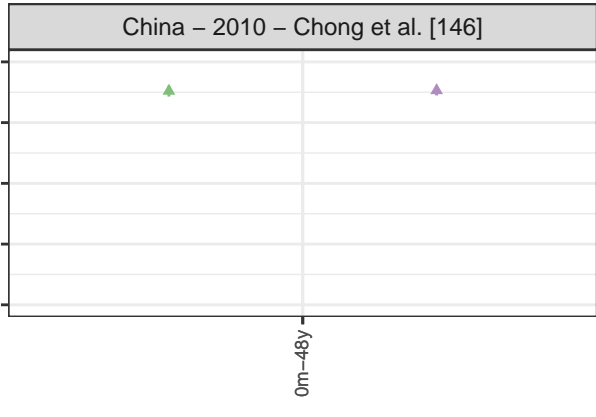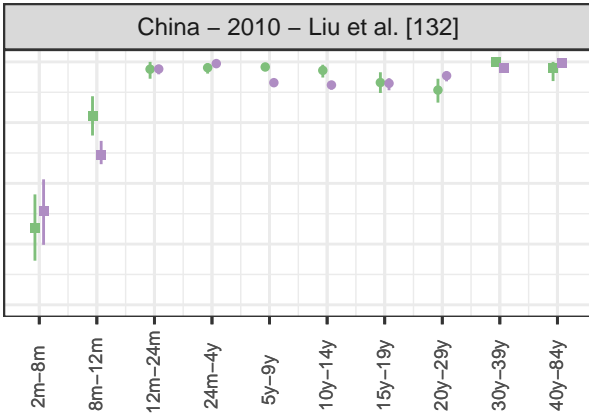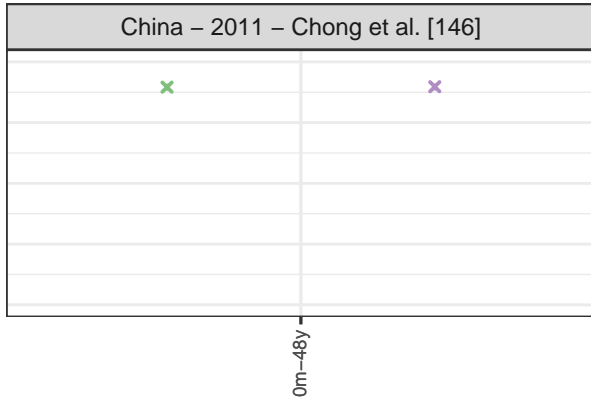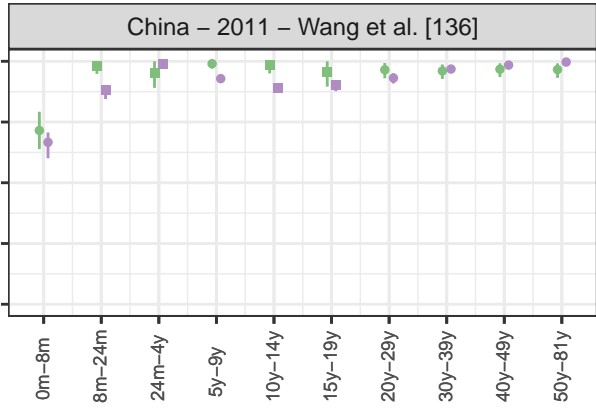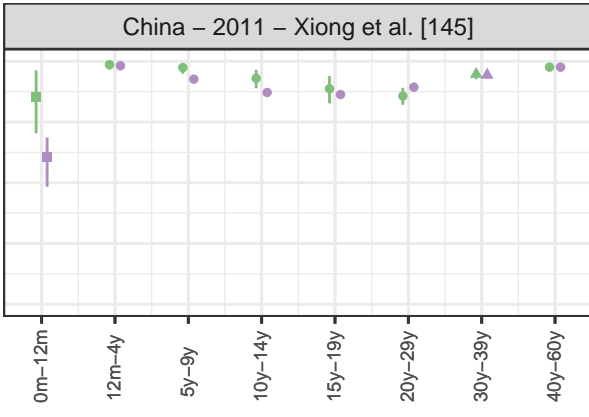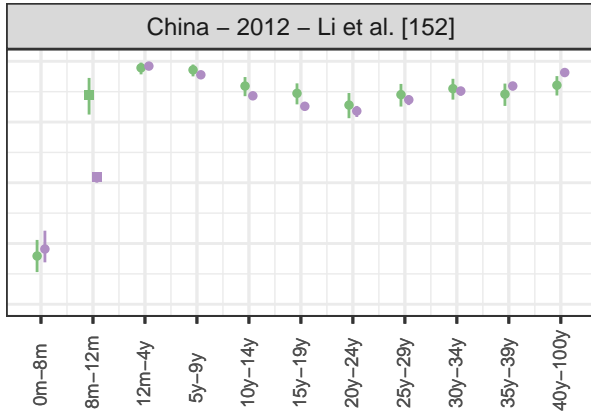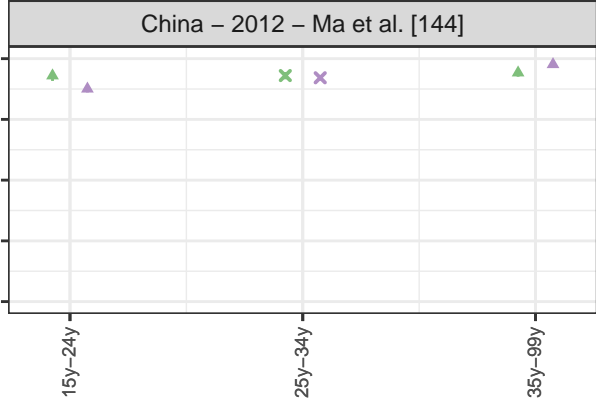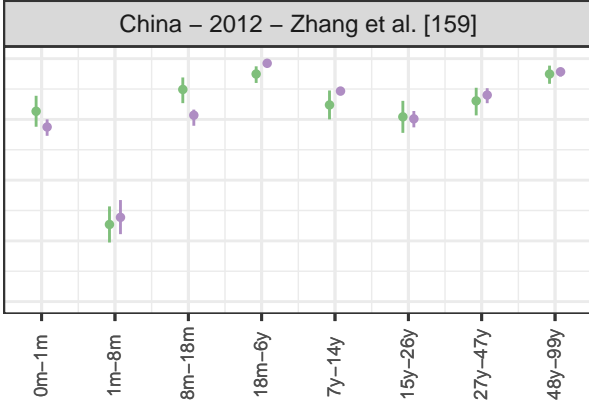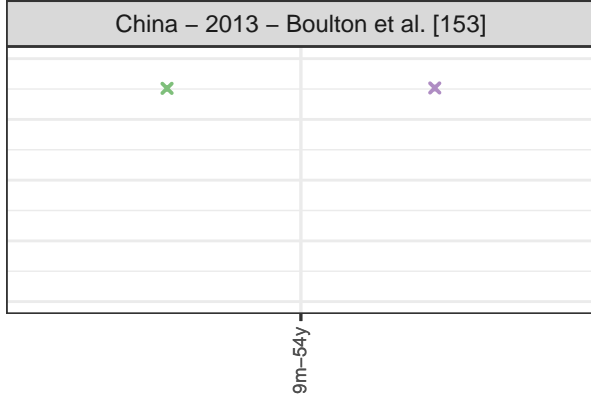

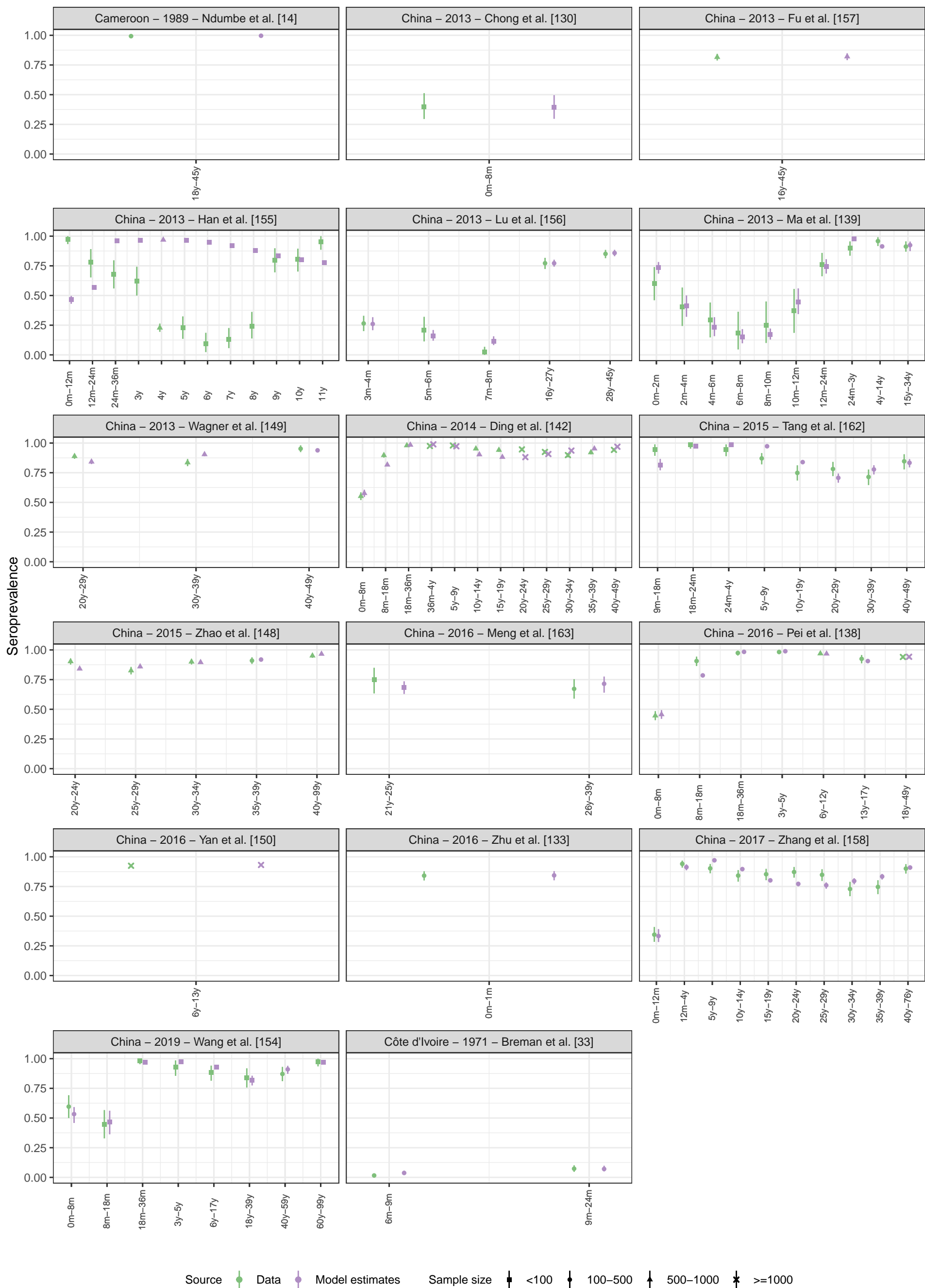

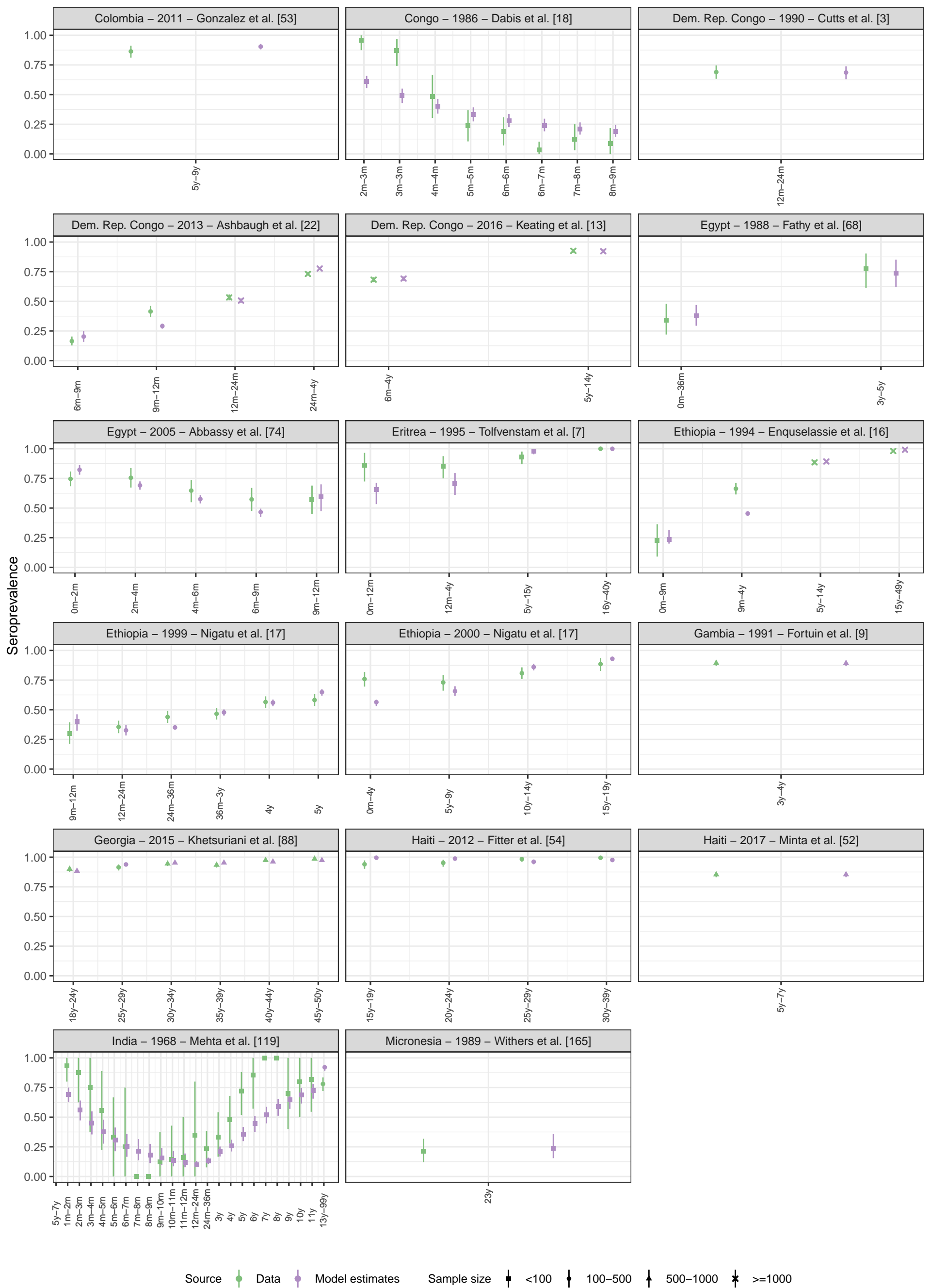

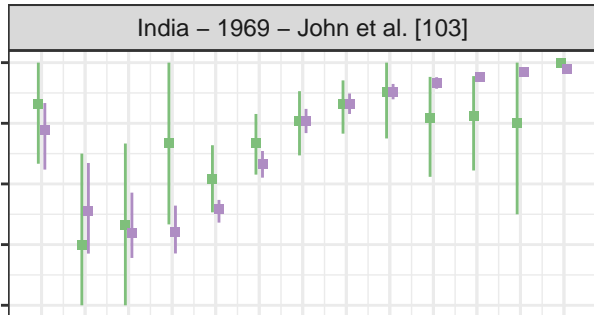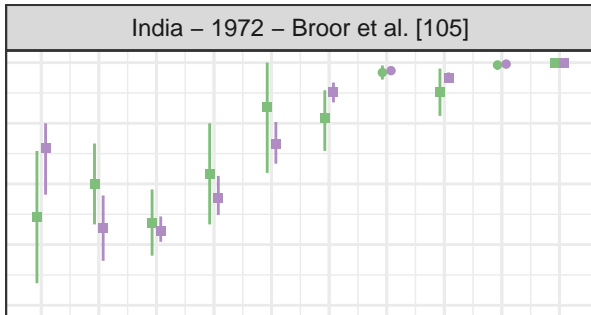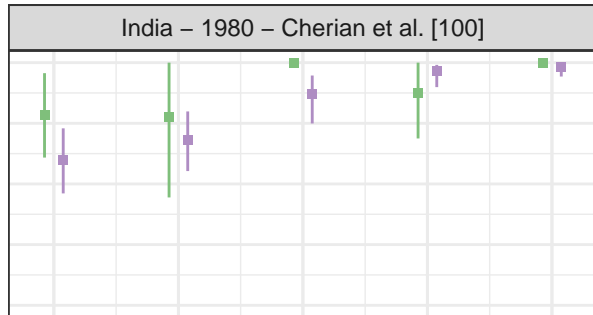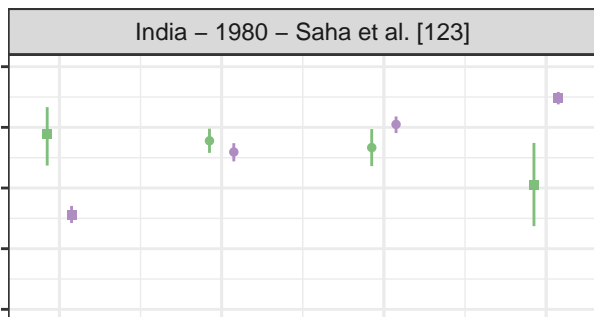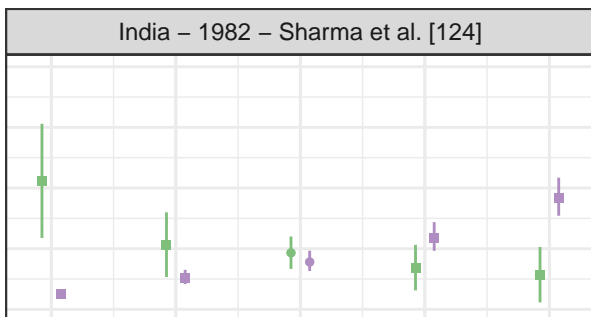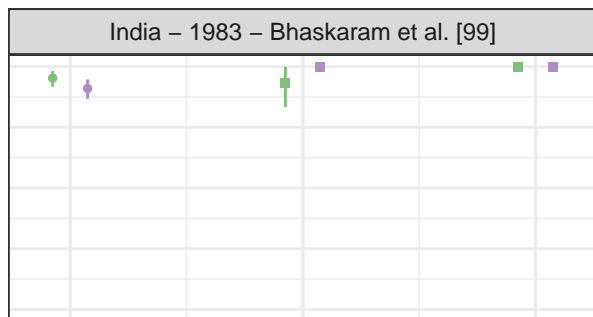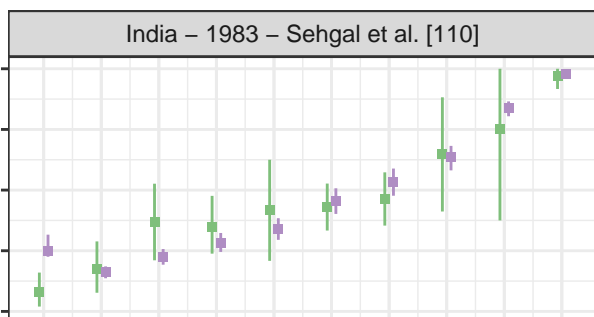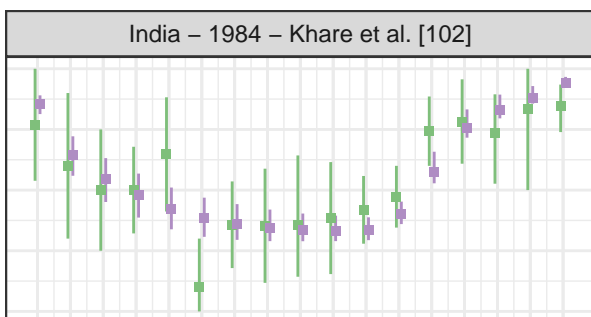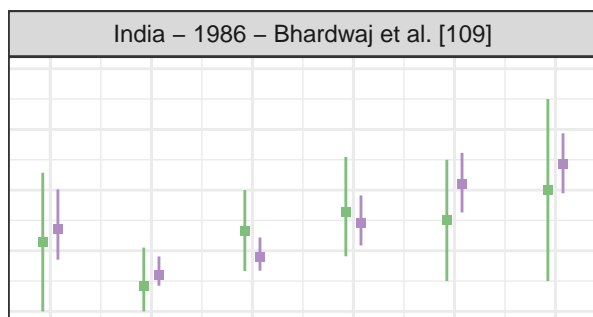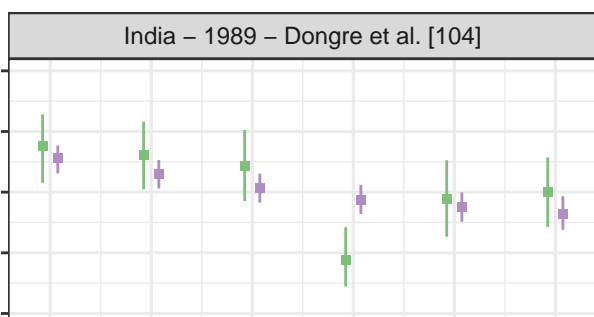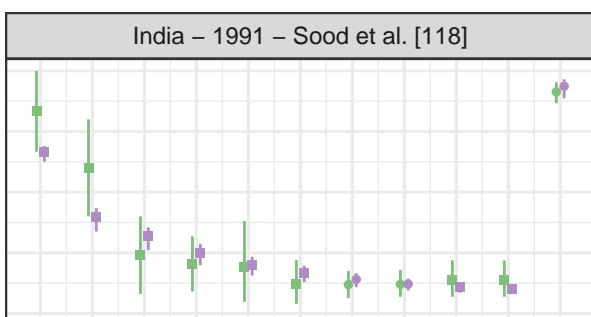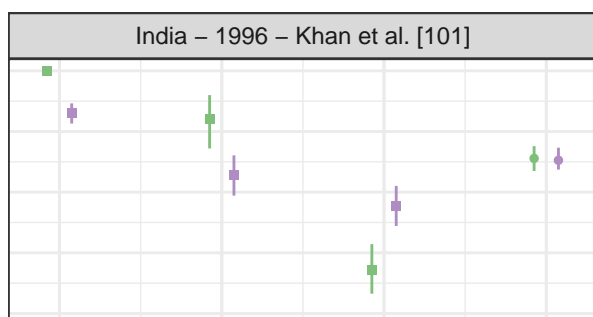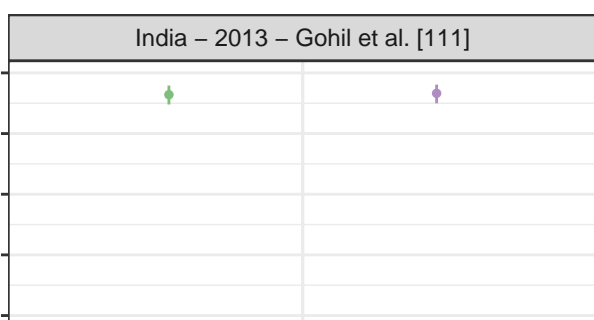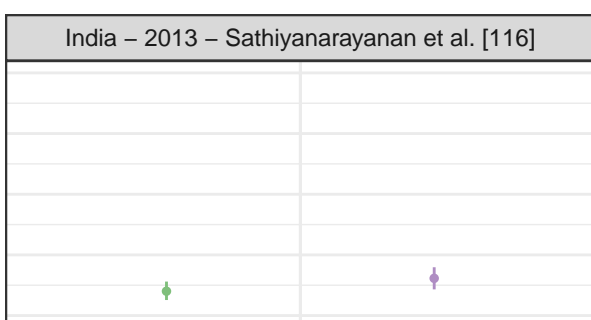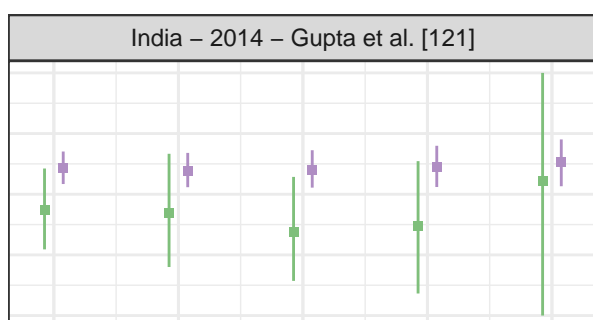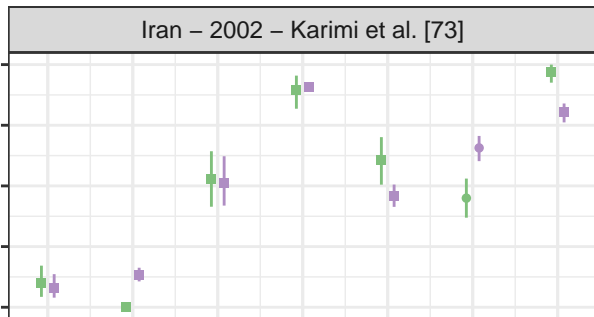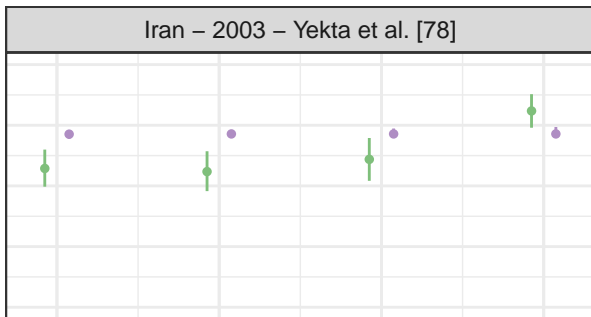

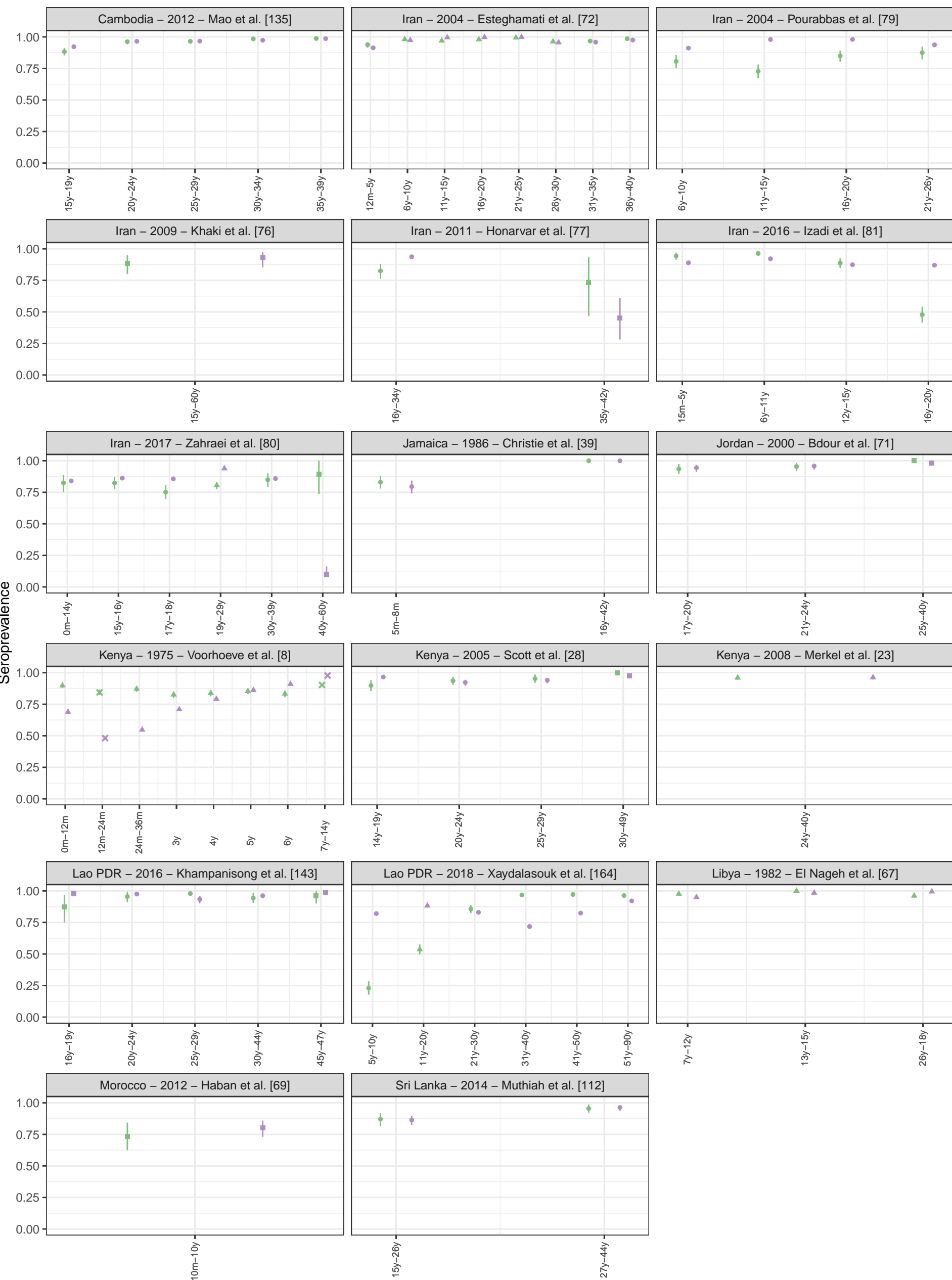

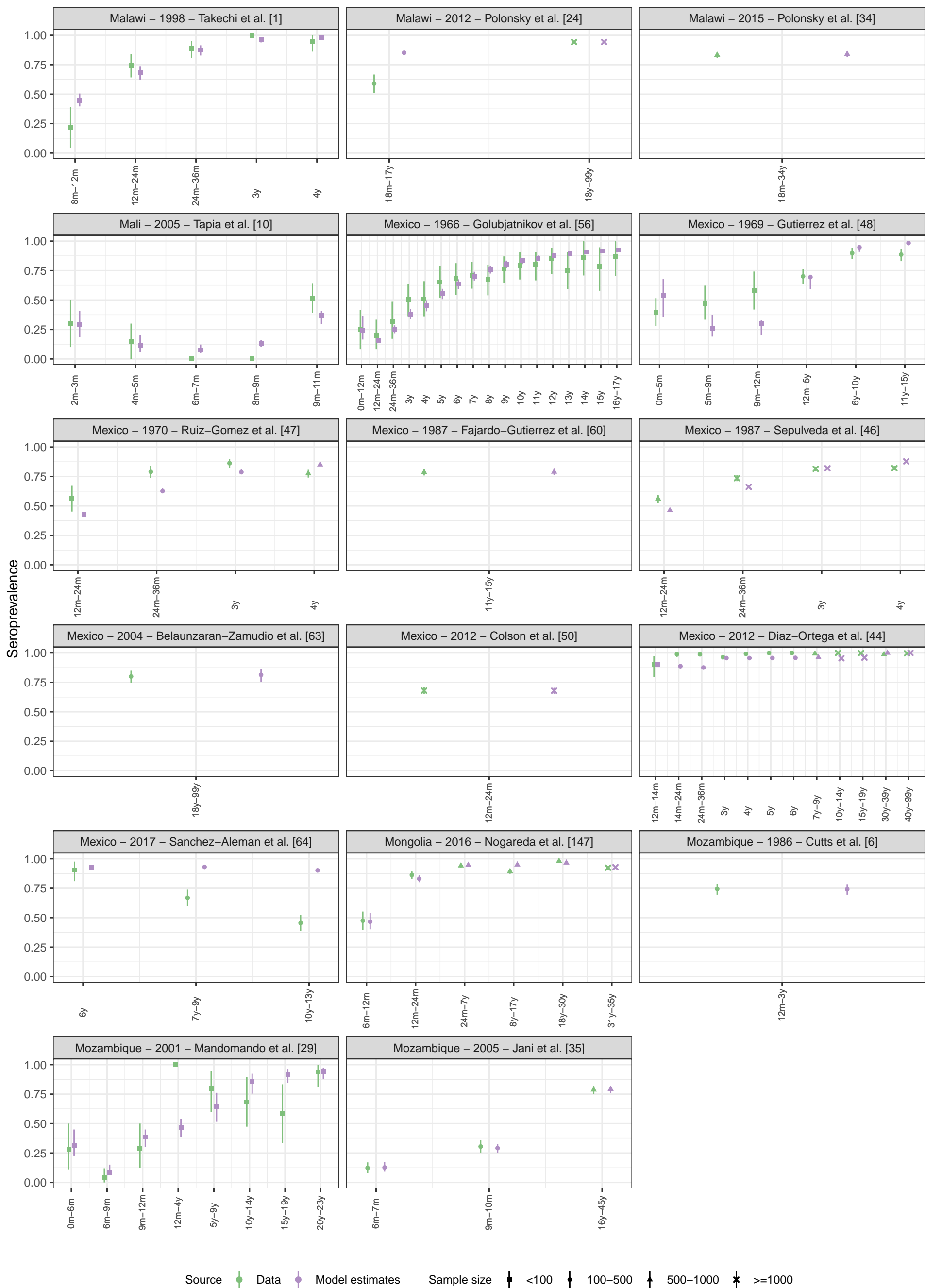

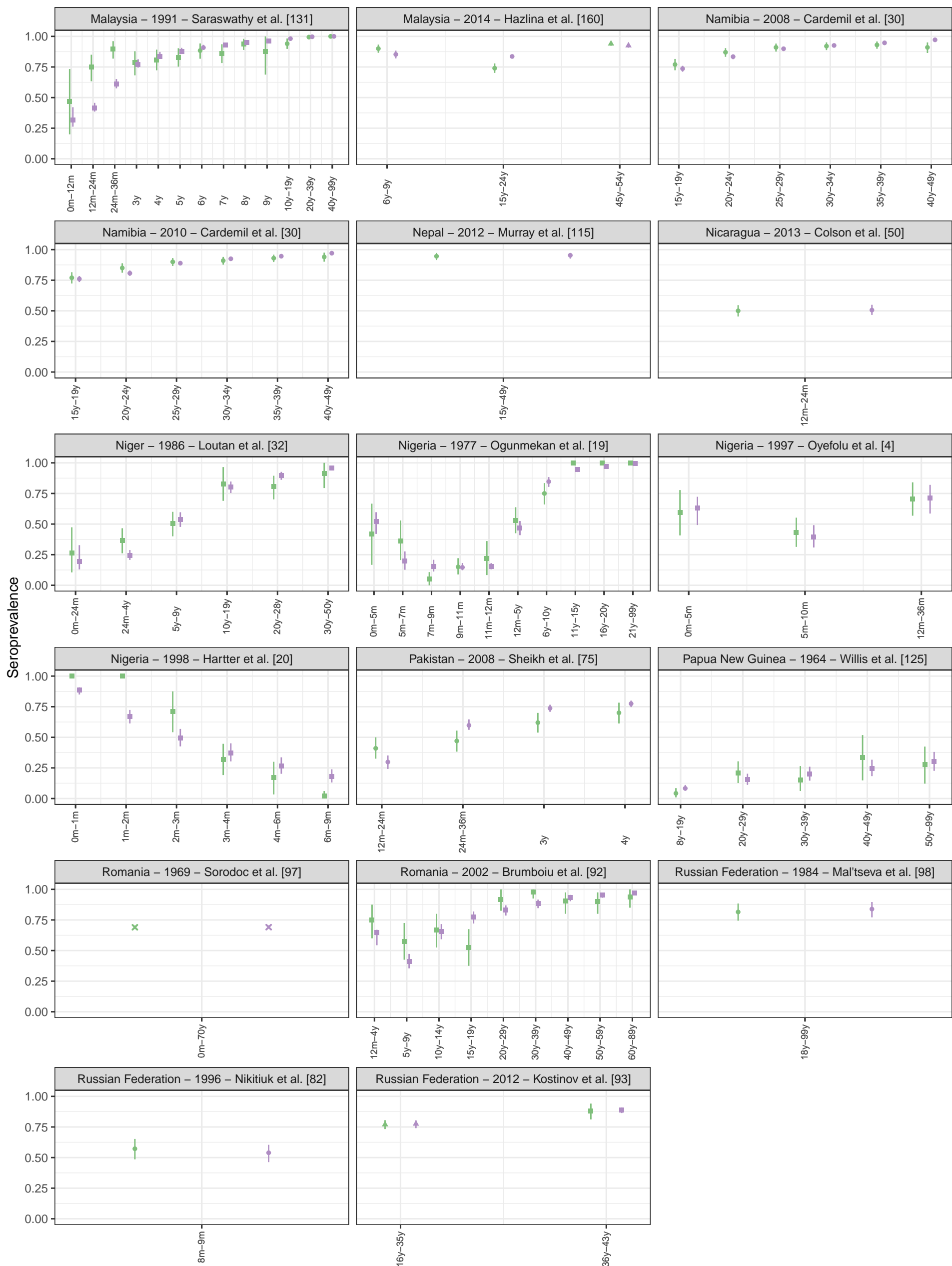

Source Data Model estimates Sample size <100 100–500 500–1000 >=1000

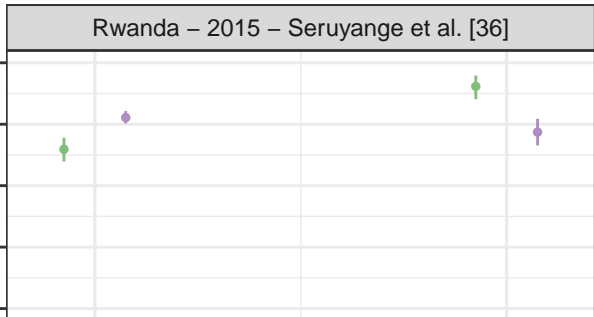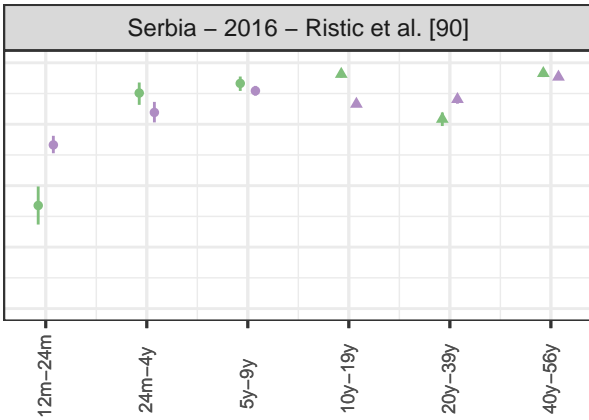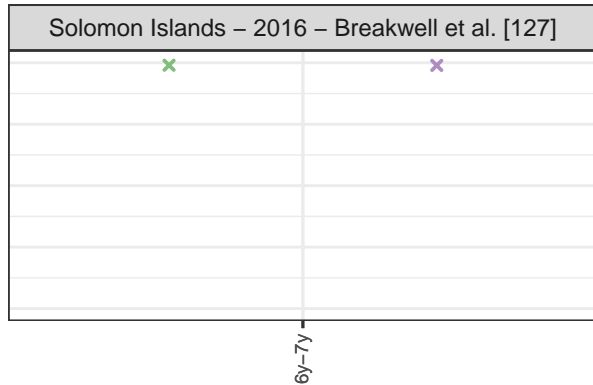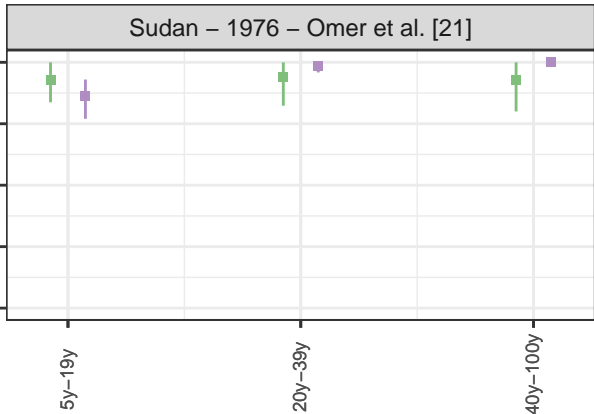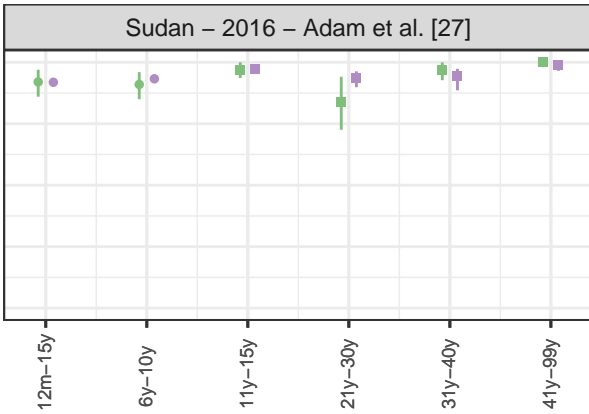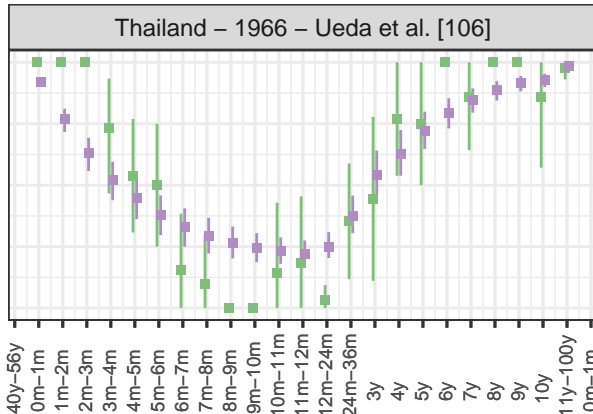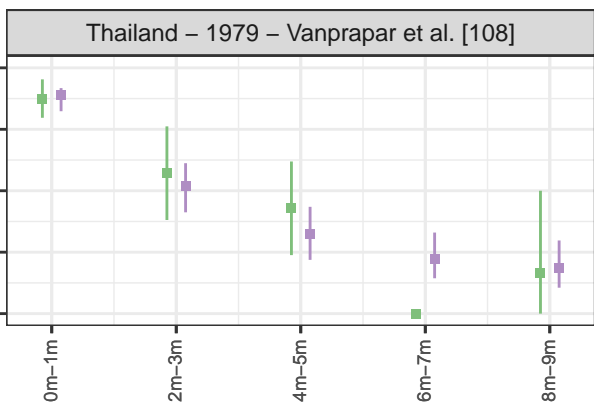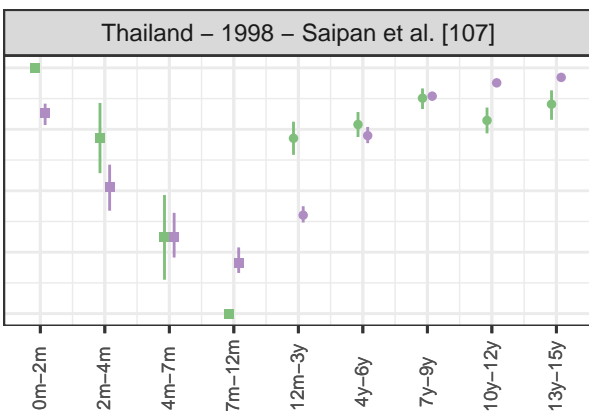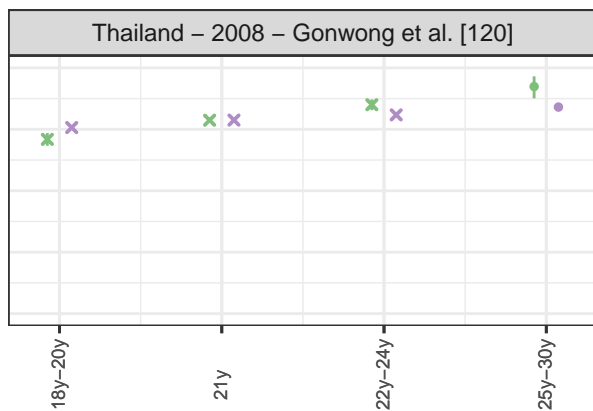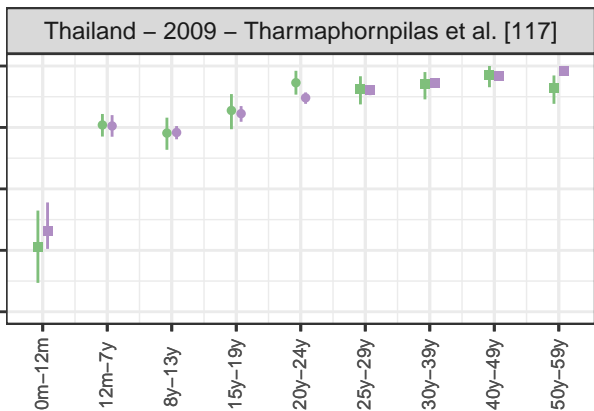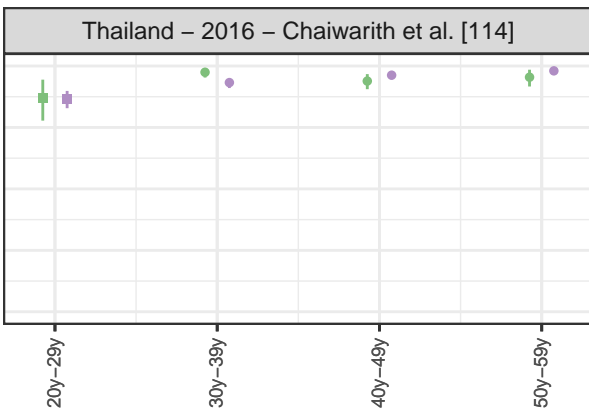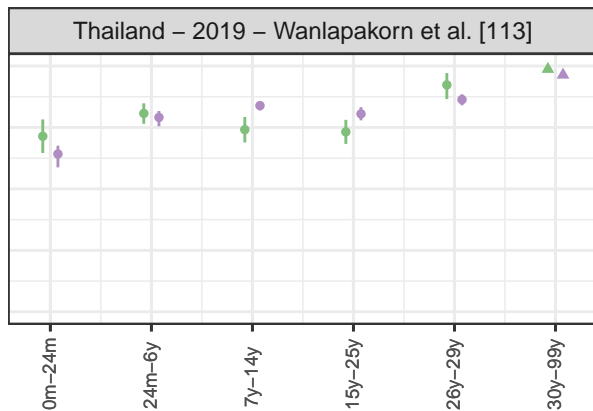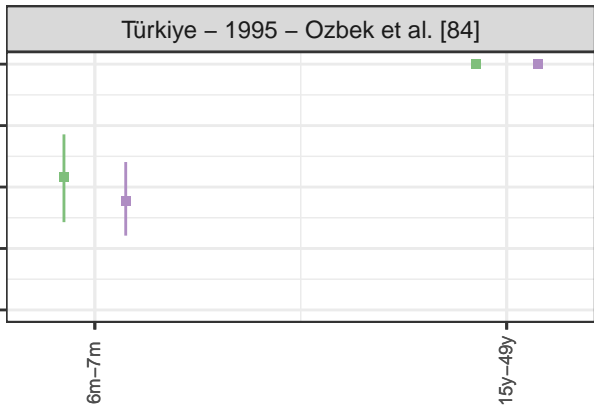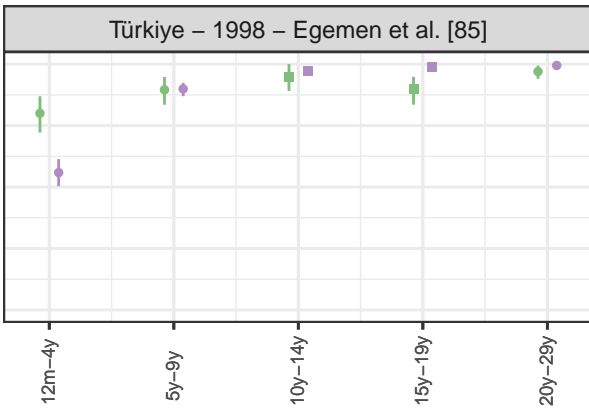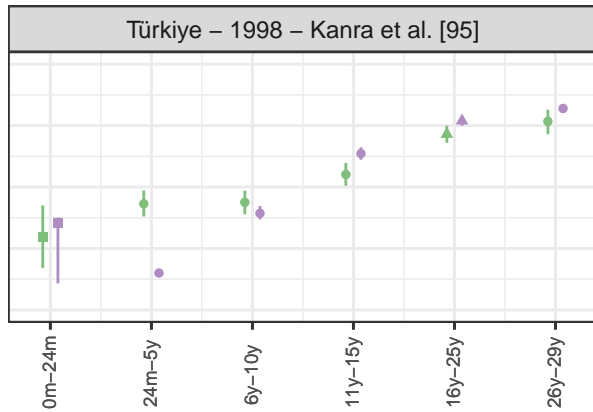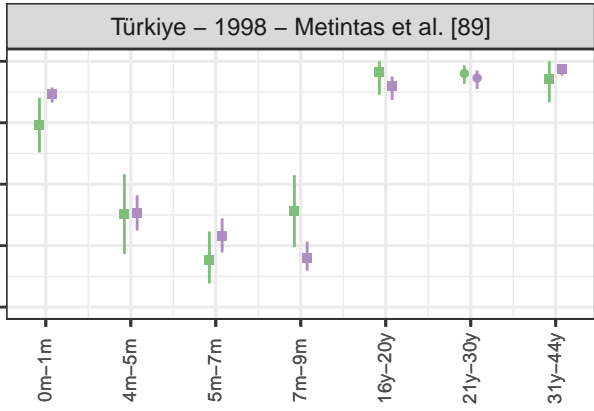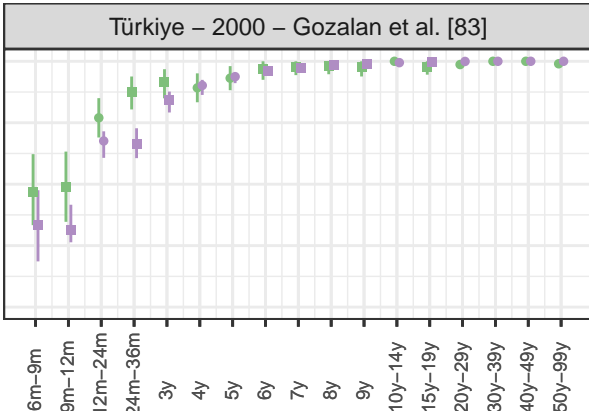

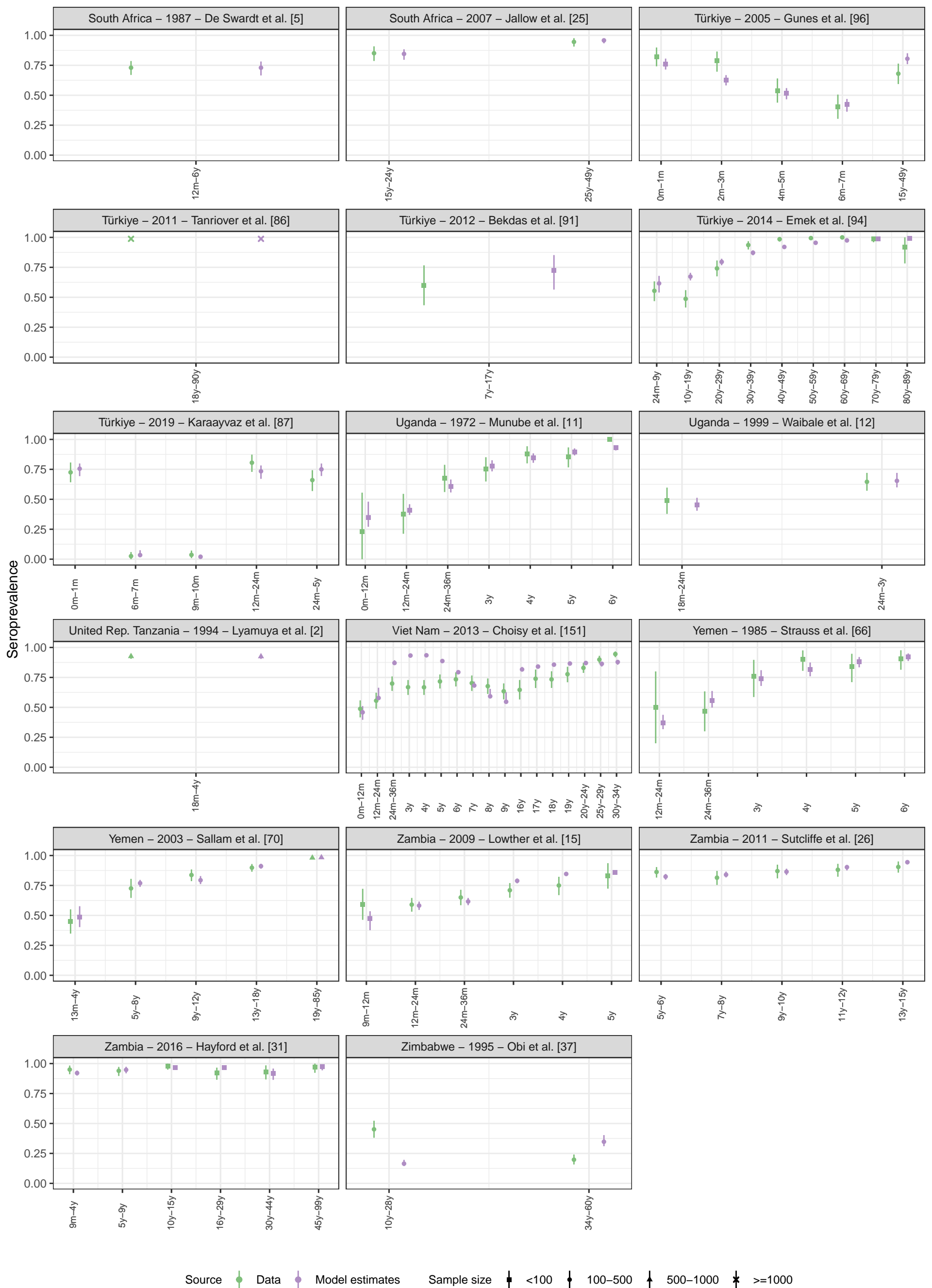

Supplement: S1 Fig — Study reference is included in S1 Appendix. (PDF) [file pgph.0006731.s002.pdf]
